# Fate of isoprene peroxy radical constrains the urban photochemical regime

**DOI:** 10.1126/sciadv.aea6509

**Published:** 2026-05-13

**Authors:** Michael A. Robinson, Matthew M. Coggon, Kelvin H. Bates, Jeff Peischl, Christopher M. Jernigan, Gordon Novak, Subi Thakali, James M. Roberts, J. Andrew Neuman, Patrick R. Veres, Kristen Zuraski, Eleanor M. Waxman, Wyndom S. Chace, Andrew W. Rollins, Victoria Treadaway, Morgan Selby, Colby Francoeur, Jessica B. Gilman, Shang Liu, Erin R. Delaria, Abby E. Sebol, Nidhi S. Desai, Jennifer Kaiser, Kathryn E. Kautzman, Jason M. St. Clair, Glenn M. Wolfe, Lu Xu, Chelsea E. Stockwell, Carsten Warneke, Han N. Huynh, Ming Lyu, Adam Ahern, Charles A. Brock, Alison Piasecki, Sarah Albertin, Ann M. Middlebrook, Amy P. Sullivan, Magesh Kumaran Mohan, Rodney Weber, Emily Lill, Ilana Pollack, Katherine Ball, John D. Crounse, Paul O. Wennberg, Anna Novelli, Aaron Stainsby, Hendrik Fuchs, Birger Bohn, Georgios I. Gkatzelis, Joshua P. DiGangi, Glenn S. Diskin, J. Jerrold M. Acdan, R. Bradley Pierce, Chia-Hua Hsu, Siyuan Wang, Rebecca Schwantes, Gonzalo González Abad, Caroline R. Nowlan, Xiong Liu, Nathan Howard, Steven S. Brown

**Affiliations:** ^1^ Cooperative Institute for Research in Environmental Sciences, University of Colorado, Boulder, CO 80305, USA.; ^2^ Chemical Sciences Laboratory, National Oceanic and Atmospheric Administration, Boulder, CO 80305, USA.; ^3^ Department of Chemistry, University of Wisconsin-Madison, Madison, WI 53706, USA.; ^4^ Department of Chemistry, University of Colorado, Boulder, CO 80305, USA.; ^5^ Department of Mechanical Engineering, University of Colorado, Boulder, CO 80305, USA.; ^6^ Department of Civil and Environmental Engineering, Northeastern University, Boston, MA 02115, USA.; ^7^ Atmospheric Chemistry and Dynamics Laboratory, NASA Goddard Spaceflight Center, Greenbelt, MD 20771, USA.; ^8^ Earth System Science Interdisciplinary Center, University of Maryland, College Park, MD 20740, USA.; ^9^ Department of Atmospheric & Oceanic Science, University of Maryland, College Park, MD 20742, USA.; ^10^ School of Earth and Atmospheric Sciences, Georgia Institute of Technology, Atlanta, GA 30332, USA.; ^11^ School of Civil and Environmental Engineering, Georgia Institute of Technology, Atlanta, GA 30332, USA.; ^12^ Department of Chemistry, Towson University, Towson, MD 21252, USA.; ^13^ Goddard Earth Sciences Technology and Research II, University of Maryland, Baltimore County, MD 21228, USA.; ^14^ Department of Atmospheric Science, Colorado State University, Fort Collins, CO 80521, USA.; ^15^ Division of Chemistry and Chemical Engineering, California Institute of Technology, Pasadena, CA 91125, USA.; ^16^ Division of Geological and Planetary Sciences, California Institute of Technology, Pasadena, CA 91125, USA.; ^17^ Division of Engineering and Applied Science, California Institute of Technology, Pasadena, CA 91125, USA.; ^18^ ICE-3: Troposphere, Forschungszentrum Jülich GmbH, Jülich, Germany.; ^19^ Department of Physics, University of Cologne, Cologne, Germany.; ^20^ NASA Langley Research Center, Hampton, VA 23681, USA.; ^21^ Department of Atmospheric and Oceanic Sciences, University of Wisconsin-Madison, Madison, WI 53706, USA.; ^22^ Space Science and Engineering Center, University of Wisconsin-Madison, Madison, WI 53706, USA.; ^23^ Atomic and Molecular Physics Division, Center for Astrophysics|Harvard & Smithsonian, Cambridge, MA 02138, USA.

## Abstract

Declining nitrogen oxide (NO*_x_* = NO + NO_2_) emissions have transformed oxidation pathways in urban atmospheres, with implications for air quality. Organic peroxy radicals (RO_2_), key intermediates in volatile organic compound oxidation, typically react with NO to form ozone (O_3_). Under lower-NO conditions, alternative RO_2_ fates, including isomerization forming highly oxidized organic molecules (HOMs), can enhance secondary organic aerosol (SOA) production. We combine aircraft observations over four major North American cities with geostationary satellite data to characterize isoprene-derived RO_2_ fate across urban environments. We infer RO_2_ bimolecular lifetimes (τ_bi_) as a proxy for isomerization potential, finding longer τ_bi_ (17 ± 11 seconds) in New York, Chicago, and Toronto compared to Los Angeles (7 ± 6 seconds). Satellite measurements reveal that long τ_bi_ is widespread across urban North America, suggesting that declining NO*_x_* is likely to lead to greater HOM formation in urban regions. These findings indicate that atmospheric models omitting RO_2_ isomerization chemistry may incorrectly simulate organic oxidation and the subsequent oxidation state of volatile organic compounds and SOA.

## Teaser

Less NO*_x_* shifts peroxy radical chemistry, reducing ozone but enhancing highly oxidized molecules.

## INTRODUCTION

The atmospheric chemical reactions between NO*_x_* = (NO + NO_2_), volatile organic compounds (VOCs), and HO*_x_* = (OH + HO_2_) produce secondary pollutants such as tropospheric ozone (O_3_) and secondary aerosol that degrade air quality and affect human health (*1*, *2*). The relative abundance of NO*_x_* and VOCs determines O_3_ and secondary aerosol production regimes, often described as either NO*_x_*-sensitive or NO*_x_*-saturated (*3*–*8*). These regimes depend on the fate of hydroxyl (OH), hydroperoxyl (HO_2_), and organic peroxy (RO_2_) radicals. Organic peroxy radicals formed by photochemical VOC oxidation react either with NO to form organic nitrates (RONO_2_) and alkoxy radicals (RO^•^), with NO_2_ to form acyl peroxynitrates [PANs; RC(O)OONO_2_], with HO_2_ to form hydroperoxides (ROOH), or with other RO_2_ to form an assortment of oxygenates. In addition, certain RO_2_ radicals may undergo isomerization to form highly oxidized organic molecules (HOMs) (*9*–*12*). The rates at which RO_2_ radicals react along these pathways, sometimes termed the photochemical regime, change the composition of atmospheric organic matter and affect the rate at which ozone, secondary organic aerosol (SOA), and other secondary pollutants form.

Defining photochemical regimes is challenging because of the nonlinear dependence of O_3_ and SOA production on NO*_x_* and VOCs. This understanding is essential for the development of air pollution mitigation strategies. Traditional approaches include model-based isopleth analyses (*6*, *13*–*19*), radical termination metrics (*17*, *19*–*24*), and proxies such as formaldehyde (HCHO)–to–NO_2_ ratio (FNR) or hydrogen peroxide (H_2_O_2_)–to–HNO_3_ ratio (*25*–*27*). FNR is useful for the HO*_x_*/NO*_x_* ratio because it is observable by satellite remote sensing instruments (*24*, *27*–*30*). While FNR does often correlate well with other photochemical regime indicators (*24*), it is imperfect because it is a quantity that integrates over time rather than representing the instantaneous RO_2_ fate, a key factor in the photochemical regime, particularly in transitional or low-NO regimes. Furthermore, the spatial and temporal variation in satellite-retrieved FNR does not represent the true extent of this heterogeneity in the chemistry or emissions (*31*).

Recent research underscores the importance of RO_2_ fate (i.e., low-NO or high-NO) in defining photochemical regimes. For example, organic molecules in wildfire plumes show a rapid shift from high-NO to low-NO oxidation as NO*_x_* is transformed to reactive nitrogen reservoirs (*32*). SOA modeling studies, such as those defining the fraction of RO_2_ reacting with either NO or HO_2_ (sometimes termed β), have shown that high-NO chemistry dominated the continental US in the early 2000s (*33*), while transitional chemistry (where RO_2_ + NO and RO_2_ + HO_2_ reactions compete) better represents the global atmosphere (*34*). Combined observations and models reveal widely varying RO_2_ fates in urban plumes (*35*) and the upper troposphere (*36*, *37*), highlighting the need for detailed observations.

As NO*_x_* emissions decline and RO_2_ fate shifts to predominately low-NO bimolecular fates, the prevalence of RO_2_ isomerization pathways will increase. RO_2_ isomerization is the overall process of an RO_2_ undergoing an intramolecular hydrogen shift to form an alkyl radical with a hydroperoxide functional group (*9*, *10*, *38*). This alkyl radical will rapidly form a more oxidized RO_2_, which can continue to undergo rapid H-shifts, or terminate to form diverse products including HOMs (*38*, *39*). Multiple generations of H-shifts to form HOMs are often termed autoxidation. Because of their low volatility, anthropogenic and biogenic HOMs can contribute substantially to SOA formation (*38*, *40*, *41*).

Isoprene, the largest biogenic VOC emission in the atmosphere, strongly influences the oxidative capacity of the terrestrial troposphere (*42*), alters NO*_x_* fate (*43*), and affects SOA (*44*–*46*). Isoprene reacts rapidly with OH and plays a key role in urban O_3_ exceedances in North America (*47*, *48*). In New York City (NYC), isoprene and other biogenic VOCs contribute up to 85% of O_3_ production from VOCs (*49*), while in Los Angeles, isoprene accounts for nearly half of O_3_ formation potential from VOCs in both 2014 and 2021 (*50*–*52*). Chicago, although less studied, also exhibits a large contribution of isoprene to OH reactivity (~30%) (*53*). Isoprene is ubiquitous in the air of most US cities, but its contribution to RO_2_ chemistry and O_3_ production is relevant anywhere isoprene chemistry is prevalent. Changes in anthropogenic VOC emissions, NO*_x_* emissions, climate, and urban greening could alter isoprene’s role in urban O_3_ production (*50*, *54*, *55*).

**Fig. 1.**
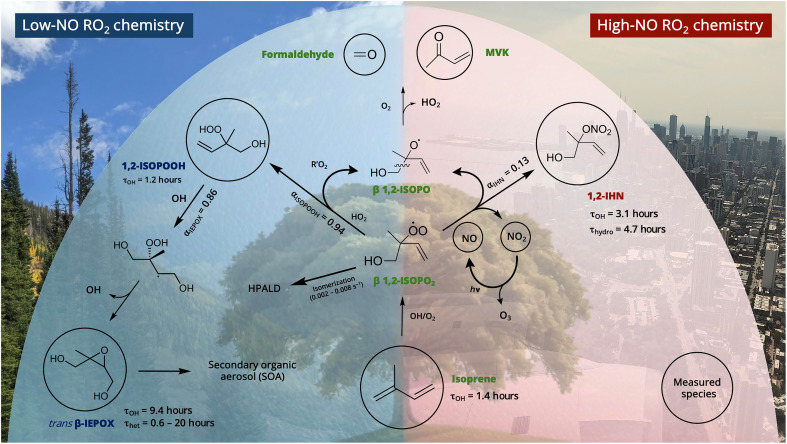
Reactions of (β-1,2) isoprene peroxy radical with NO, HO_2_, and RO_2_. Only the (β-1,2) isomer shown for clarity. Circled species were measured directly during SUNVEx and AEROMMA via CIMS (∑IHN and ∑ISOPOOH + IEPOX), LIF or cavity ring down spectroscopy (NO, NO_2_, and HCHO), and gas chromatography mass spectrometry (isoprene and MVK). Rates and branching ratios are from the study by Wennberg *et al.* (*57*). Background photos taken by P.R.V. and M.A.R.

Here, we leverage measurements of isoprene oxidation products and demonstrate a measurement-based approach to define the photochemical regime (Fig. 1). Specifically, we analyze measurements of isoprene hydroxy nitrates (IHNs; C_5_H_9_NO_4_) that are produced from RO_2_ + NO pathways and isoprene hydroperoxides and epoxy diols (ISOPOOH and IEPOX, respectively; C_5_H_10_O_3_) that are produced from RO_2_ + HO_2_ pathways. We show that the relative contribution of these products informs RO_2_ fate, photochemical regimes, and bimolecular lifetime for urban VOCs. We connect this measure to a satellite-derived proxy of photochemical regimes and show that RO_2_ isomerization, an important yet understudied determinant of atmospheric organic composition, is likely prevalent across most North American urban areas.

In the following sections, we first explore the use of isoprene RO_2_ as a proxy for understanding urban VOC RO_2_ fate. We then validate measurement-based isoprene RO_2_ fate against modeled metrics and define the chemical regimes governing O_3_ production. The discussion expands to examine the variation of chemical regimes across major North American cities sampled with aircraft during the summer of 2023. We study the role of RO_2_ isomerization in urban chemistry and lastly how chemical regimes observed with Tropospheric Emissions: Monitoring of Pollution (TEMPO) satellite, as indicated by measured column FNR, show the extent of RO_2_ isomerization in North American urban areas.

## RESULTS AND DISCUSSION

We evaluate relationships between isoprene RO_2_ fate, FNR, radical termination, and photochemical regime in urban air using data from two field campaigns. One was sampled at a ground site in Pasadena, CA (1 August to 5 September 2021; see section S1.1), downwind of the Los Angeles urban core. The other, part of the Atmospheric Emissions and Reactions from Megacities to Marine Areas (AEROMMA) mission, used a research aircraft to sample four major North America cities (26 July to 26 August 2023). Measurement and sampling details are provided in Materials and Methods and section S1.5.

### Estimating *f*_NO_ with isoprene RO_2_ and defining O_3_ production chemical regimes

**Fig. 2.**
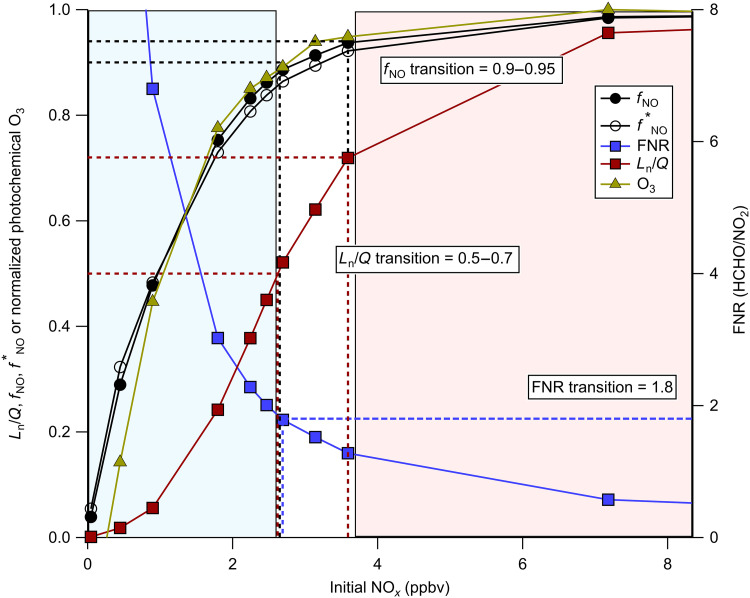
Modeled chemical regime transitions in isoprene RO_2_ chemistry. The model relationships of *f*_NO_, $f_{\text{NO}}^{*}$, *L*_n_/*Q*, FNR_in situ_, and normalized photochemical O_3_ production with initial NO*_x_* at constant OH exposure (2.3 × 10^10^ molecules cm^−3^ s) for isoprene chemistry. Blue shading represents NO*_x_*-sensitive chemistry, and red shading represents NO*_x_*-saturated chemistry. FNR_in situ_ and *L*_n_/*Q* transitions are presented from the literature (*20*, *24*, *31*, *59*), and *f*_NO_ and $f_{\text{NO}}^{*}$ transition is defined by *df*_NO_/*d*NO*_x_* < 0.1 ppbv^−1^.

Equation 1 defines the fraction of RO_2_ radicals reacting with NO, *f*_NO_

$$f_{\text{NO}}=\frac{k_{RO_{2}+\text{NO}}\left[\text{NO}\right]}{k_{RO_{2}+\text{NO}}\left[\text{NO}\right]+k_{RO_{2}+HO_{2}}\left[HO_{2}\right]+k_{RO_{2}+RO_{2}^{\prime }}\left[RO_{2}^{\prime }\right]+k_{\text{isom}}}$$ (1)

where $k_{RO_{2}+\text{NO}}$, $k_{RO_{2}+HO_{2}}$, $k_{RO_{2}+RO_{2}^{\prime }}$, and *k*_isom_ are the rate coefficients of RO_2_ reacting with NO, HO_2_, or $RO_{2}^{\prime }$, or isomerization, respectively. A similar parameter used previously (*56*), β, is defined when only considering the NO and HO_2_ bimolecular RO_2_ fates (see section S3.3) and represents *f*_NO_ when these reactants control RO_2_ reactive fate. We use known isoprene oxidation products of RO_2_ + NO (∑IHN) and RO_2_ + HO_2_ (∑[ISOPOOH+IEPOX]) pathways to develop a measurement-based proxy of *f*_NO_, termed $f_{\text{NO}}^{*}$, defined in Eq. 2

$$f_{\text{NO}}^{*}=\frac{\frac{\left[\text{IHN}\right]}{\alpha_{\text{IHN}}}}{\frac{\left[\text{IHN}\right]}{\alpha_{\text{IHN}}}+\left(\frac{\sum \left[\text{ISOPOOH}+\text{IEPOX}\right]}{\alpha_{\text{ISOPOOH}}\alpha_{\text{IEPOX}}}\right)}$$ (2)

where α_IHN_ and α_ISOPOOH_ are the product branching ratios (see Fig. 1; α_IHN_ = 0.13, α_ISOPOOH_ = 0.937, and α_IEPOX_ = 0.95) (*57*), and [IHN] and ∑[ISOPOOH+IEPOX] are measured mixing ratios using chemical ionization mass spectrometry (CIMS). Similar approaches have been used to understand the fate of RO_2_ in smoke using the known chemistry of VOCs emitted from biomass burning (*32*). In this analysis, isoprene is an ideal choice because (i) it is abundant and ubiquitous in urban, rural, and remote terrestrial regions and (ii) its product distributions are well studied. A full description of these CIMS measurements and the derivation of $f_{\text{NO}}^{*}$ is provided in Materials and Methods and sections S1.2 and S3.2. The uncertainty in $f_{\text{NO}}^{*}$ depends on its magnitude (see Materials and Methods and figs. S1 to S3). It is smallest when $f_{\text{NO}}^{*}$ is high (≈0.98, $\pm_{0.03}^{0.01}$), moderate at intermediate values (≈0.88, $\pm_{0.12}^{0.07}$), and largest at low values (≈0.63, $\pm_{0.22}^{0.19}$). Measurement-derived $f_{\text{NO}}^{*}$ relies on the assumption that production of isoprene oxidation products is large and that their measured relative concentration is not appreciably affected by differences in the rate of their chemical losses. We test these assumptions with individual AEROMMA flight box models over relevant chemical timescales (see sections S1.6, S2.2, and S2.3 and figs. S4). We apply models and ground site observations to define the *f*_NO_ transition point for NO*_x_*-saturated to NO*_x_*-sensitive O_3_ production. In Fig. 2, we compare the model relationships between *f*_NO_ and $f_{\text{NO}}^{*}$, as well as other proxies of atmospheric chemical regimes including FNR, radical termination (*L*_n_/*Q*), and chemical O_3_ production.

First, we evaluate *f*_NO_ for five urban VOCs using a box model constrained in Pasadena, CA, during the summer of 2021 to determine whether $f_{\text{NO}}^{*}$ derived from isoprene is representative of reactive fates of other VOCs observed in urban air (see section S2.1 for box model details). While VOC OH reactivity (VOCR; ∑*k*_OH_[VOC]) is driven by isoprene (45% of VOCR at noon) at the Pasadena ground site (fig. S5), we find that isoprene’s RO_2_ fate closely resembles those of other urban VOCs, including toluene, isopentane, and other biogenic VOCs, such as α-pinene (see section S3.4 and fig. S6). This is due to similarity in RO_2_ + NO and RO_2_ + HO_2_ rates across VOCs (*58*). We conclude that when isomerization and RO_2_ + $RO_{2}^{\prime }$ reactions are negligible, isoprene *f*_NO_ sufficiently represents urban VOC *f*_NO_ (fig. S6). However, for VOC-derived RO_2_ that isomerize rapidly, traditional bimolecular RO_2_ fate does not adequately describe their fate, an aspect we explore further below.

Figure 2 displays the nonlinear relationship isoprene *f*_NO_ has with NO*_x_*. Also shown is modeled $f_{\text{NO}}^{*}$, which agrees with *f*_NO_ within 15% and demonstrates the utility of this measurement-based proxy for RO_2_ fate (see figs. S7 and S8 and sections S2.1 to S2.3 for further comparisons and model descriptions). Notably, *f*_NO_ exhibits a monotonically increasing relationship with NO*_x_* until ~3.5 parts per billion by volume (ppbv). At this point (*f*_NO_ and $f_{\text{NO}}^{*}$ ~ 0.9), the proxy saturates and exhibits smaller sensitivity with respect to NO*_x_*. This transition occurs at the same NO*_x_* levels as the “chemical regime” transition typically associated with traditional O_3_ regime metrics, namely FNR and *L*_n_/*Q*. The radical termination approach, *L*_n_/*Q*, is calculated to estimate the O_3_ production chemical regime in both the Pasadena ground site and simplified isoprene box models (section S3.1) (*19*–*23*). Briefly, *L*_n_ is the rate of odd hydrogen (= OH + HO_2_ + RO_2_) removal by reactions with NO*_x_*, and *Q* is the sum of total odd hydrogen loss (see section S3.1 for more information). Values of *L*_n_/*Q* > 0.5 indicate NO*_x_*-saturated chemistry, where most of radical termination reactions proceed via reaction with NO*_x_* (*20*). Conversely, *L*_n_/*Q* < 0.5 indicates NO*_x_*-sensitive chemistry, where most of the radical termination reactions proceed via radical-radical reactions. However, in urban atmospheres when compared to ozone isopleths, the *L*_n_/*Q* transition value is typically higher (~0.7) (*31*, *59*). The transition in *f*_NO_ when $\frac{df_{\text{NO}}}{dNO_{x}}$ falls below 0.1 ppbv^−1^, corresponding to *f*_NO_ of 0.90, agreeing with in situ FNR (FNR_in situ_) and *L*_n_/*Q*, defined transitions in the literature (*L*_n_/*Q* = 0.5; FNR_in situ_ of 1.8) and other studies on *f*_NO_ (*20*, *24*, *36*, *37*). The transition in *f*_NO_ could be as high as 0.95 if *L*_n_/*Q* transition is 0.7, as shown in Fig. 2. Furthermore, we find that the average relative uncertainty for FNR_in situ_ is ±33% for the AEROMMA campaign (see Methods and Materials and figs. S1 to S3), emphasizing the uncertainty in the FNR_in situ_ transition definition (1.8 ± 0.6). Comparison of modeled $f_{\text{NO}}^{*}$ to *f*_NO_ in Pasadena shows agreement within 5 to 15% throughout the photochemical day (fig. S7). This comparison with real atmospheric data indicates a high bias in the model-derived transition of *f*_NO_, leading to defining the $f_{\text{NO}}^{*}$ chemical saturation at 0.9 with respect to O_3_ production regime. This saturation point does not reflect where the RO_2_ chemistry has equal NO and HO_2_ rates, which is at 0.5, but the point where RO_2_ fate and O_3_ production are no longer sensitive to NO.

### Chemical regimes across North American cities

**Fig. 3.**
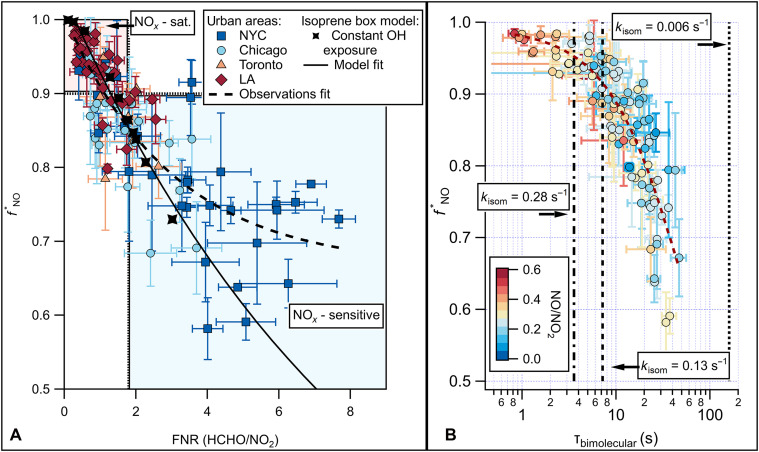
Observed relationships between $f_{\text{NO}}^{*}$, FNR, and RO_2_ bimolecular lifetime. Transect averaged (error bars indicate ±1 standard deviation) for measurement-based (**A**) $f_{\text{NO}}^{*}$ versus FNR and (**B**) $f_{\text{NO}}^{*}$ versus isoprene RO_2_ bimolecular lifetime, colored by the NO-to-NO_2_ ratio, for the sampled cities during the AEROMMA campaign. Reference isomerization lifetimes are shown for isoprene [*k*_isom_ = 0.006 s^−1^ (*93*)], α-pinene [*k*_isom_ = 0.28 s^−1^ (*70*)], and 2-ethoxy ethanol [*k*_isom_ = 0.13 s^−1^ (*69*)]. Exponential fits to the experimental data are shown in both panels.

Figure 3A shows transect-averaged $f_{\text{NO}}^{*}$ and FNR_in situ_ during AEROMMA for the four North American cities. Isoprene makes up 6 to 16% of VOCR in these cities, allowing for $f_{\text{NO}}^{*}$ determination (see fig. S9). The relationship between FNR_in situ_ and $f_{\text{NO}}^{*}$ illustrates how the established O_3_-production regime proxy (FNR) compares to the metric of RO_2_ fate. The O_3_-production regime transition for FNR_in situ_ is taken from Souri *et al.* (*24*) (1.8 ± 0.4), which agrees with the $f_{\text{NO}}^{*}$ saturation of 0.9 established above. However, column-derived FNR (FNR_remote_) transition values differ from FNR_in situ_. In the work of Duncan *et al.* (*27*), NO*_x_*-saturated chemistry occurs with FNR_remote_ < 1, and NO*_x_*-sensitive chemistry occurs with FNR_remote_ > 2, with transitional chemistry for FNR_remote_ between 1 and 2 (*60*, *61*). The simplified zero-dimensional box model–predicted results from Fig. 2 are also shown in Fig. 3A. The solid black curve and markers are model estimates at a constant OH exposure (AEROMMA average = 2.3 × 10^10^ molecules cm^−3^ s) created by varying initial NO from 100 to 3600 parts per trillion by volume (pptv) to span the range of measured $f_{\text{NO}}^{*}$ and FNR_in situ_. The agreement between the box model and field observations of FNR_in situ_ and $f_{\text{NO}}^{*}$ shows that the relationship is readily predictable up to FNR_in situ_ of ~4 with known chemistry if OH exposure is considered.

The RO_2_ lifetime with respect to bimolecular reactions with NO or HO_2_ or τ_bi_ (*9*, *10*, *12*, *34*, *62*) is also integral to this chemistry

$$\tau_{\text{bi}}=\frac{1}{k_{RO_{2}+\text{NO}}\left[\text{NO}\right]+k_{RO_{2}+HO_{2}}\left[HO_{2}\right]}$$ (3)

where $k_{RO_{2}+\text{NO}}$ is the bimolecular rate coefficient for isoprene RO_2_ reacting with NO (*57*), $k_{RO_{2}+HO_{2}}$ is the bimolecular rate coefficient for isoprene RO_2_ reacting with HO_2_ (*57*), [NO] is the observed NO number density measured on the DC-8, and [HO_2_] is the estimated HO_2_ number density from Weather Research and Forecasting model coupled with Chemistry (WRF-Chem) model runs sampled along the DC-8 flight track described in Materials and Methods. RO_2_ + $RO_{2}^{\prime }$ reactions are omitted because their bimolecular rates are typically slow (*63*–*65*). Modeled urban plume HO_2_ was on average 25 ± 9 pptv (see fig. S10). To evaluate WRF-Chem HO_2_, we calculate β and compare with $f_{\text{NO}}^{*}$, showing agreement within ±15% (see section S3.3 and fig. S11).

Figure 3A shows that observations of $f_{\text{NO}}^{*}$ from AEROMMA can mostly be explained with an isoprene box model or an exponential fit of the data (black dashed line). Transects that display NO*_x_*-saturated chemistry (upper left-hand sector of Fig. 3A) make up 38% of observations during AEROMMA and are predominately in the LA Basin or transects very close to urban centers. NO*_x_*-sensitive chemistry is plotted in the lower right-hand sector of Fig. 3A, comprising 36% of observations, mainly downwind of NYC, Chicago, and Toronto. The absence of $f_{\text{NO}}^{*}$ data below 0.55 and a small amount of points below 0.70 in Fig. 3A suggest that isoprene RO_2_ reactions with NO were the prevailing fate during AEROMMA, likely due to the campaign’s emphasis on sampling urban areas or the limited presence of low-NO oxidation conditions. The remaining 26% of observations fall in the lower left-hand sector of Fig. 3A, where $f_{\text{NO}}^{*}$ and FNR_in situ_ disagree in NO*_x_* sensitivity designation. This discrepancy may reflect regional differences in FNR_in situ_ threshold definition, uncertainty in FNR_in situ_, or cases where $f_{\text{NO}}^{*}$ does not fully represent the urban plume. These points are predominately from the Chicago transects (46%) and largely correspond to instances where $f_{\text{NO}}^{*}$ and β disagree outside of uncertainty ($f_{\text{NO}}^{*}$ uncertainty = $\pm_{0.12}^{0.07}$). However, nearly half of the observations in this sector (46%) fall within the uncertainty of their respective thresholds (FNR = 1.8 ± 0.6; $f_{\text{NO}}^{*}$ = 0.9 ± 0.1). In addition, FNR integrates both direct emissions and the accumulated effects of photochemistry, which can bias photochemical regime determination, whereas $f_{\text{NO}}^{*}$ reflects the prompt production of isoprene oxidation products, providing information on recent oxidation conditions.

NYC, Chicago, and Toronto all displayed a mix of chemical regimes during AEROMMA, with notable variability depending on the proximity to the urban core. NYC was predominately NO*_x_*-sensitive (69% of transects) with longer τ_bi_ (23 ± 12 s) and moderate $f_{\text{NO}}^{*}$ (0.79 ± 0.1), but Hudson River transects near Manhattan were NO*_x_*-saturated (see fig. S12D), with shorter τ_bi_ (6 ± 3 s) and high $f_{\text{NO}}^{*}$ (0.92 ± 0.03). Chicago’s plume exhibited transitional chemistry, split between NO*_x_*-sensitive (38% of transects) chemistry over Lake Michigan and NO*_x_*-saturated (19% of transects) chemistry near downtown, where τ_bi_ averaged 9 ± 3 s and $f_{\text{NO}}^{*}$ was 0.90 ± 0.03. Similarly, Toronto’s plume was characterized with τ_bi_ (13 ± 5 s) and $f_{\text{NO}}^{*}$ (0.87 ± 0.06), resembling Chicago. Across these cities, NO*_x_*-saturated chemistry persisted near urban centers, while NO*_x_*-sensitive regimes were found outside of the urban plume and downwind (fig. S12). However, this analysis reflects only the urban areas with sufficient observational constraints on FNR and $f_{\text{NO}}^{*}$ and does not capture broader, biogenically influenced regions with limited data, where lower-NO*_x_* conditions and different RO_2_ fates are expected.

In contrast, Los Angeles was dominated by NO*_x_*-saturated chemistry throughout the basin (73%), with only limited NO*_x_*-sensitive (8%) transects on the east side of the basin (see fig. S12A). As a result, the LA Basin exhibited the shortest isoprene RO_2_ lifetimes, with an average τ_bi_ of 7 ± 6 s and even shorter lifetimes downtown (3 ± 2 s). These transects closest to downtown LA had the highest $f_{\text{NO}}^{*}$ (0.96 ± 0.02) and lowest FNR_in situ_ (0.49 ± 0.2). Unlike NYC, Chicago, and Toronto, which all displayed mixed chemical regimes, LA’s consistently short τ_bi_, low FNR_in situ_, and high $f_{\text{NO}}^{*}$ emphasize the dominance of NO*_x_*-saturated chemistry (RO_2_ + NO reactions) across the basin at flight altitude.

### Role of RO_2_ isomerization in urban chemistry

Measured isoprene RO_2_ fate ($f_{\text{NO}}^{*}$) does not consider isomerization, but the τ_bi_ can be compared to RO_2_ isomerization lifetimes (τ_isom_ = 1/*k*_isom_) to infer the importance of this pathway. For example, although certain isoprene RO_2_ rapidly isomerize to form hydroperoxy aldehydes (HPALDs) (*9*, *62*), the bulk isoprene RO_2_ isomer pool isomerizes more slowly [*k*_isom,bulk_ ~ 0.002 to 0.008 s^−1^ (298 K)], with τ_bi_ ~ 160 s. This has implications for other VOCs for which RO_2_ isomerization can lead to the production of HOMs (*10*, *12*, *66*–*69*). We present our $f_{\text{NO}}^{*}$ as a function of estimated τ_bi_ in Fig. 3B. The average τ_bi_ estimated for isoprene during urban transects was 14 ± 10 s, ranging from 0.83 to 47 s. These values are much lower than those reported by Kenagy *et al.* (20 to 300 s) (*34*), reflecting the influence of the urban oxidation environment sampled during AEROMMA and the lack of observations under low-NO conditions that are more representative of the global background. However, the AEROMMA observationally constrained estimates of τ_bi_ are sufficiently long for isomerization chemistry to be an important chemical fate of other RO_2_ radicals in urban air, as proposed by Praske *et al.* (*12*).

The relatively slow bulk isomerization rate for isoprene RO_2_ means that a small fraction of these isomerize. Many RO_2_ derived from other VOCs have much faster isomerization rates than those from isoprene. For example, α-pinene and 2-ethoxy ethanol [*k*_isom,2EE_ = 0.13 s^−1^ (294 K); *k*_isom,apine_ = 0.28 s^−1^ (298 K); τ_isom,2EE_ = 7.7 s; τ_isom,apine_ = 3.6 s^−1^] (*66*, *68*–*71*) and even moderate RO_2_ bimolecular lifetimes (e.g., τ_bi_ > ~3 s) can lead to a substantial fraction of isomerization for certain VOCs. As will be shown below, moderate to high (τ_bi_ > ~3 s) RO_2_ bimolecular lifetimes are prevalent in urban regions across North America. The fraction of RO_2_ isomerizing (*f*_isom_) can be defined similarly to *f*_NO_ in Eq. 1

$$f_{\text{isom}}=\frac{k_{\text{isom}}}{1/\tau_{\text{bi}}+k_{\text{isom}}}$$ (4)

*k*_isom_ is for the first-generation RO_2_ isomer of interest (at 298 K), and isomerization rates are highly dependent on the VOC and temperature. Master Chemical Mechanism (version 3.3.1) RO_2_ + NO and RO_2_ + HO_2_ rate constant expressions are used in Eq. 4. In addition to isoprene, we examine the anthropogenic VOCs 2-ethoxy ethanol and hexanal [*k*_isom,hexanal_ = ~0.2 s^−1^ (298 K)] and α-pinene as important biogenic SOA precursors (*69*, *72*, *73*). These VOCs span key source sectors, including biogenic emissions, volatile chemical products, and cooking, and have either modeled or measured RO_2_ isomerization rates. We report only the fraction of RO_2_ that isomerize at the rates reported above to account for large differences in RO_2_ isomer–specific isomerization rates (i.e., only certain RO_2_ isomers isomerize fast enough for this chemistry to matter). In the case of α-pinene, the total *f*_isom_ is weighted by the fraction of RO_2_ formed that isomerize rapidly (22%) (*73*). The estimated *f*_isom_ from AEROMMA data varied not only between VOCs but also between cities. The LA Basin had the lowest fraction on average because of the short τ_bi_ (isoprene: 4 ± 3%; 2-ethoxyethanol: 28 ± 14%; hexanal: 35 ± 15%; α-pinene: 12 ± 4%). The cities with longer τ_bi_ (NYC, Chicago, and Toronto) displayed much higher average isomerization fractions (isoprene: 9 ± 5%; 2-ethoxyethanol: 44 ± 11%; hexanal: 51 ± 10%; α-pinene: 17 ± 3%). We calculate *f*_isom_ for typical conditions during AEROMMA, and it is apparent that isomerization chemistry will become more prevalent with decreasing NO (see fig. S13). These isomerization processes lead to lower-volatility HOMs, which have been shown to contribute to SOA formation (see section S3.8 and table S2) (*38*, *40*, *41*, *73*).

### Chemical regimes observed by TEMPO

**Fig. 4.**
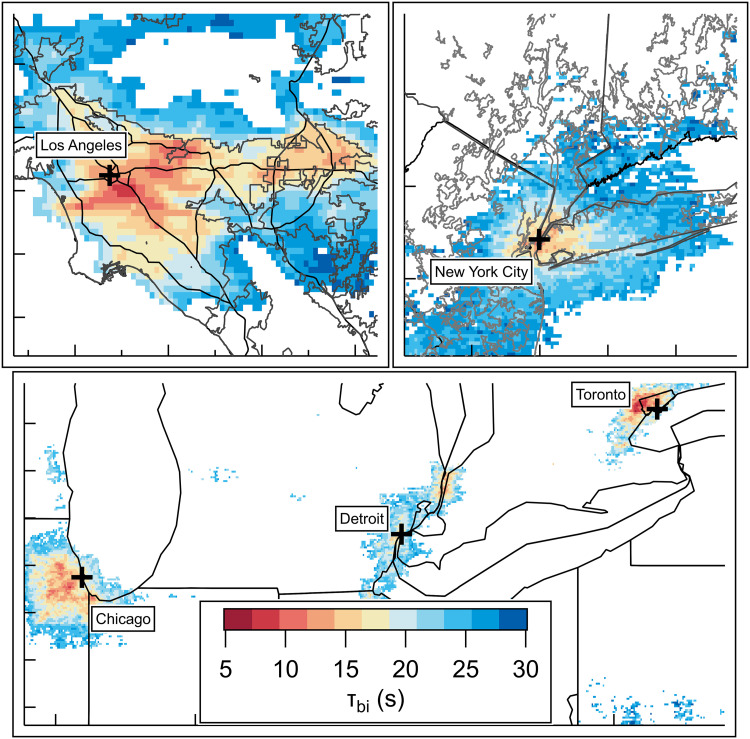
Satellite mapping of RO_2_ bimolecular lifetime using in situ constraints. TEMPO satellite retrieval for 1 p.m. to 5 p.m. PDT average for the month of August 2023 of τ_bi_ as estimated with the relationship to FNR_in situ_ determined during AEROMMA. Data are filtered on the basis of the published thresholds for HCHO and NO_2_ (see Materials and Methods).

Tropospheric Emissions: Monitoring of Pollution (TEMPO; first light August 2023 during AEROMMA) retrieves tropospheric NO_2_ and HCHO columns hourly over North America. Average August 2023 maps of TEMPO FNR_remote_ and $f_{\text{NO}}^{*}$ derived from correlations between FNR_in situ_ and $f_{\text{NO}}^{*}$ are shown in fig. S14 and S15. TEMPO hourly data are averaged for the entire month of August 2023 from 1300 to 1700 PDT (Pacific daylight time), which overlaps with typical aircraft sampling hours. Exponential fits to $f_{\text{NO}}^{*}$ and τ_bi_ versus FNR_in situ_ in urban plumes during AEROMMA are used to produce fig. S15 and Fig. 4 (see figs. S16 and S17). Data filtering applied to FNR_remote_ (see Materials and Methods) in Fig. 4 largely excludes regions of North America without high tropospheric NO_2_ or HCHO columns, highlighting urban areas. FNR_in situ_ values from the DC-8 during AEROMMA agree in magnitude, and spatial distribution with monthly averaged TEMPO retrieved FNR_remote_ values in most cases (see figs. S18 to S20). AEROMMA-derived τ_bi_ maps of sampled cities are shown in Fig. 4. As was shown with the AEROMMA data, urban areas share similar FNR_remote_, $f_{\text{NO}}^{*}$, and τ_bi_ values across North America, indicating a widespread prevalence of RO_2_ isomerization. TEMPO enables near-real-time, continental-scale constraints on chemical regimes, but interpretation of satellite-derived proxies requires appropriate context. In this work, we demonstrate correlation between $f_{\text{NO}}^{*}$, τ_bi_, and FNR_in situ_ (fig. S16 and S17), providing a chemistry-based linkage to FNR_remote_. Extension of these relationships to TEMPO FNR_remote_ introduces uncertainty because the retrieval is column integrated, is temporally averaged, and reflects chemistry integrated over several hours. Accordingly, TEMPO-derived regime patterns should be interpreted as broad, regional indicators rather than instantaneous local constraints, within which this analysis provides observationally constrained maps of τ_bi_ at the continental scale.

These data show that isomerization for certain VOCs is a widely prevalent RO_2_ fate in urban environments, which likely affects organic composition. Current state-of-the-art models (e.g., Goddard Earth Observing System–Chem, Community Regional Atmospheric Chemistry Multiphase Mechanism) have only recently included this chemistry (*73*–*75*), necessitating careful model and measurement comparisons. As was shown in Fig. 3B, the relationship between *f*_NO_ and τ_bi_ predicts an increasing importance in RO_2_ isomerization reactions as NO mixing ratios continue to decrease. While decreasing NO*_x_* levels are predicted to decrease OH and offset SOA from isomerization products (*73*), for α-pinene, 2-ethoxy ethanol, and hexanal, the prevalence of RO_2_ isomerization reactions in urban areas, at current NO*_x_* and OH levels, is already high. This widespread occurrence of RO_2_ isomerization and its known temperature dependence may contribute to the formation of HOMs, which in turn could be a contributing factor in SOA formation in NYC and LA along with other processes, such as wildfire influences or temperature-dependent VOC emissions (*76*–*79*). In addition, it has been recently shown that the nationwide decreasing NO*_x_*/VOC ratio, an indicator of the reaction partner of OH (i.e., to make RO_2_ or to form HNO_3_), has an important impact on the NO*_x_* loss, increasing the importance of RO_2_ + NO compared to OH + NO_2_ → HNO_3_ (*80*). These findings highlight the importance of incorporating more RO_2_ isomerization chemistry in reduced chemical mechanisms to accurately predict future air quality trends in a changing NO*_x_* landscape.

## MATERIALS AND METHODS

### Aircraft measurements and 2023 campaign description

The AEROMMA mission used the instrumented NASA DC-8 research aircraft during the summer of 2023 to sample urban pollution plumes from four North American cities (see fig. S21) (*81*). Isoprene oxidation products (∑IHN and ∑[ISOPOOH + IEPOX]) were measured by the National Oceanic and Atmospheric Administration (NOAA) I^−^ CIMS and California Institute of Technology CF_3_O^−^ CIMS (see fig. S22) (*82*, *83*). The NOAA I^−^ CIMS was calibrated after the campaign in the same manner as described by Robinson *et al.* (*84*), but isomer distributions were updated with flight-by-flight box models (described below) representative of aircraft sampling conditions. Additional details of the NOAA I^−^ CIMS measurements are shown in section S1.2.

In addition to isoprene oxidation products, NO*_x_*, aerosol pH, aerosol liquid water (ALW), total aerosol surface area, methyl vinyl ketone (MVK), and methacrolein (MACR) were used to constrain the heterogeneous losses of isoprene oxidation products and to estimate average isoprene system OH exposure. NO*_x_* was measured via a custom two-channel NO laser-induced fluorescence (LIF) instrument with a blue light converter to measure NO_2_ (*85*). Formaldehyde (HCHO) was measured via a custom LIF instrument (*86*). Nitric acid (HNO_3_) was measured with the California Institute of Technology CF_3_O^−^ CIMS (*82*, *83*). MVK and MACR were measured using a custom-built whole air canister system analyzed by gas chromatography–mass spectrometry (*87*). Each canister was analyzed by custom, two-channel gas chromatography–mass spectrometry with a 20-min duty cycle per sample. Canister samples are analyzed in series using quadrupole mass spectrometry (Agilent 5975C). Samples were analyzed postflight, typically within 48 hours of sampling. OH reactivity was measured by flash photolysis and LIF (*88*). Aerosol pH and ALW were determined by ISORROPIA-lite run in forward mode, constrained by high-resolution aerosol mass spectrometer nonrefractory mass concentrations, nonvolatile cations measured by ion chromatography of particle-into-liquid samples, gas phase NH_3_ and HNO_3_, relative humidity, and temperature (see fig. S23). The total aerosol volume and surface area (see fig. S24) were derived from a complete size-resolved aerosol composition profile and adjusted for ambient relative humidity. Additional details of measurements are shown in section S1 and table S3.

### $f_{\text{NO}}^{*}$ and FNR uncertainty

We quantified uncertainties in both $f_{\text{NO}}^{*}$ and FNR using a Monte Carlo approach that propagates measurement and yield uncertainties. We use nominal measurements (IHN, ∑[ISOPOOH + IEPOX], HCHO, and NO_2_) and product branching ratios from the literature (α_IHN_, α_ISOPOOH_, and α_IEPOX_). Each parameter was assigned a log-normal uncertainty distribution defined by its nominal value and reported uncertainty (see table S3 for measurement uncertainties). Because of the wide-ranging mixing ratios observed during AEROMMA, we take three cases (LA, Chicago, and NYC) spanning the range of measurements. For each case, we draw 2 × 10^5^ Monte Carlo samples, evaluate $f_{\text{NO}}^{*}$ and FNR, and report the resulting probability distribution, mean, and 95% confidence interval (*89*, *90*). The probability distribution functions and associated uncertainties for both $f_{\text{NO}}^{*}$ and FNR for each case are shown in figs. S1 to S3.

### Isoprene oxidation timescales and corrections

We apply a sequential reaction model to estimate the OH exposure that isoprene and its products experienced, following approaches used previously from field data (*91*, *92*). This sequential reaction model (see section S2) uses MVK and MACR ratios to isoprene to estimate isoprene system OH exposures, which ranged from 1.1 × 10^10^ to 5.1 × 10^10^ molecules cm^−3^ s (average ± standard deviation: 2.3 ± 0.7 × 10^10^ molecules cm^−3^ s) (fig. S4) during AEROMMA urban sampling. Anthropogenic VOC OH exposure (average ± standard deviation: 8.3 ± 2.6 × 10^10^ molecules cm^−3^ s) is higher than isoprene OH exposures during AEROMMA. The same sequential reaction model can be applied to the ground site observations in Pasadena to estimate isoprene OH exposure, which ranged from 0.15 × 10^10^ to 3.4 × 10^10^ molecules cm^−3^ s (average ± standard deviation: 0.95 ± 0.5 x 10^10^ molecules cm^−3^ s) (fig. S4). We use these measured isoprene system OH exposures to evaluate box models to the appropriate chemical timescales as well as correct ∑[ISOPOOH + IEPOX] signal.

We apply a correction to the ∑[ISOPOOH + IEPOX] signal measured by I^−^ CIMS to correct for differences in sensitivity for each isomer (see section S1.4). The fraction of ISOPOOH is determined flight by flight (see fig. S25) from individual isoprene box models constrained to meteorology (temperature, pressure, and photolysis) and parameters affecting heterogenous loss processes (ALW, aerosol pH, aerosol surface area, and aerosol mean radius) and evaluated at isoprene OH exposures observed across each flight (see section S2.3). The impact of this isomer distribution on I^−^ CIMS sensitivity was found to be ±15%, well within typical CIMS uncertainties (see fig. S26).

We use $f_{\text{NO}}^{*}$ to estimate production rates of ∑IHN and ∑[ISOPOOH + IEPOX], which relies on short oxidation timescales (where OH dictates loss processes). A high background of either IHN or ∑[ISOPOOH + IEPOX] could skew $f_{\text{NO}}^{*}$ in the direction of the high-background molecule. As there is very little NO outside of the urban plumes measured, the ∑IHN background is generally quite low during AEROMMA and SUNVEx. Because of HO_2_ being an important bimolecular reaction partner outside of urban air, we have implemented a background subtraction for ∑[ISOPOOH + IEPOX] mixing ratios in both the AEROMMA and SUNVEx datasets (see section S1.7, table S4, and fig. S27).

### HO_2_ estimate from the regional chemical model

An estimate of HO_2_ was derived from WRF-Chem, sampled along the DC-8 flight track (see fig. S10). Three WRF-Chem simulations were conducted to cover the AEROMMA spatial and temporal domain, one centered on Chicago, one centered on NYC (which included Toronto), and one covering the conterminous United States for LA flights. Detailed descriptions of these WRF-Chem setups can be found in section S2.4. HO_2_ has little impact on the determined τ_bi_ and β for moderate to high NO (NO >100 pptv; see fig. S28).

### FNR TEMPO data

We build FNR_remote_ maps from L3 TEMPO qualified data (main_data_quality_flag = 0) and filtered for effective cloud fraction <0.2, with tropospheric NO_2_ (https://doi.org/10.5067/IS-40e/TEMPO/NO2_L3.003) and total HCHO (https://doi.org/10.5067/IS-40e/TEMPO/HCHO_L3.003) version 3 columns. On the basis of FNR_remote_ error quantification and validation work done for TROPOMI satellite retrieval products, we have disregarded data below a threshold of 2.7 × 10^15^ molecules cm^−2^ for both tropospheric NO_2_ and HCHO columns. This is based on the work of Souri *et al.* (*24*), adjusted for monthly averaging (i.e., scaled by 1/√*n*).

## Supplementary Materials

### This PDF file includes:

Supplementary Text Figs. S1 to S30 Tables S1 to S6 References
