# Supplementary material for: Fate of isoprene peroxy radical constrains the urban photochemical regime
Source: Sci Adv. 2026 May 13;12(20):eaea6509. doi: 10.1126/sciadv.aea6509 (PMC13170639; doi:10.1126/sciadv.aea6509)
Supplement: Supplementary file 1 — Supplementary Text Figs. S1 to S30 Tables S1 to S6 References [file sciadv.aea6509_sm.pdf]

Science Advances
AAAS

Supplementary Materials for

Fate of isoprene peroxy radical constrains the urban photochemical regime

Michael A. Robinson *et al.*

Corresponding author: Michael A. Robinson, michael.a.robinson@noaa.gov

Sci. Adv. **12**, eaea6509 (2026)
DOI: 10.1126/sciadv.aea6509

This PDF file includes:

Supplementary Text
Figs. S1 to S30
Tables S1 to S6
References

1 Measurements

1.1 Ground Site Measurements and 2021 Pasadena, CA Ozone Photochemistry Campaign Description:

The Southwest Urban NO_x and VOC Experiment (SUNVEx) (https://csl.noaa.gov/projects/sunvex/) was an intensive air quality study conducted in the summer of 2021. Trace gas measurements were made at a ground site at the California Institute of Technology campus in Pasadena, CA from 1 August to 5 September. The location of this ground site was in close proximity to the ground site used during the 2010 CalNex campaign (*94*). Trace gas measurements were made from a 10m tall tower (*95*). This ground site has been evaluated as a downwind receptor site for the Los Angeles urban plume, and has been shown to be routinely impacted by these emissions and photochemistry (*96*).

Isoprene oxidation products during SUNVEx were measured using the NOAA iodide time of flight chemical ionization mass spectrometer (NOAA I^- CIMS) and a gas chromatography mass spectrometer (GC-MS) (*87*, *97*, *98*, *84*). The NOAA I^- CIMS has been described in detail elsewhere(*97*, *98*, *84*), but in short analytes react with iodide (I^-) or iodide water clusters ($[I{\bullet}H_2O]^-$) and are detected as iodide analyte clusters using a modified time of flight mass spectrometer (*99*, *100*) with a mass resolving power of approximately 5000 (Δm/m). Reagent ions are made by flowing methyl iodide (CH_3I) in front of a vacuum ultraviolet Krypton lamp (*98*, *84*, *101*, *102*). Iodide CIMS measured isoprene oxidation products included ∑IHN ($[I{\bullet}C_5H_9NO_4]^-$; m/z: 273.958) and ∑[ISOPOOH + IEPOX] ($[I{\bullet}C_5H_{10}O_3]^-$; m/z: 244.968), which were both calibrated for isomer specific sensitivities and reported with yield weighted instrument response factors using a box modeling approach (*84*). In addition to the isoprene oxidation products, HNO_3 and acyl peroxynitrates (PANs), were measure by the NOAA I^- CIMS, providing important constraints to the box model and radical termination budget. The iodide water cluster is controlled in the ion molecule reactor (IMR) region of the instrument via a small saturated N_2 flow to maintain a cluster ratio ($[I{\bullet}H_2O]^-/I^-$) of 0.5 ± 0.004 during the day and 0.57 ± 0.01 at night. In order to measure PANs (PAN, PPN, APAN, fur-PAN), a thermal dissociator inlet made of PFA tubing set to 130°C was used (*103*).

In addition to oxidation products, measurements of VOCs and NO_x were used to constrain the box model at the Pasadena, CA ground site. NO_x was measured via a custom two channel NO- laser induced fluorescence (LIF) instrument with a blue light converter to measure NO_2 (*85*). Ozone was measured with UV-absorption (*104*). Benzene, toluene, benzaldehyde, methanol, acetaldehyde, ethanol, and the sum of monoterpenes were measured using a proton-transfer-reaction mass spectrometer (*105–107*). Formaldehyde was measured with cavity ring down spectroscopy (CRDS) (*108*, *109*). Isoprene, ethene, ethane, propane, isobutane, n-butane, iso-pentane, n-pentane, n-hexane, n-decane, ethylbenzene, 1,2,4-trimethyl-benzene, 1,3,5-trimethyl-benzene, acetone, MVK and MACR were measured with GC-MS (*87*). Carbon monoxide and methane were measured with CRDS(*108*). The photolysis rate of NO_2 was measured with a filter radiometer (*110*). More information about ground site measurements can be found in SI Table 6.

1.2 AEROMMA I^- CIMS Measurements:

The NOAA I^- CIMS was deployed on the NASA DC-8 with an improved IMR designed to be less susceptible to large changes in aircraft cabin temperature, which may impact instrument sensitivity (*98*). In short, this version of the NOAA IMR, mimics the volume and residence time of the previous generation of NOAA IMR (*98*), but adds robust temperature control with a water jacket and thermoelectric chiller (ThermoTek) set to maintain an IMR temperature of 30°C. The cluster ratio was controlled to 0.55 ± 0.03 for this campaign to span the difference in the marine and urban ambient water vapor conditions. Instrument sensitivity was tracked in flight and over the course of the field campaign by standard additions at the aircraft inlet of formic acid ($[I{\bullet}HCOOH]^-$; m/z 172.911) from a custom permeation source and isotopically labelled PAN, measured at both the acid anion ($^{13}C_2H_3O_2^-$; m/z: 61.021) and peroxy radical cluster ($[I{\bullet}^{13}C_2H_3O_3]^-$; m/z: 203.920)(*111*). The ^{13}C PAN standard is mass shifted from the target analyte and was added continuously during every flight. The HCOOH permeation source output was added every hour during flight on zero air or ambient air. On aircraft access days, a Br_2 ($[I{\bullet}^{79}Br^{81}Br]^-$; m/z: 286.740) permeation source was also used to track instrument sensitivity over the course of the campaign.

1.3 AEROMMA Aerosol Measurements:

In order to calculate heterogeneous and hydrolysis loss rates described in SI Section 2.3 we determine total wet aerosol surface area and average radius, aerosol liquid water (ALW) and aerosol pH. ALW and aerosol pH were determined by ISORROPIA-lite run in forward mode, constrained by HR-AMS non-refractory mass concentrations, the PiLS-IC non-volatile cations, gas phase NH_3 and HNO_3, relative humidity and temperature. Aerosols were provided to the HR-AMS from outside the aircraft with an NSF/NCAR High-performance Instrumented Airborne Platform for Environmental Research (HIAPER) modular inlet (HIMIL) that was previously characterized for the DC-8 (*112*). A constant mass flow rate into the HR-AMS was maintained with a pressure-controlled inlet (*113*). Data were obtained at 1 Hz, in cycles of 5 s for background correction, 42 s for bulk aerosol sampling, and 12 s for sampling aerosol mass distributions. The uncertainty on the total AMS mass concentrations is estimated to be from 20 to 35% (*114*). However, there is a significant, positive bias for the AEROMMA AMS field data when compared to separate measurements of the aerosol size distribution.

Aerosol surface area is measured dry and converted into a total wet size distribution by mapping speciated chemical composition from the high resolution Aerosol Mass Spectrometer (HR-AMS) (bulk particle composition: 100 nm – 5 µm) (*114*) and PALMS-NG (single particle composition: 120 nm - 5 µm) (*115*, *116*) onto the available aerosol size distribution (3 nm - 5 µm) measured by NMASS (*117*, *118*), UHSAS (Droplet Measurement Technologies) (*119*) and CMASS (PALAS) instruments, sampling as described in Brock et al. 2019 (*120*). This aerosol mapping process for the urban environment expands on Brock et. al. 2021 (*121*).

1.4 I^- CIMS ISOPOOH and IEPOX sensitivity correction:

Individual flight box models are initialized with isoprene and NO_x mixing ratios tuned to best match IHN mixing ratios observed in urban plume transects. Once $f_{ISOPOOH}$

($= \frac{ISOPOOH}{\sum[ISOPOOH+IEPOX]}$) is determined for each iWAS sample, the CIMS sensitivity to $\sum$[ISOPOOH + IEPOX] can be determined:

$$Sens_{\sum ISOPOOH+IEPOX} = f_{ISOPOOH}(OH_{exposure}) * \mathrm{Sens_{ISOPOOH}} + \left(1 - f_{ISOPOOH}(OH_{exposure})\right) * Sens_{IEPOX} \quad (1)$$

We apply an average sensitivity factor for each flight based on all of the iWAS samples collected on that flight (typically 144 samples). All individual flight box model $f_{ISOPOOH}(OH_{exposure})$ are shown in SI Figure 25. While there is significant range of predicted $f_{ISOPOOH}$ (10 to 22 % at average OH exposure) over the 14 urban flights analyzed, the impact on instrument sensitivity is small. Individual CIMS $\sum$[ISOPOOH + IEPOX] sensitivities for each iWAS sample are shown in SI Figure 26 for all 14 research flights, while CIMS sensitivity changes from sample to sample due to changing OH exposure, a single average sensitivity of 18.7 ± 2.8 nHz $pptv^{-1}$ can describe the entire urban sampling portion of the campaign.

1.5 AEROMMA Sampling approach:

This analysis focuses on the urban portion of the AEROMMA campaign, where 14 research flights sampled four North American megacities, New York City (4 flights), Chicago (5 flights), Toronto (2 flights) and Los Angeles (3 flights) from 26 July to 26 August 2023 (see SI Figure 21). These flights were designed to evaluate VOC and reactive nitrogen emissions, chemistry, and their influences on O_3 and aerosol formation. The NASA DC-8 was based in Dayton, OH to study eastern megacities (NYC, Chicago and Toronto) from 26 July to 18 August 2023. The aircraft was then based out of Palmdale, CA from 19 August to 26 August 2023 to study the Los Angeles and the San Joaquin Valley. We utilize all urban flights to characterize the O_3 photochemical regimes, which include flights on weekdays and weekends as well as days with contrasting low and high photochemical activity. The aircraft typically took off between 10:30 AM to 13:30 PM from Dayton, OH, with flight durations lasting ~6.5 hours. Take off time in Palmdale, CA was 11:00 AM for two research flights and one 13:30 PM flight to target evening chemistry with a flight duration of ~ 7.3 hours. Typically, the aircraft was on scene during the height of photochemical O_3 production (12:00 to 16:00 local time) and executed two to three 'circuits' of a predetermined flight plan, which were designed to sample the plume under typical flow patterns for each city.

1.6 Isoprene oxidation products lifetimes:

First and second generation products of isoprene oxidation may be lost by reactions with OH, uptake onto aerosols, or deposition to surfaces (*57*). Due to the possible differing lifetimes of IHN, ISOPOOH, and IEPOX in sampled plumes, it is critical to quantify these lifetimes and make appropriate corrections to $\sum$[ISOPOOH + IEPOX] before calculating and interpreting f^*_{NO}.

Assuming a constant daytime OH number density of 3 x 10^6 molec cm^{-3}, the OH lifetime of IHN ((β-1,2-IHN): 3.1 hr; (β-4,3-IHN): 2.2 hr), ISOPOOH ((1,2-ISOPOOH): 1.2 hr; (4,3-ISOPOOH): 0.8 hr) are similar magnitude, while IEPOX OH loss is slower by a factor of two or more ((*trans-β*-IEPOX): 9.4 hr; (*cis-β*-IEPOX): 6 hr) (see SI Table 5). Additionally, as IEPOX is directly produced from ISOPOOH – OH chemistry, OH exposure largely dictates the ISOPOOH – IEPOX isomer distribution, if heterogenous

and deposition losses are small. Due to IEPOX OH lifetimes being long, heterogeneous uptake of IEPOX largely dictates its lifetime. Depending on aerosol properties (particle acidity, sulfate) IEPOX heterogenous lifetimes can vary dramatically from 0.6 – 20 hr (*122*, *33*). However, with declining sulfate mass fraction in $PM_{2.5}$ due to SO_2 emission controls, heterogeneous loss of IEPOX has declined and will likely continue to decline (*123*). Rapid IHN hydrolysis has been shown to play an important role in the fate of (1,2)-IHN in Pasadena, CA (*124*). At this ground site, the (1,2)-IHN isomer hydrolysis lifetime ranged from 4 – 7 hr. The other major IHN isomer (4,3) loss process is linear with ALW and hydrolysis rates are somewhat uncertain (*124*).

1.7 ISOPOOH and IEPOX background correction:

As HO_2 is not generally low outside of urban plumes and is an important RO_2 bimolecular reaction partner when NO is low, this makes for conditions where the ∑[ISOPOOH+IEPOX] mixing ratio outside of the plume is often observed to be a significant portion of the ∑[ISOPOOH+IEPOX] signal measured in the plume. During AEROMMA for each plume transect we estimate a constant ∑[ISOPOOH+IEPOX] background and subtract from the in plume ∑[ISOPOOH+IEPOX] mixing ratio. Individual plume transects ∑[ISOPOOH+IEPOX] backgrounds are tabulated in SI Table 4. This can also impact ground site determination of f^*_{NO}, if significant amounts of ∑[ISOPOOH+IEPOX] or ∑IHN remain overnight and elevate the effective background of diurnally produced isoprene oxidation products. During SUNVEx, ∑[ISOPOOH+IEPOX] often did not go to zero at night (average night time mixing ratio: 11.8 ± 5 pptv), so a high order polynomial was fit to the nighttime mixing ratios (SI Figure 27) and subtracted from the daytime data used for determining f^*_{NO}.

2 Models

2.1 Description of constrained box model at Pasadena ground site:

We simulated organic nitrate isomer distributions at the Pasadena ground site using the Framework for 0-D Atmospheric Modeling, a photochemical zero-dimensional box model (*125*). The gas phase chemical mechanisms implemented in this 0-D model are the Master Chemical Mechanism (MCMv3.3.1).

The model was initialized using the hourly averaged measured mixing ratios of NO, NO_2, O_3, CO, CH_4, PAN, PPN, HONO, N_2O_5, $ClNO_2$, C_5H_8 (isoprene), monoterpenes (α-pinene, β-pinene, limonene), C_2H_4 (ethene), C_2H_6 (ethane), C_3H_8 (propane), i-C_4H_{10} (isobutane), n-C_4H_{10} (n-butane), i-C_5H_{12} (iso-pentane), n-C_5H_{12} (n-pentane), n-C_6H_{14} (n-hexane), n-$C_{10}H_{22}$ (n-decane), benzene, toluene, ethylbenzene, 1,2,4-trimethyl-benzene, 1,3,5-trimethyl-benzene, benzaldehyde, HCHO, acetaldehyde, acetone, methanol, ethanol, methyl vinyl ketone, and methacrolein.

We assume ALW of 5 $\mu g\ m^{-3}$ for parameterization of IHN loss (described below in Section 2.3), $\gamma_{IEPOX} = 2.0 \times 10^{-4}$, aerosol surface area of 125 $\mu m^2\ cm^{-3}$ and mean particle radius of 150 nm.

2.2 Isoprene sequential reaction model:

The isoprene sequential reaction model has been used in literature elsewhere (*91*), briefly it relies on a simple reaction scheme where a parent molecule (i.e. isoprene) reacts in the atmosphere to form a known product (i.e. MVK or MACR) at a known rate, k_1,

with a known branching ratio, α_1, and with known loss rate of product species, k_2. An expression of the ratio of [Product]/[Parent] can be solved for analytically:

$$\frac{[Product]}{[Parent]} = \frac{\alpha_1 k_1}{(k_2 - k_1)}\{1 - exp(k_1 - k_2)t\} \quad (2)$$

The system of reactions in the isoprene system are well characterized, with rates and yields that are well known:

$$isoprene + OH \rightarrow 0.23MACR + 0.33MVK$$

$k_1 = 1.0 \times 10^{-10}$ cm^3 molec^{-1} s^{-1}

Daytime removal of MACR and MVK are dominated by the reaction with OH:

$MACR + OH \rightarrow products$ $\quad$ $k_2 = 2.9 \times 10^{-11}$ cm^3 molec^{-1} s^{-1}

$MVK + OH \rightarrow products$ $\quad$ $k_3 = 2.0 \times 10^{-11}$ cm^3 molec^{-1} s^{-1}

Because the reaction with O_3 and photolysis loss are very slow compared to OH loss for both MVK and MACR, they are neglected and the isoprene-MVK-MACR system can be described by just OH loss, shown below for the MVK/isoprene:

$$\frac{[MVK]}{[isoprene]} = \frac{0.23 k_1}{(k_3 - k_1)}\{1 - exp(k_1 - k_3)[OH]t\} \quad (3)$$

Where [OH]t is defined as the OH exposure experienced by the parent-product compounds.

This model relies on fixed starting concentration of isoprene at t=0 and there is negligible MVK or MACR at t=0. We believe this assumption applies to detached plumes in the atmosphere but at ground sites it may be susceptible to bias due to air containing isoprene and oxidation products. However, because we are concerned with daytime planetary boundary layer, we believe 'leftover' isoprene or oxidation products will be quite small relative to same-day isoprene and products, so we ignore these. During SUNVEx at the Pasadena ground site near zero mixing ratios of isoprene MVK and MACR were measured (see SI Figure 29).

2.3 AEROMMA individual flight box models:

In order to generate corrections and test the assumption of ∑IHN and ∑[ISOPOOH+IEPOX] loss rates are similar, we model each AEROMMA flight with an isoprene box model, which is initialized with NO_x in photo stationary steady-state, isoprene, OH (3×10^6 molec cm^{-3}), HO_2 estimated from WRF-Chem, observations of jNO_2, pressure, temperature, relative humidity, aerosol surface area, mean particle radius, and ALW (see SI Table 1). We assume solar zenith angle of 25 and a background O_3 mixing ratio of 65 ppbv. We determine IEPOX uptake coefficient from modeled the aerosol pH (see SI Section 1.3). The heterogenous loss of IEPOX is parameterized as a first order rate constant ($k_{IEPOX,het}$):

$$k_{IEPOX,het} = \left(\frac{r_p}{D_g} + \frac{4}{w\gamma}\right)^{-1} * SA \quad (4)$$

Where SA is the total wet aerosol surface area (see SI section 1.3), r_p is the mean particle radius, D_g is the gas diffusion coefficient, w is the mean molecular speed, and γ is the IEPOX reaction probability. The IEPOX reaction probability, γ_{IEPOX}, varies strongly with particle acidity and sulfate content. We use γ_{IEPOX} parameterization from GEOS-Chem ($1 < pH < 2$: 1.1×10^{-3}; $2 < pH < 3$: 2.0×10^{-4}) (*33*). The aerosol pH during AEROMMA for all urban flights except 20230809 NYC was between 2 and 3, yielding a γ_{IEPOX} of 2.0×10^{-4}.

In addition to IEPOX uptake, we implement IHN hydrolysis in our box model. The hydrolysis loss of IHN is parameterized as a first order rate constant ($k_{IHN,het}$) in the box model as:

$$k_{IHN,het} = ALW * k^{*}_{hydro} \quad (5)$$

Where ALW is the aerosol liquid water content and k^{*}_{hydro} (M atm^{-1} s^{-1}) is the product of the Henry's law coefficient (K_H) and the condensed phase hydrolysis rate ($k_{(aq)}$). Here we use flight by flight ALW (see SI Section 1.3) and effective hydrolysis coefficients derived by Vasquez et al. (*124*) from GEOS-Chem simulations (k^{*}_{hydro} = 3 x 10^5 M atm^{-1} s^{-1} for (1,2)-IHN and 0.31 M atm^{-1} s^{-1} for (4,3)-IHN).

The box model used in Figure 1 to generally describe relationships of f_{NO}, f^{*}_{NO}, FNR, O_3 and L_n/Q was configured with the 20230826 LA flight heterogenous chemistry. This simplified isoprene model was initialized with [OH] of 3 × 10^6 molec. cm^{-3}, [HO_2] from WRF-Chem, 2 ppbv of isoprene, and variable NO_x (45 pptv – 28 ppbv in photo stationary state).

2.4 WRF-Chem simulations:

HO_2 concentrations for the Los Angeles AEROMMA flights were temporally and spatially interpolated from WRF-Chem version 4.2.2 model simulations setup with a domain covering the conterminous United States at a horizontal resolution of 12 × 12 km^2 and with 50 vertical layers from the surface to 50 hectopascals. The model setup used the RACM-ESRL-VCP mechanism described in Zhu et al., 2024 (*126*) with in brief the following updates: The lateral boundary and initial conditions are from the NCEP North American Mesoscale 12 km Analysis for meteorology and Realtime Air Quality Modeling System for chemistry. The anthropogenic emissions are from The GReenhouse gas And Air Pollutants Emissions System (GRA^2PES, https://csl.noaa.gov/groups/csl4/gra2pes/). The fire emissions are developed based on the fire radiative power data from the Regional hourly Advanced Baseline Imager and the Visible Infrared Imaging Radiometer Suite Emissions (RAVE) product (*127*) and the smoke emissions coefficients from Stockwell et al. 2022 (*128*). The biogenic and soil NO_x emissions are from the inline Biogenic Emissions Inventory System in WRF-Chem, with reference isoprene emissions input reduced by 25% following He et al. (2023) (*129*).

We utilized the Weather Research and Forecasting model coupled with Chemistry (WRF-Chem) version 4.5.1 (*130*, *131*) to conduct two regional-scale air quality simulations centered on the Chicago and New York City metropolitan areas. The New York City modeling domain also includes the Toronto metropolitan area. Each modeling domain has a 4-km × 4-km horizontal spatial resolution and 41 vertical levels extending from the surface up to 50 hectopascals (hPa). The modeling period began on 21 July 2023 and ended on 17 August 2023, with output generated every one hour.

The WRF-Chem simulations implemented the T1_MOZCART chemistry and aerosol scheme, which utilizes the Model for Ozone and Related chemical Tracers (MOZART) with an updated tropospheric chemistry mechanism to simulate gas phase chemistry (*132*) and the Goddard Chemistry Aerosol Radiation and Transport (GOCART) model to simulate aerosol processes. The Tropospheric Ultraviolet and Visible (TUV) photolysis option was selected to parameterize photochemical processes.

Additionally, the aerosol radiative feedback option was turned on, which allowed aerosols to impact the radiation schemes and meteorological processes within the model.

Meteorological initial and boundary conditions were provided by the High-Resolution Rapid Refresh (HRRR) model. Chemical initial and boundary conditions were provided by the Whole Atmosphere Community Climate Model (WACCM) version 6. For anthropogenic emissions, we used the 2017 base year 1-km Neighborhood Emission Mapping Operation (NEMO) dataset, upscaled to 4-km, and point sources from the U.S. EPA 2017 National Emissions Inventory. Biogenic emissions were provided by the Model of Emissions of Gases and Aerosols from Nature (MEGAN) version 2.1. Biomass burning emissions were provided by the Fire INventory from NCAR (FINN) version 1.5.

For comparison to aircraft-based observations and use in subsequent analyses, the WRF-Chem simulation results were interpolated in space and time to match the location and sampling frequency (1 Hz) of the DC-8 observations for all Chicago, New York City, and Toronto flight days of the AEROMMA field campaign. These "model flight track" files are available for download in the AEROMMA data archive (https://csl.noaa.gov/projects/aeromma/data.html).

3 Observations describe photochemical regime

3.1 Radical termination approach (L_n/Q):

We use an observationally constrained box model at an urban receptor ground site (Pasadena, CA) to define the O_3 photochemical production regimes. We identify a five day O_3 event during SUNVEx in which average diurnals profiles of VOCs, NO_x, and meteorological conditions (pressure, temperature, relative humidity and photolysis rates) are used to constrain the box model (*84*). More information on the constrained box model can be found in SI Section 2.1. Here the L_n/Q calculation is compared against modeled and measured proxies for RO_2 fate (f_{NO}, f^*_{NO}, $HCHO/NO_2$). L_n is the rate of odd hydrogen loss (= $OH + HO_2 + RO_2$) to nitrogen through the following reactions:

$OH + NO_2 \rightarrow HNO_3$
$RO_2 + NO \rightarrow RONO_2$
$RO_2 + NO_2 \rightarrow RO_2NO_2$

And Q is the rate of all odd hydrogen loss, including radical-radical reactions:

$RO_2 + HO_2 \rightarrow ROOH$
$HO_2 + HO_2 \rightarrow H_2O_2 + O_2$
$OH + HO_2 \rightarrow H_2O + O_2$

In the Pasadena ground site box model, L_n/Q implies NO_x-saturated chemistry during the morning (see SI Figure 7; 5 am to 10 am). However, after 10 am, the interpretation of chemical regimes garnered from L_n/Q and f_{NO} differ. L_n/Q displays a rapid decrease towards NO_x-sensitive chemistry, while f_{NO} remains high suggesting the presence of high-NO RO_2 chemistry in this regime. The difference in these two metrics stems from L_n/Q fully describing radical termination reactions impacting both sides of the O_3 production curve, and f_{NO} describing only the NO_x-sensitive side of the O_3 production curve. RO_2 radicals are central in O_3 production chemistry and their fate controls an increasingly important portion of the chemical regime under NO_x-sensitive conditions.

3.2 Definition and derivation of f^*_{NO}

We define a measurement-based approach to estimating the fraction of isoprene RO_2 reacting with NO (f^*_{NO}):

$$f^*_{NO} = \frac{\frac{[IHN]}{\alpha_{IHN}}}{\frac{[IHN]}{\alpha_{IHN}} + \left(\frac{\sum[ISOPOOH+IEPOX]}{\alpha_{ISOPOOH}\alpha_{IEPOX}}\right)} \tag{6}$$

Here, α_{IHN} and $\alpha_{ISOPOOH}$ are the product branching ratios (see Figure 1; $\alpha_{IHN} = 0.13$, $\alpha_{ISOPOOH} = 0.937$, $\alpha_{IEPOX} = 0.95$) (*57*), and [IHN] and ∑[ISOPOOH+IEPOX] are measured mixing ratios using chemical ionization mass spectrometry. The derivation of this metric is provided below:

$ISOPO_2 + NO \rightarrow IHN$ k_1, α_1

$ISOPO_2 + HO_2 \rightarrow \sum ISOPOOH + IEPOX$ k_2, α_2

k_1 and k_2 are bimolecular rate coefficients

α_1 and α_2 are yields in each respective channel

α_2 is the product of $ISOPO_2 + HO_2 \rightarrow ISOPOOH$ and $ISOPOOH + OH \rightarrow IEPOX$ yields

Define k'$_1$ and k'$_2$:

$$k_1' = k_1[NO] \quad \text{1st order}$$

$$k_2' = k_2[HO_2] \quad \text{1st order}$$

Implicit assumption is:

[NO] >> $ISOPO_2$

[HO_2] >> $ISOPO_2$

K' = k'$_1$ + k'$_2$

$$\frac{d[IHN]}{dt} = pIHN = \alpha_1 k_1'[ISOPO_2] \tag{6}$$

$$\frac{d[\sum ISOPOOH+IEPOX]}{dt} = p\sum ISOPOOH + IEPOX = \alpha_2 k_2'[ISOPO_2] \tag{7}$$

$$\frac{d[ISOPO_2]}{dt} = -K'[ISOPO_2] \tag{8}$$

$$[ISOPO_2] = [ISOPO_2]_0 e^{-K't} \tag{9}$$

$$\frac{d[IHN]}{dt} = \alpha_1 k_1'[ISOPO_2]_0 e^{-K't} \tag{10}$$

$$[IHN] = -\alpha_1 \frac{k_1'}{K'}[ISOPO_2]_0 e^{-K't}\Big|_0^t$$

$$= \alpha_1 \frac{k_1'}{K'}[ISOPO_2]_0\left(1 - e^{-K't}\right) \tag{11}$$

$$\sum[ISOPOOH + IEPOX] = \alpha_2 \frac{k_2'}{K'}[ISOPO_2]_0\left(1 - e^{-K't}\right) \tag{12}$$

We define f^*_{NO} from observations:

$$f^*_{NO} = \frac{\frac{[IHN]}{\alpha_1}}{\frac{[IHN]}{\alpha_1} + \frac{\sum[ISOPOOH + IEPOX]}{\alpha_2}}$$

Substituting (11) and (12):

$$f^*_{NO} = \frac{\frac{k_1'}{K'}[ISOPO_2]_0\left(1-e^{-K't}\right)}{\frac{k_1'}{K'}[ISOPO_2]_0\left(1-e^{-K't}\right)+\frac{k_2'}{K'}[ISOPO_2]_0\left(1-e^{-K't}\right)} = \frac{k_1'}{k_1'+k_2'} \tag{13}$$

Recall:

$$f_{NO} = \frac{k_{RO2+NO}[NO]}{k_{RO2+NO}[NO]+k_{RO2+HO2}[HO_2]} = \frac{k_1'}{k_1'+k_2'} \tag{14}$$

3.3 Definition of β:

$$\beta = \frac{k_{RO_2+NO}[NO]}{k_{RO_2+NO}[NO] + k_{RO_2+HO_2}[HO_2]} \tag{15}$$

Where k_{RO2+NO} is bimolecular rate constant for RO_2 reacting with NO (*57*), $k_{RO2+HO2}$ is the bimolecular rate constant for RO_2 reacting with HO_2 (*57*), [NO] is the observed NO number density measured on the DC-8 and [HO_2] is the estimated HO_2 number density from WRF-Chem described in the SI Section 2.4. The agreement between our measured f^*_{NO} and the estimated β (SI figure 11) is within ± 15%.

3.4 Isoprene RO_2 estimates urban RO_2 fates:

We utilize five common urban VOCs (ethene, MVK, i-pentane, toluene, and α-pinene) due to their prevalence in urban air (together they make up 13% of VOCR at 8 am) and because they span a relevant range of carbon numbers and RO_2 + HO_2 reaction rates compared to the isoprene-RO_2 system. Calculated f_{NO} for all parent VOCs exhibit similar behavior when NO mixing ratios are high (i.e., NO > 1 ppbv; SI Figures 6 and 7; 5 to 10 am) due to the similarity in NO and HO_2 rate coefficients in Eq. 1 (*63*). It is worth noting that the RO_2 + HO_2 rate coefficient is thought to increase by a factor of 1.9 from ethene to pentane derived RO_2 in the MCM v3.3.1.

3.5 Comparison of f^*_{NO} and f_{NO}:

We compare the measured f^*_{NO} to modeled f_{NO} in a simplified 0-D box model. We find good agreement between measures (see SI Figure 8). This agreement stems from loss process timescales. During AEROMMA, ∑[ISOPOOH+IEPOX] had a modeled-average lifetime of 9.8 ± 0.6 hrs controlled by OH due to small heterogeneous loss rates (average aerosol pH = 2.1 ± 0.5; $\gamma_{IEPOX} \sim 2.0 \times 10^{-4}$; see SI Figure 23). Whereas, ∑IHN had a modeled-average lifetime of 2.1 ± 0.3 hrs, which was strongly influenced by hydrolysis loss on wet aerosol (average aerosol liquid water = 6.6 ± 3 μg m^{-3}; see SI Figure 23 and Table 1). This difference in lifetimes means for the AEROMMA data after isoprene has experienced 3 - 4 hrs of OH exposure f^*_{NO} and f_{NO} begin to diverge, due to hydrolysis loss dictating ∑IHN lifetime, rather than OH loss controlling ∑IHN and ∑[ISOPOOH+IEPOX] lifetimes.

3.6 Example flight tracks of f^*_{NO} display evolving photochemical regimes spatially and temporally:

The ground site data from Pasadena, CA during SUNVEx shows RO_2 fate during peak O_3 production is largely driven by high-NO chemistry ($f^*_{NO} > 0.9$, SI Figure 7). The LA basin flights during AEROMMA (SI Figure 12a) are consistent with ground site measurements made in Pasadena during SUNVEx – i.e. f^*_{NO} indicates high-NO chemical regime in the majority of the basin during peak O_3 production. Data acquired throughout the entire LA basin demonstrates that this chemical regime from 1400 to 1600 PDT. Downwind transects near San Bernardino indicate shifting RO_2 chemistry with f^*_{NO} decreasing to 0.8, implying $RO_2 + HO_2$ are becoming an important reaction pathway in this region (80% RO_2 + NO, 20% $RO_2 + HO_2$). This is supported by other analysis of the LA basin showing the O_3 production is predominately NO_x-saturated close to LA and Pasadena, but trends towards transitional chemistry outside of the urban core and in downwind locations towards the east in San Bernadino (*133*). Toronto, shown in SI Figure 12b displays similar RO_2 chemistry to LA – i.e., where the second and third transect downwind of the urban area displayed $f^*_{NO} > 0.9$ at the plume center, indicating high-NO RO_2 chemistry is prevalent in this urban plume. However, the first downwind plume transects did not display consistent wind direction, with winds shifting from downtown Toronto to off Lake Ontario, possibly explaining the lower f^*_{NO} (< 0.8) values measured closest to the urban center. The Chicago plume, shown in SI Figure 12c, sampled on 8/12/23 exhibits transitional or high-NO RO_2 chemistry immediately downwind of the urban center, with f^*_{NO} values ranging from 0.87 – 0.91, with the second downwind transect displaying transitional to low-NO RO_2 chemistry represented by f^*_{NO} values ranging from 0.76 – 0.89. The NYC plume, shown in SI Figure 12d, sampled on 8/16/23, exhibits similar high-NO RO_2 chemistry as LA during the Hudson River transect closest to the urban core in Manhattan. The f^*_{NO} values ranged from 0.89 – 1.0, some of the highest measured over the entire campaign. These observations of urban core high-NO RO_2 chemistry are reinforced by an analysis of the NYC plume during the 2018 O_3 season, which showed that O_3 production was NO_x – saturated up to 40 km downwind of Manhattan (the urban center of NYC) (*49*).

3.7 Transect correlation of f^*_{NO} and formaldehyde to NO_2 in urban plumes:

Formaldehyde to NO_2 (FNR) is a long established proxy for O_3 photochemical regime (*24*, *27*, *29*). Ease of monitoring these species and the ability to retrieve both from ultraviolet–visible viewing satellites, make FNR an attractive representation for O_3 photochemical regime quantification. However, due the chemical complexity of the dependence of HCHO production on NO_x (*92*) and the possible impact of primary HCHO emissions, we compare FNR to the chemically constrained proxy, f^*_{NO}. As we have shown in Figure 2, there is a clear inverse relationship between these measures, and together we may infer information about the fundamental chemistry in plumes measured during AEROMMA. There is a clear anti-correlation of FNR and f^*_{NO}, where f^*_{NO} increases towards high-NO chemistry in the plume center while FNR decreases towards NO_x–saturated O_3 production chemistry (see SI Figure 30). In this case, these proxies of O_3 production regime are anti-correlated. The top panel in SI Figure 30 shows FNR that < 1.8 in the center of the Toronto urban plume. This threshold (1.8) is typical of NO_x–saturated chemistry (*24*). In agreement, the f^*_{NO} measure of chemical regime, shown in

the top panel of SI Figure 30, indicates the isoprene RO_2 radicals experienced distinctly high-NO chemistry ($f^*_{NO} > 0.9$). This relationship between FNR and f^*_{NO} chemical regime classification make understanding the relationship between the two consequential in interpreting field observations.

3.8 α-pinene autoxidation fraction in particle calculation:

Not all isomerization pathways lead to HOM, often autoxidation is needed, but in all sampled AEROMMA cities we observe α-pinene gas-phase HOM tracers with iodide CIMS. In order to predict the fraction of autoxidation products partitioned to the particle we use C* of first- and second-generation autoxidation products determined by Pye et al. (*73*). Fraction in particle can be determined with the following equation:

$$f_p = \left(1 + \frac{C_i^*}{C_{OA}}\right) \quad (16)$$

Where C_i* is the saturation vapor concentration of the molecule of interest and C_{OA} is the organic aerosol concentration. Here we assume 10.5 µg m^{-3} based on AEROMMA AMS data. *C** data is tabulated for HO_2 and NO termination products of alpha-pinene autoxidation RO_2 in SI Table 2. It is clear from these calculations that in the α-pinene system, first generation HOM is not fully partitioned to the particle (fraction in particle (f_p): 3.90 to 99.7%), but second-generation HOM from autoxidation will fully partition to the particle (f_p: 96.1 to 100.0%).

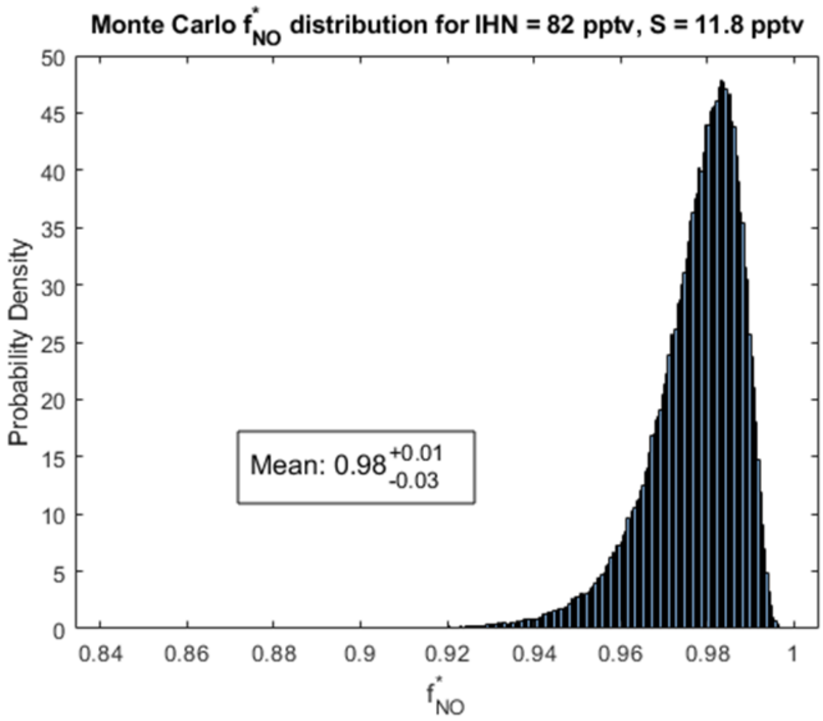

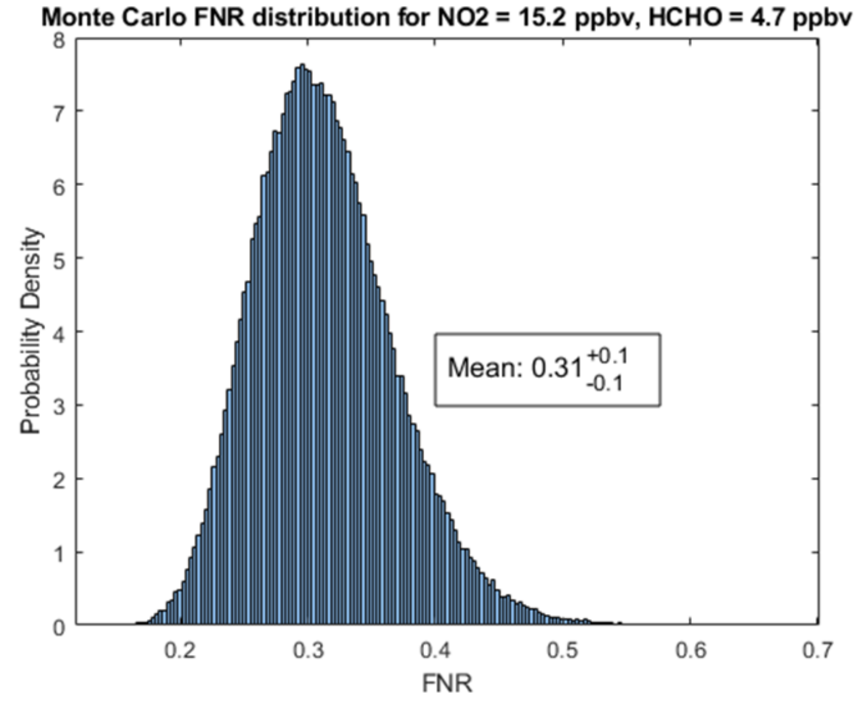

Fig. S1. Probability distribution functions of f^*_{NO} and HCHO to NO_2 (FNR) for a typical Los Angeles plume transect during AEROMMA 2023 determined Monte Carlo method. The mean of the distribution is reported with 95% confidence intervals. Here S is ∑[ISOPOOH+IEPOX].

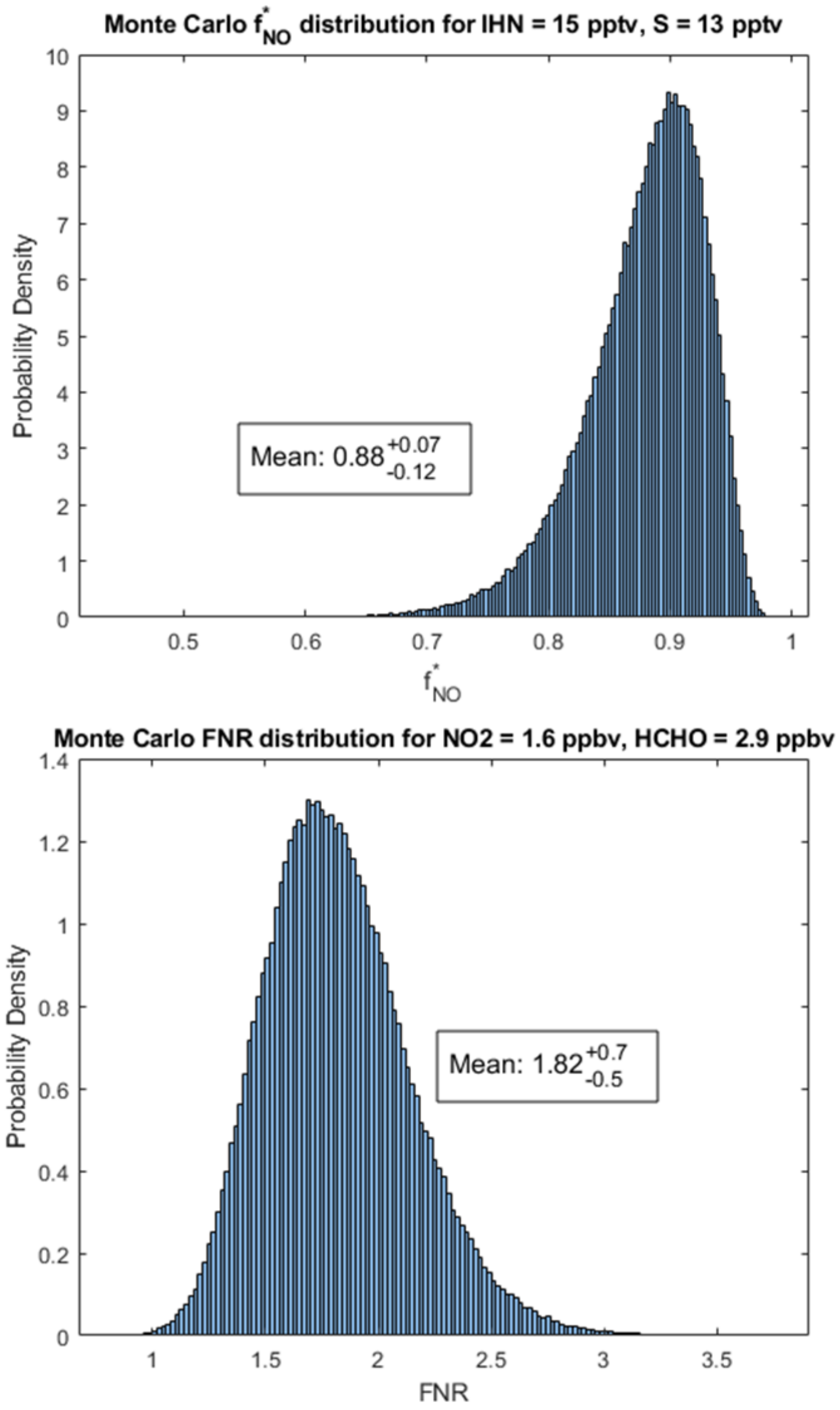

Fig. S2. Probability distribution functions of f^*_{NO} and HCHO to NO_2 (FNR) for a typical Chicago plume transect during AEROMMA 2023 determined Monte Carlo method. The mean of the distribution is reported with 95% confidence intervals. Here S is ∑[ISOPOOH+IEPOX].

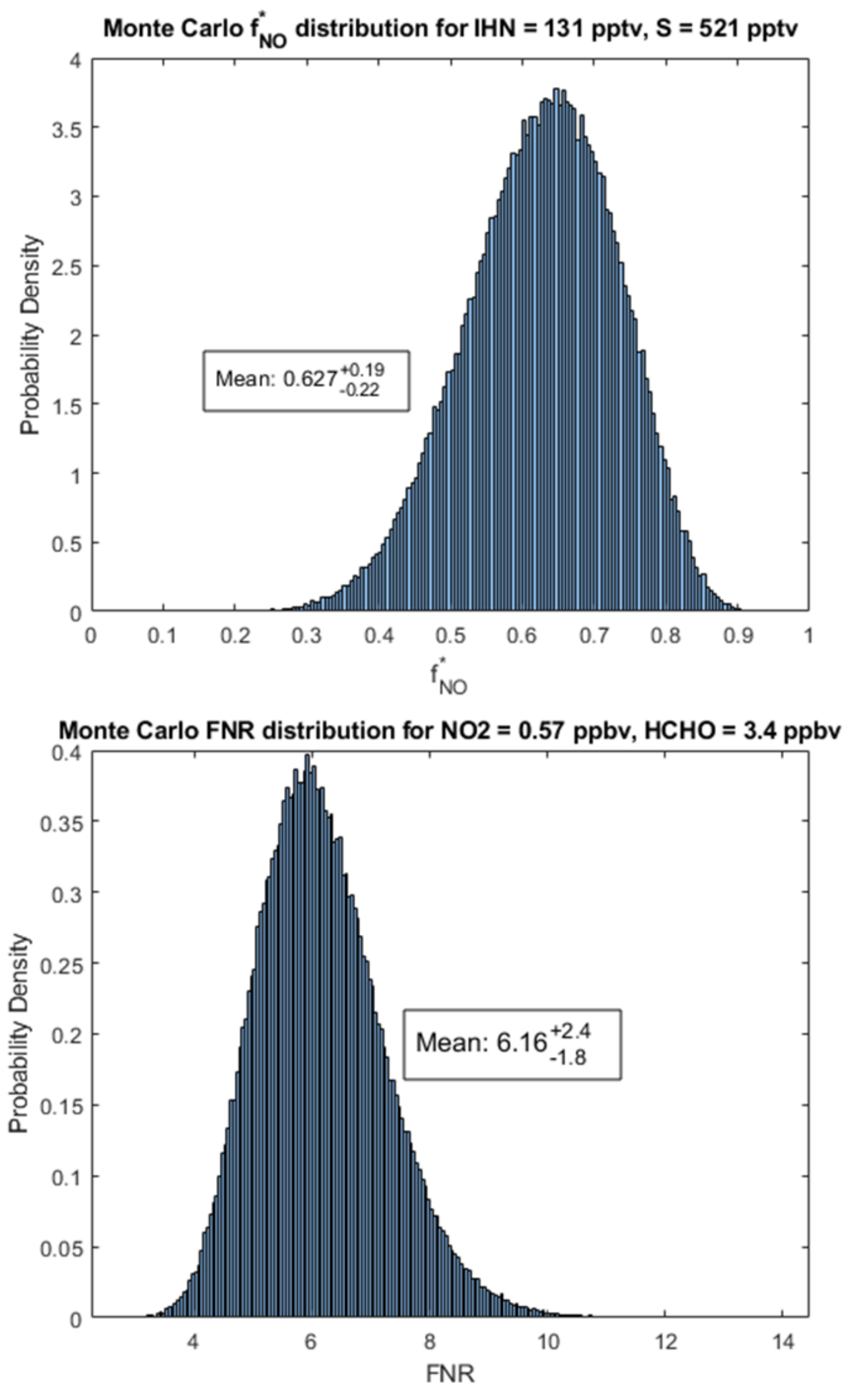

Fig. S3. Probability distribution functions of f^*_{NO} and HCHO to NO_2 (FNR) for a typical New York City plume transect during AEROMMA 2023 determined Monte Carlo method. The mean of the distribution is reported with 95% confidence intervals. Here S is ∑[ISOPOOH+IEPOX].

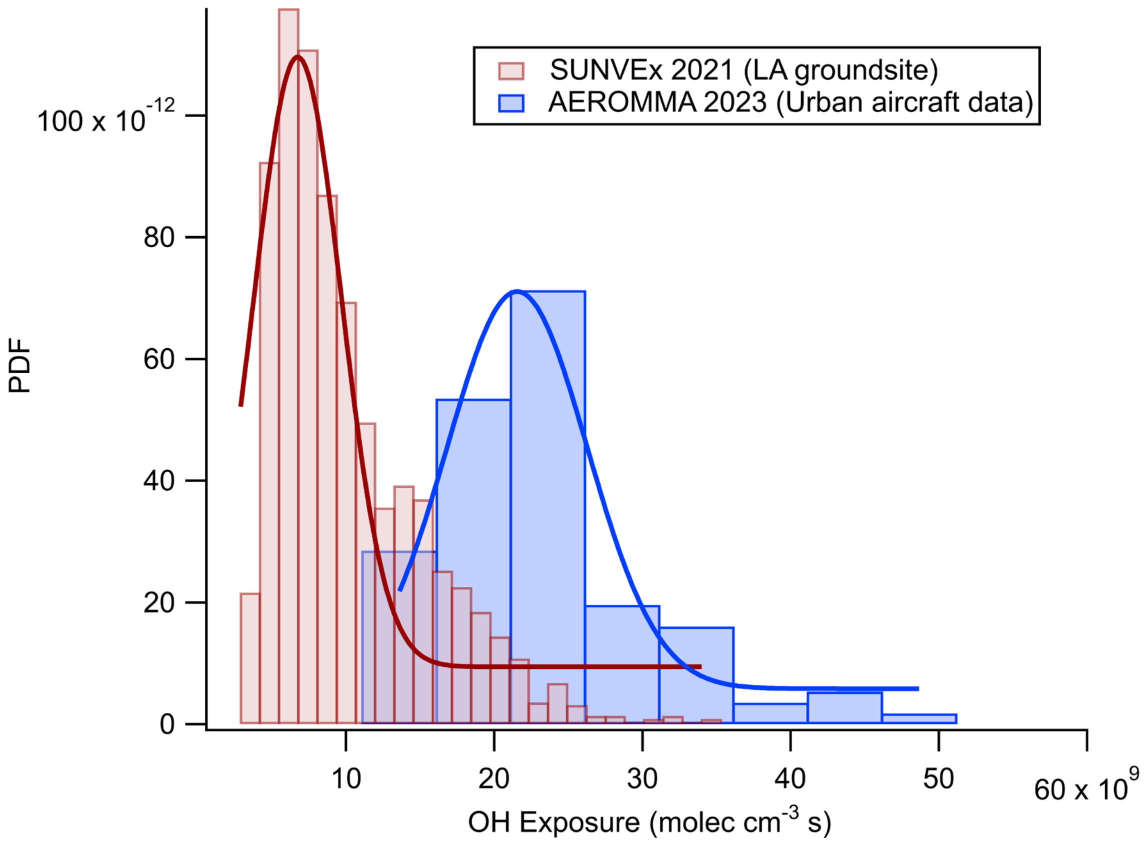

Fig. S4. Probability distribution function of OH exposures measured during SUNVEx 2021 and AEROMMA 2023 determined by the MVK and MACR sequential models and averaged.

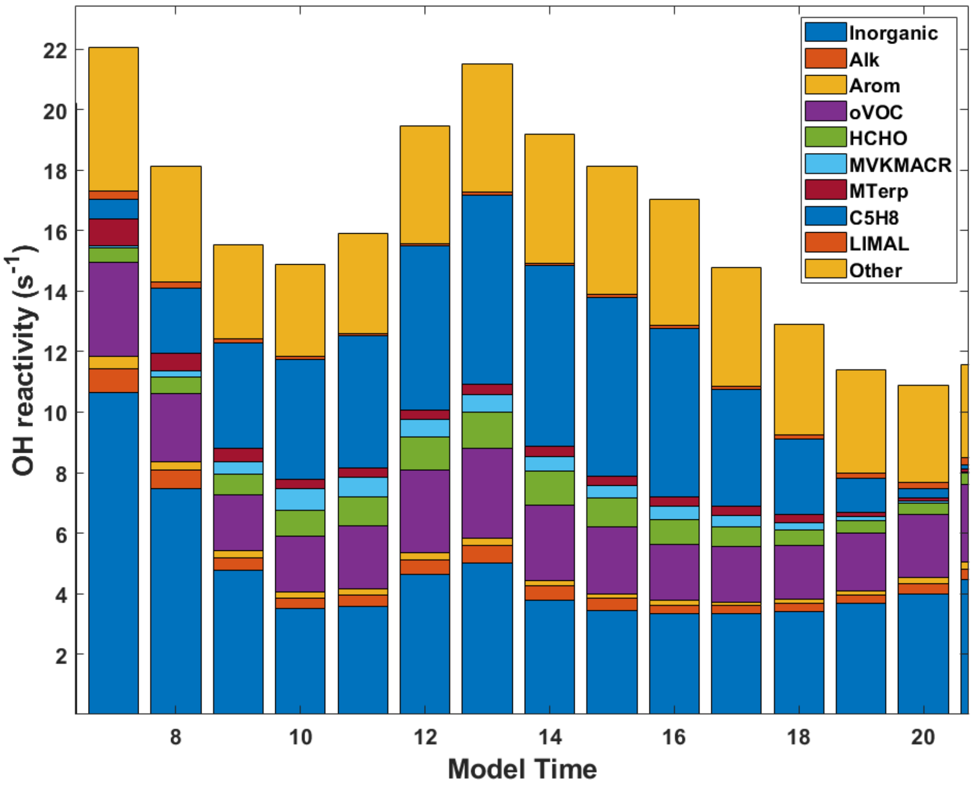

Fig. S5. OH reactivity determined at the Pasadena ground site during the summer of 2021 (SUNVEx field campaign). Inorganic: CO, H_2, O_3, HO_2, H_2O_2, NO_2, NO; Alk: C_2H_4, C_2H_6, C_3H_8, i-C_4H_{10}, i-C_5H_{12}, n-C_5H_{12}, n-C_6H_{14}, n-$C_{10}H_{22}$, CH_4; Arom: benzene, toluene, ethylbenzene, 1,2,4-trimethyl-benzene, 1,3,5-trimethyl-benzene, benzaldehyde; oVOC: acetaldehyde, propionaldehyde, butyraldehyde, glycolaldehyde, glyoxal, methanol, ethanol, hydroxyacetone, 2,3-butanedione, acrolein; MVKMACR: methyl vinyl ketone, methacrolein; MTerp: α-pinene, β-pinene, limonene, pinonaldehyde; LIMAL: limononaldehyde.

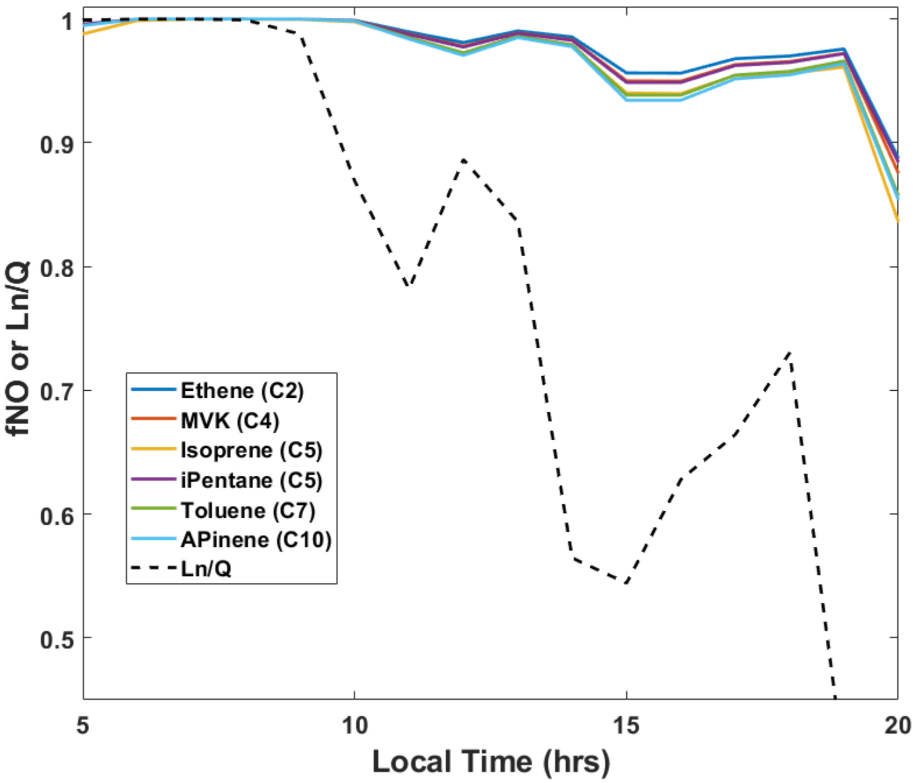

Fig. S6. Ground site box model fraction of peroxy radical reacting with NO and L_n/Q.

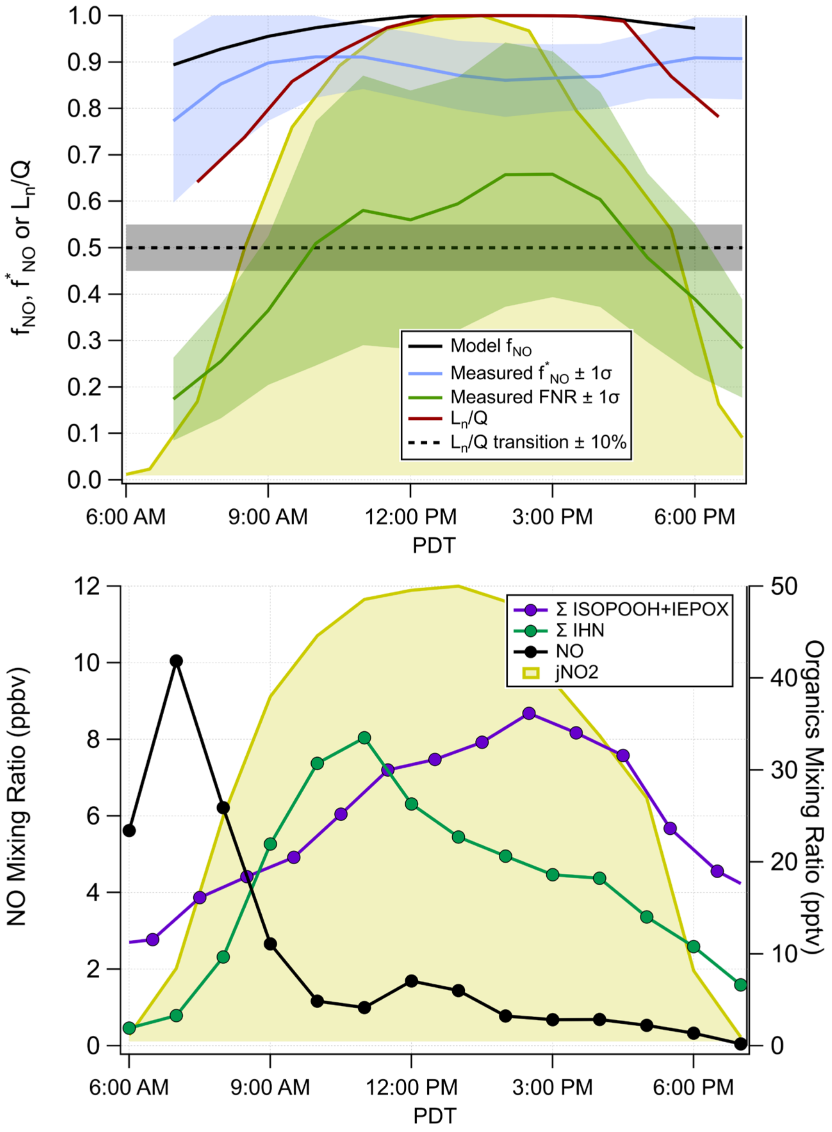

Fig. S7. Ground site observations and box model results for the isoprene system from Pasadena, CA during a five-day O_3 event which occurred in the SUNVEx campaign in 2021. Top panel shows five-day average chemical regime proxies, both measured (f^*_{NO} and FNR) and modelled (L_n/Q and f_{NO}). The bottom panel shows average diurnal mixing ratios of NO, ∑IHN isomers and ∑ISOPOOH + IEPOX isomers.

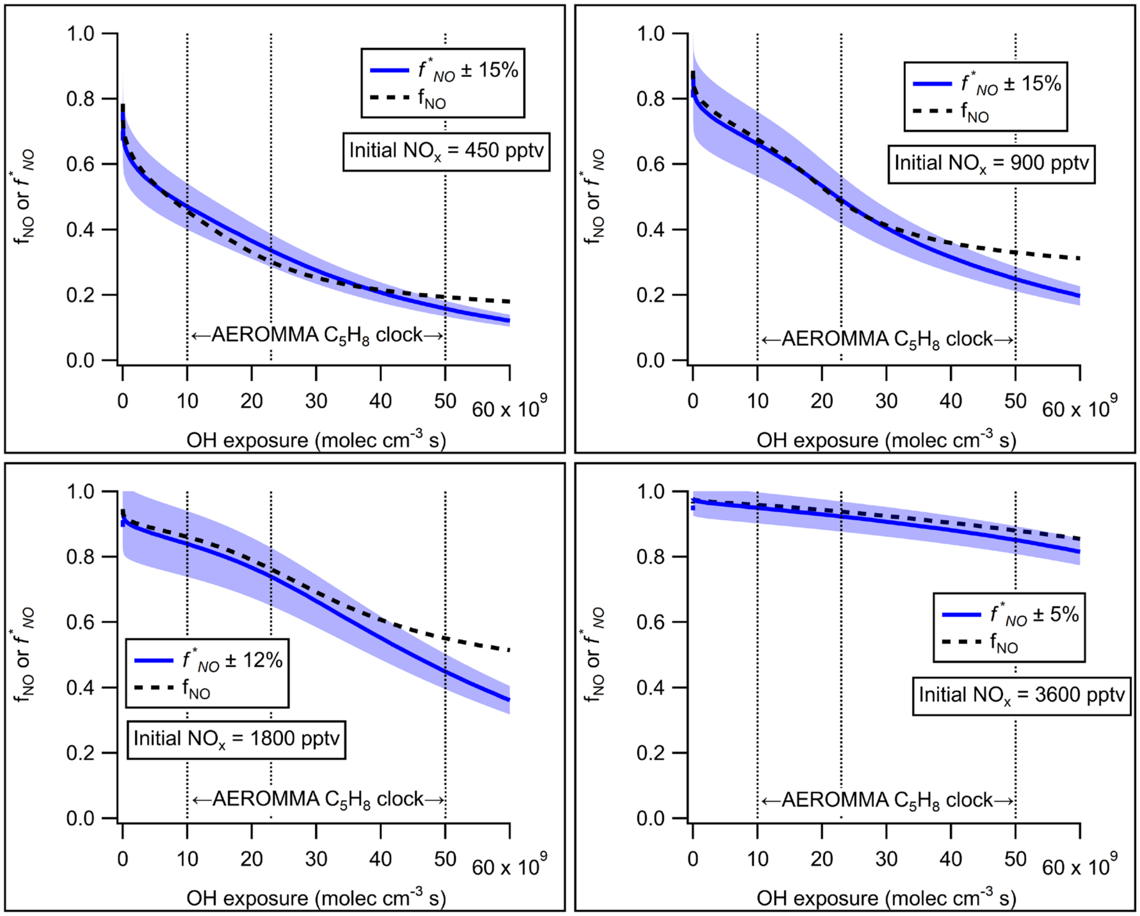

Fig. S8. Comparison of f^*_{NO} and f_{NO} in an isoprene box model over the range of OH exposures observed during AEROMMA for four initial NO_x mixing ratios (3.6, 1.8, 0.9 and 0.45 ppbv).

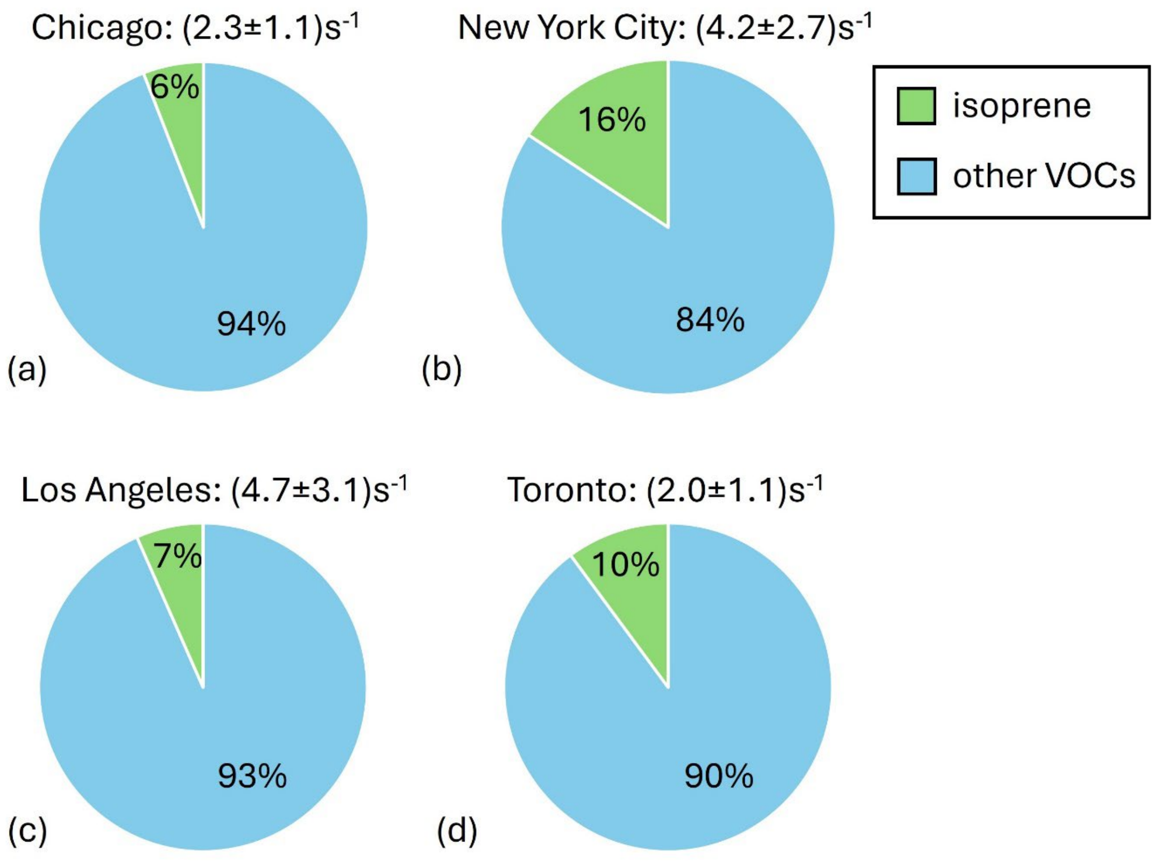

Fig. S9. Average OH reactivity from isoprene and other VOCs in urban plumes of the metropolitan areas measured during flights of the AEROMMA campaign.

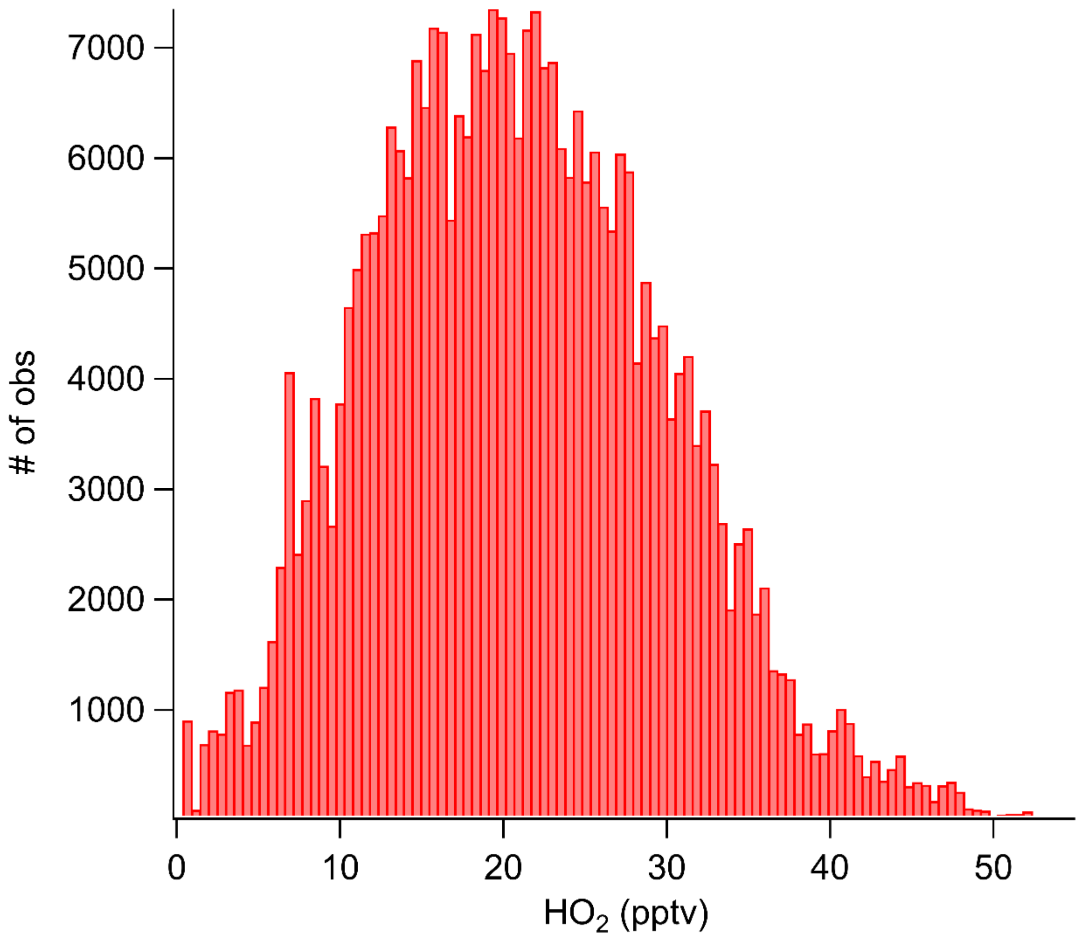

Fig. S10. Histogram of WRF-Chem retrieved HO_2 during AEROMMA 2023.

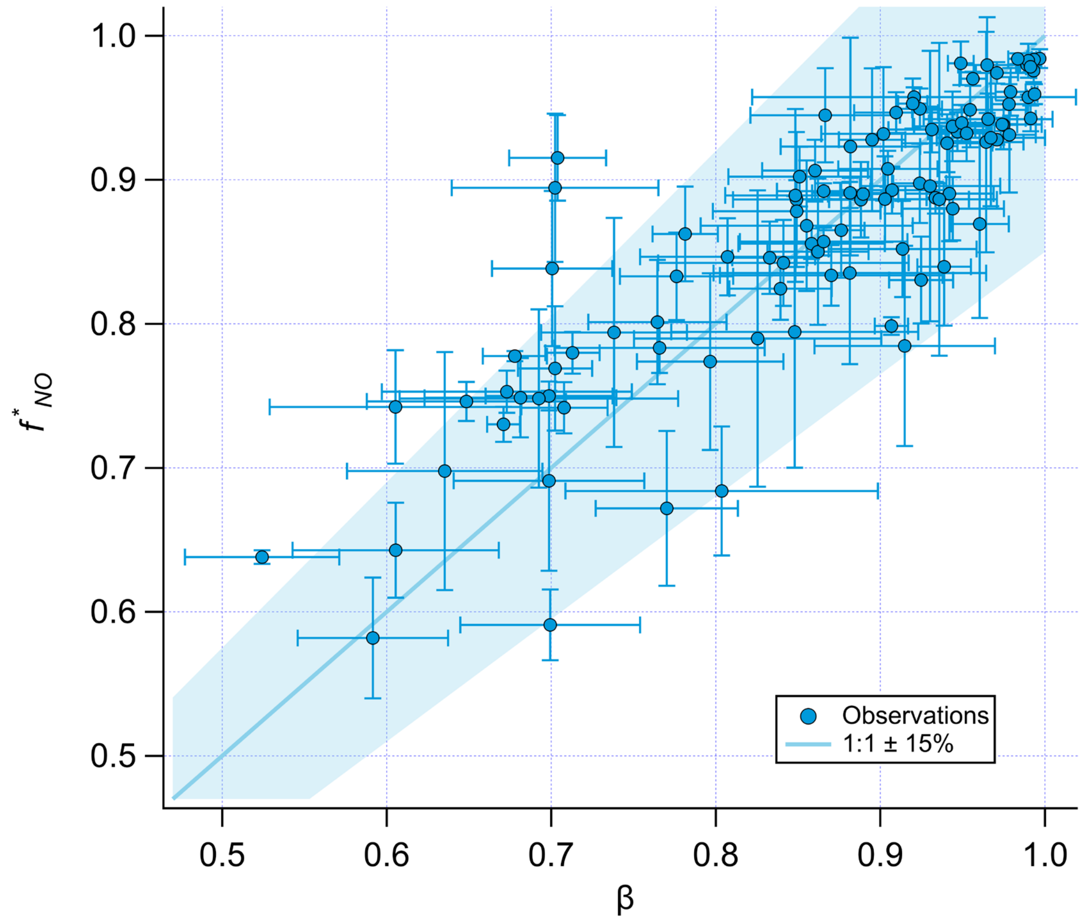

Fig. S11. AEROMMA transect correlation of f^*_{NO} and β.

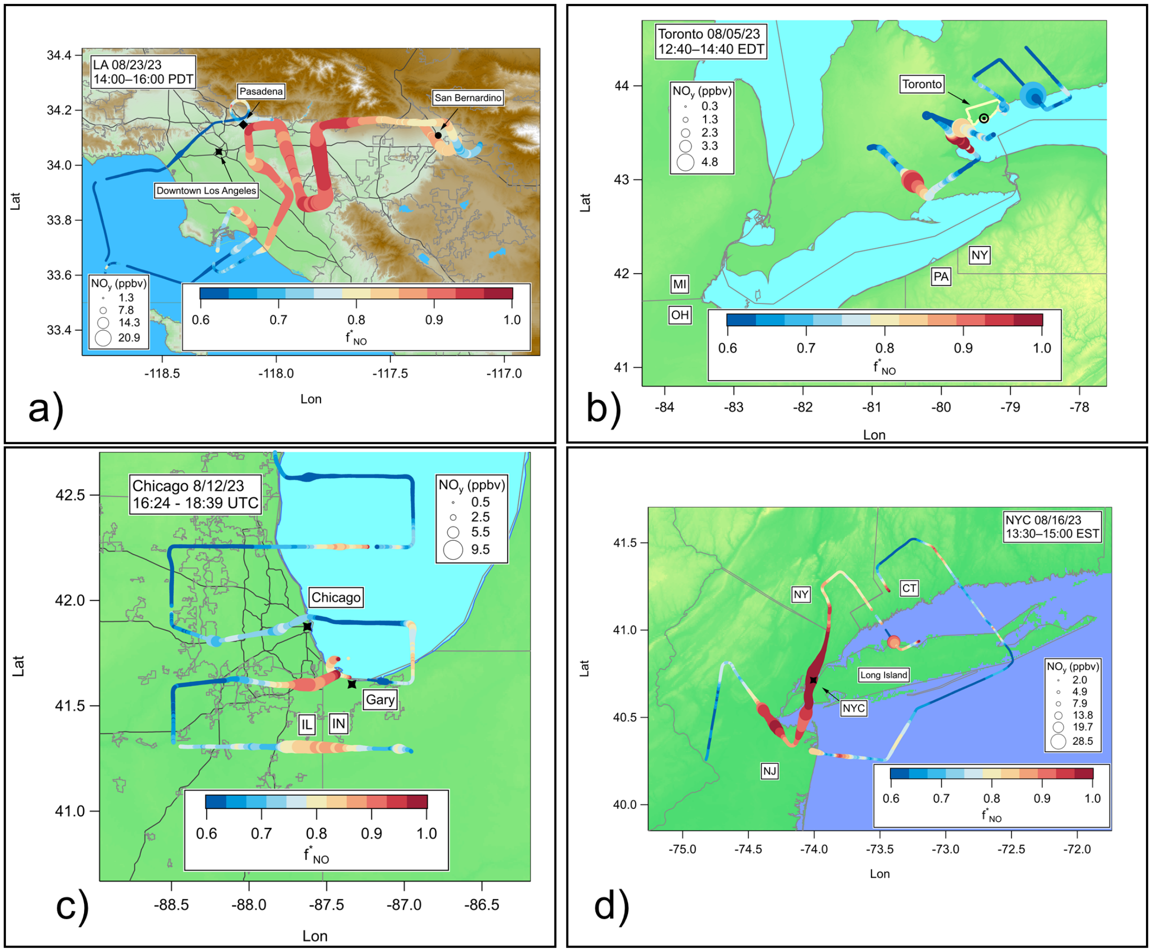

Fig. S12. Example flight tracks from the NASA DC-8 during AEROMMA for each sampled mega city: a) Los Angeles, b) Toronto, c) Chicago, and d) New York City. Flight track colored by f^*_{NO} and sized by the mixing ratios of NO_y, which is a proxy for NO_x emissions in these regions.

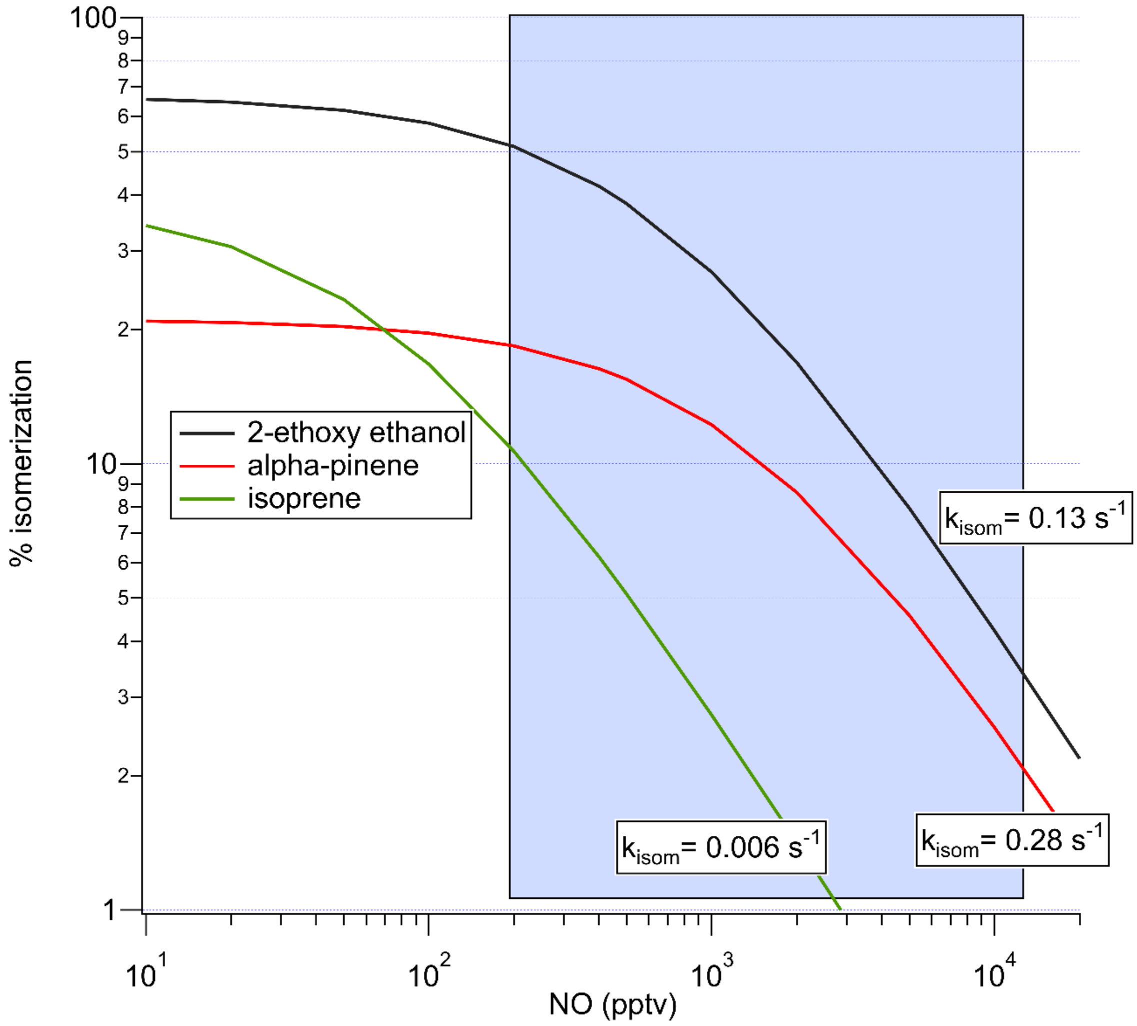

Fig. S13. Calculated fraction of isomerization (f_{isom}) for three VOC RO_2 at average AEROMMA HO_2 conditions (24.5 pptv) over the range of NO mixing ratios observed (blue shading).

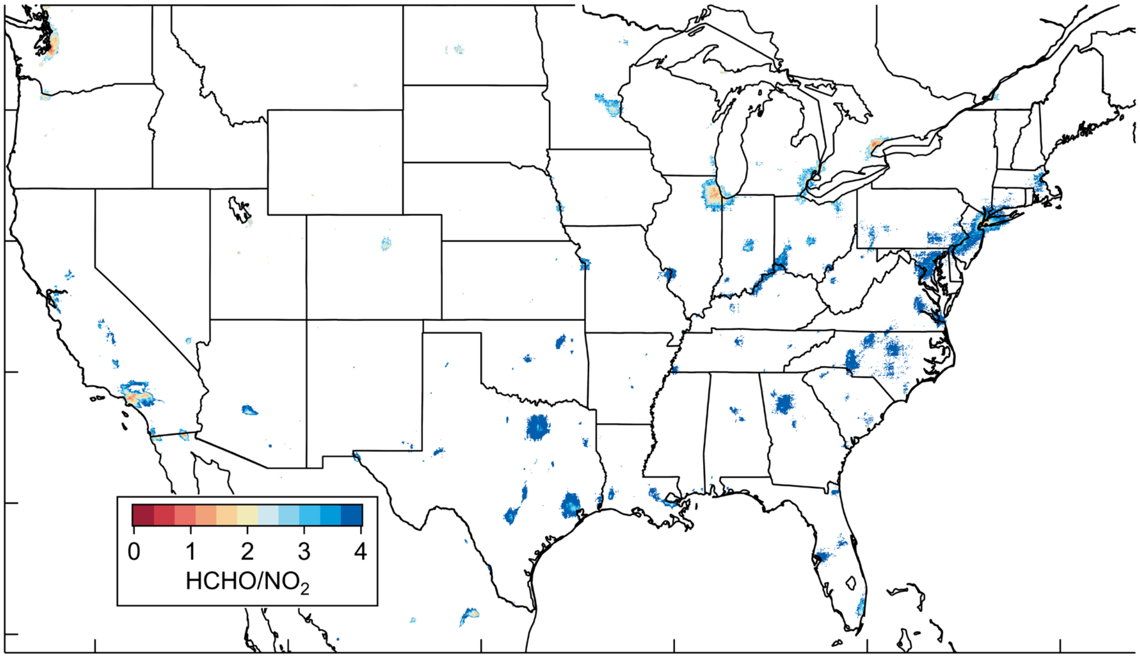

Fig. S14. TEMPO satellite retrieval for 1 PM – 5 PM PDT average for the month of August 2023 of $HCHO/NO_2$. Data are filtered based on the published thresholds for HCHO and NO_2 (see Materials and Methods).

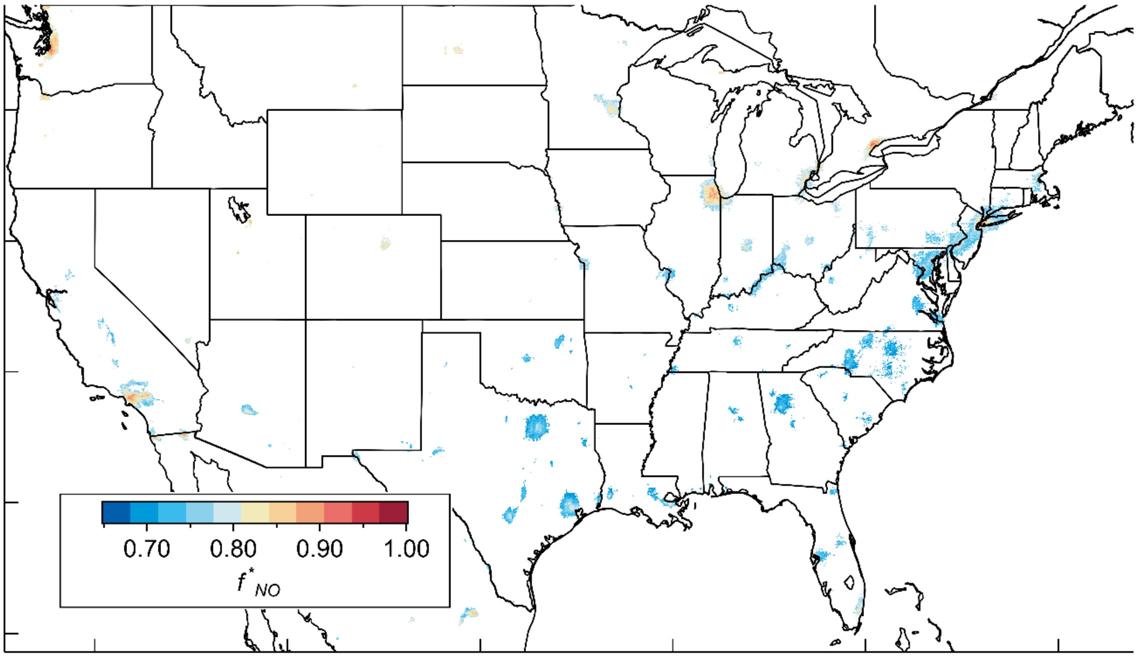

Fig. S15. TEMPO satellite retrieval for 1 PM – 5 PM PDT average for the month of August 2023 of f^*_{NO} as estimated with the relationship to FNR determined during AEROMMA (see SI Figure 16). Data are filtered based on the published thresholds for HCHO and NO_2 (see Materials and Methods).

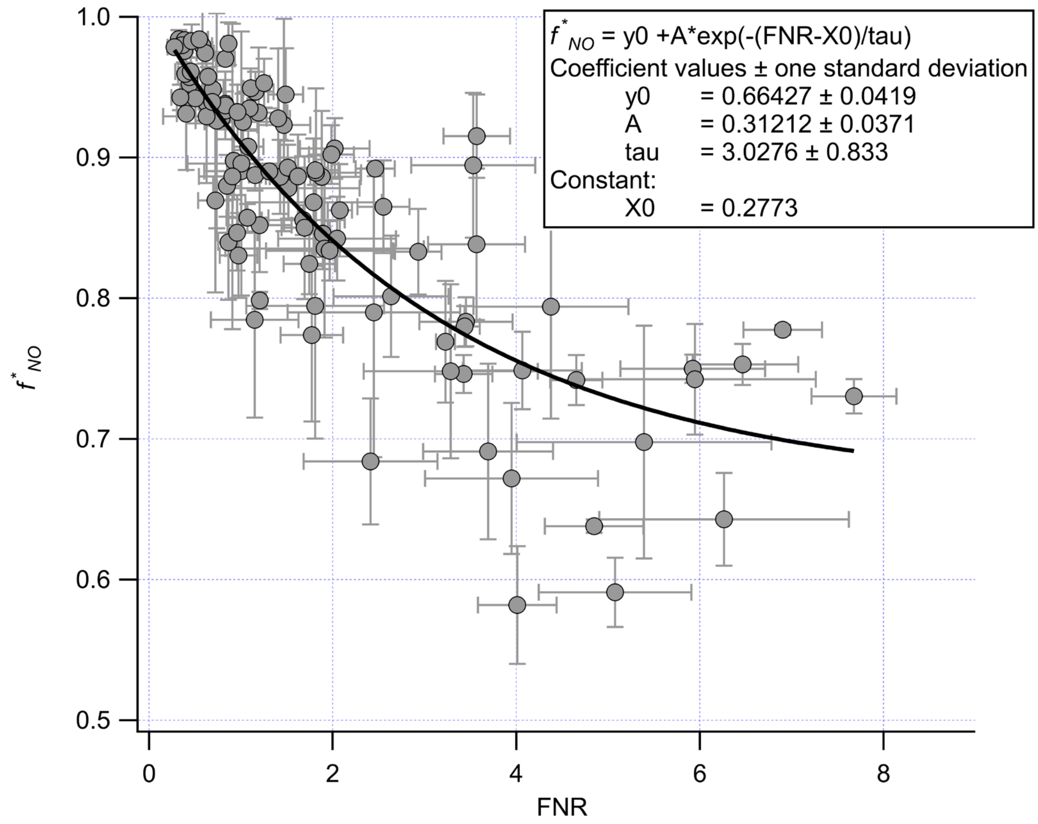

Fig. S16. f^*_{NO} vs. FNR fit from AEROMMA transect data

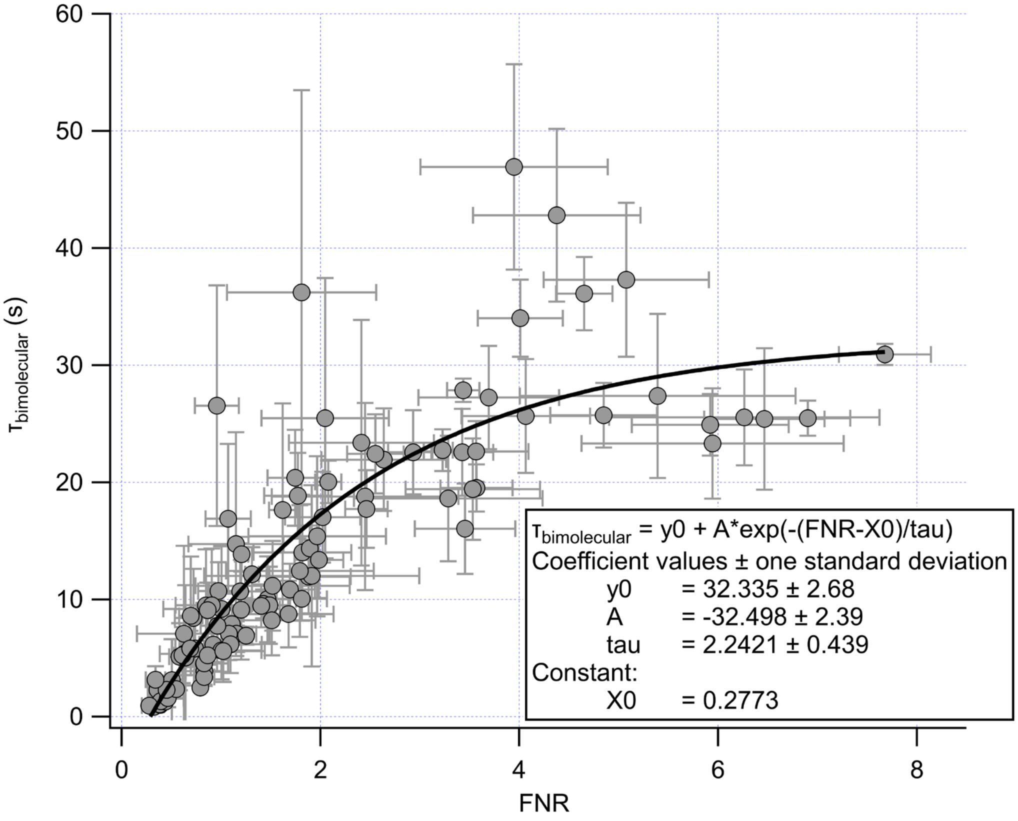

Fig. S17. RO_2 bimolecular lifetime vs. FNR fit from AEROMMA transect data.

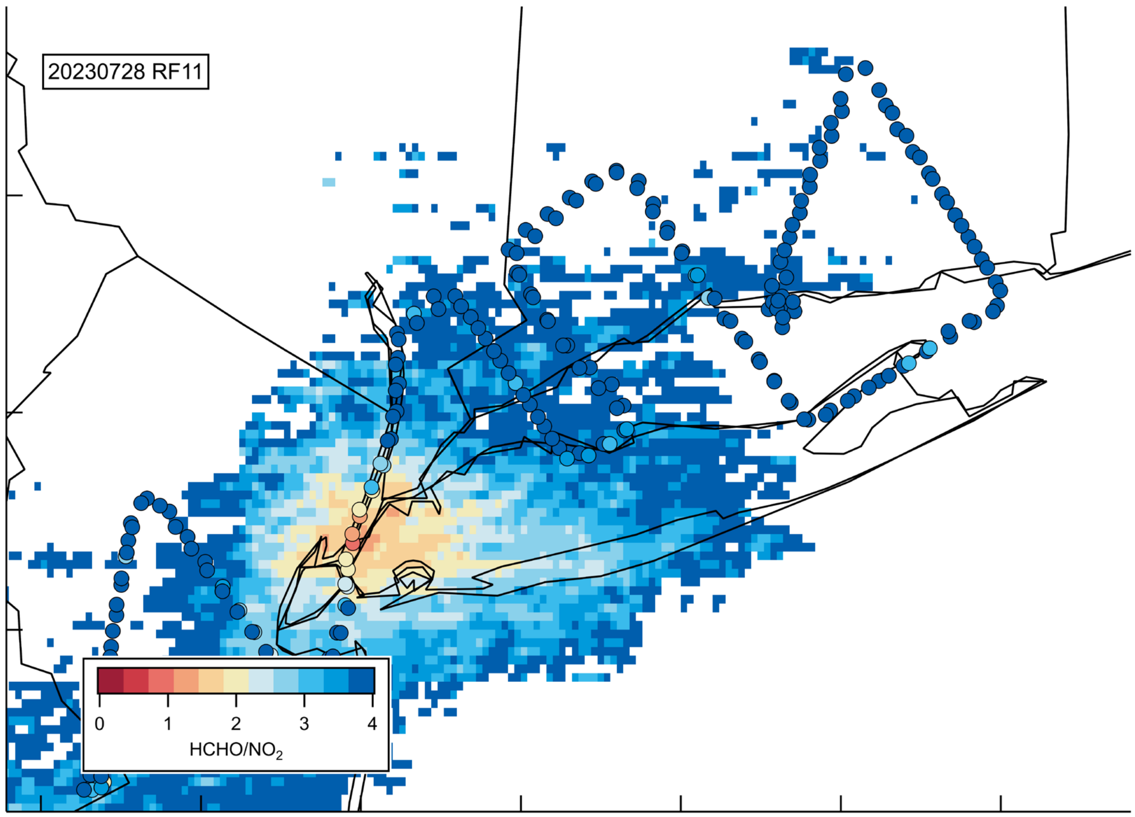

Fig. S18. August 2023 average TEMPO FNR (1300 to 1700 PDT) and DC-8 in-situ boundary layer FNR for 20230728 research flight.

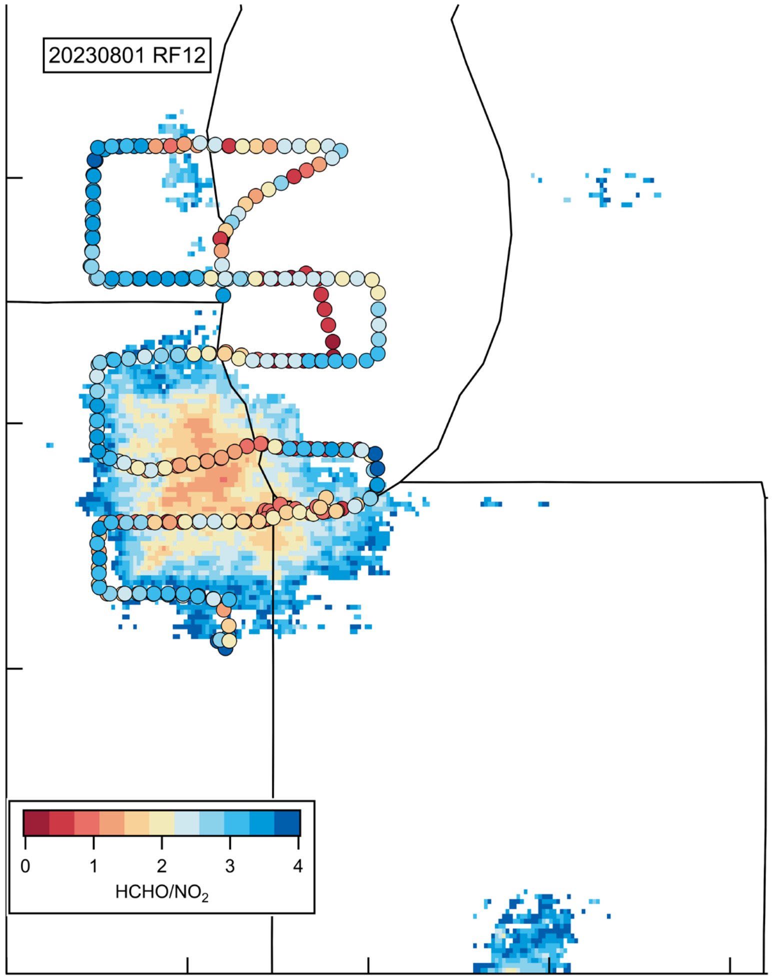

Fig. S19. August 2023 average TEMPO FNR (1300 to 1700 PDT) and DC-8 in-situ boundary layer FNR for 20230801 research flight.

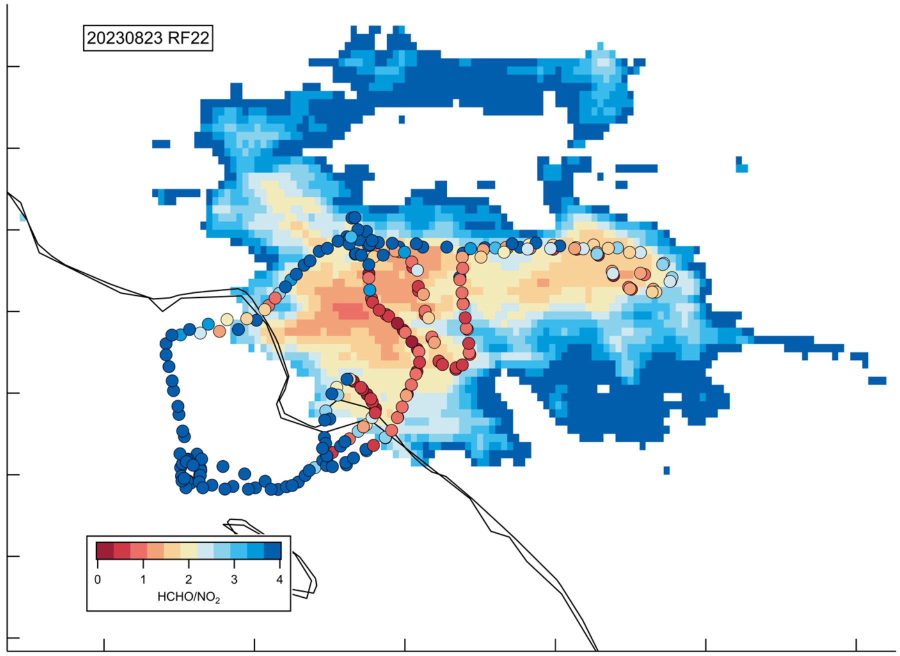

Fig. S20. August 2023 average TEMPO FNR (1300 to 1700 PDT) and DC-8 in-situ boundary layer FNR for 20230823 research flight.

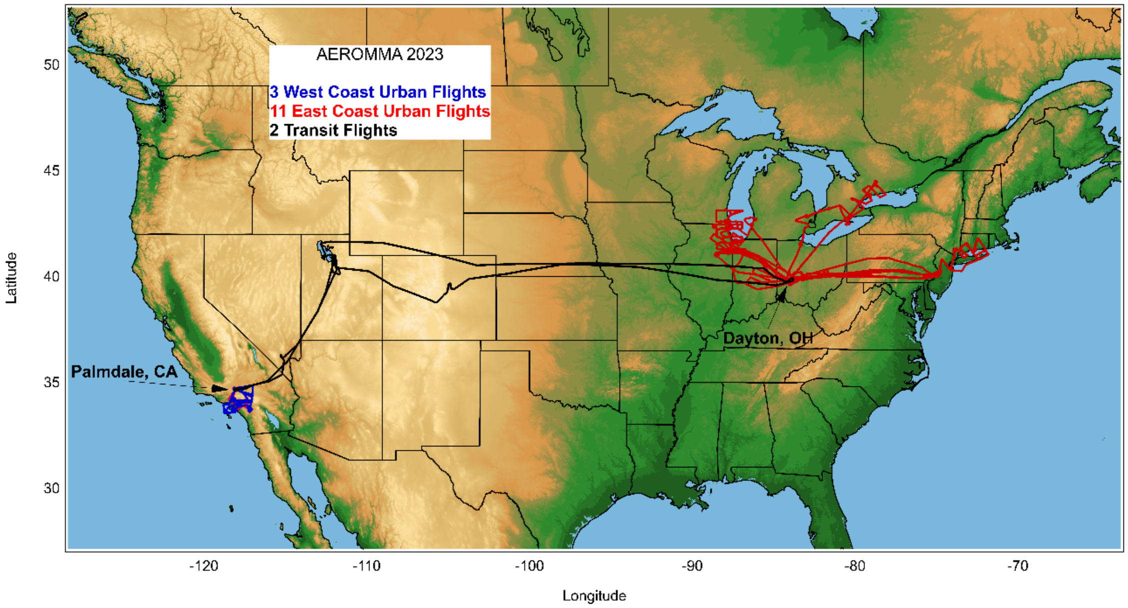

Fig. S21. AEROMMA Urban campaign flight tracks.

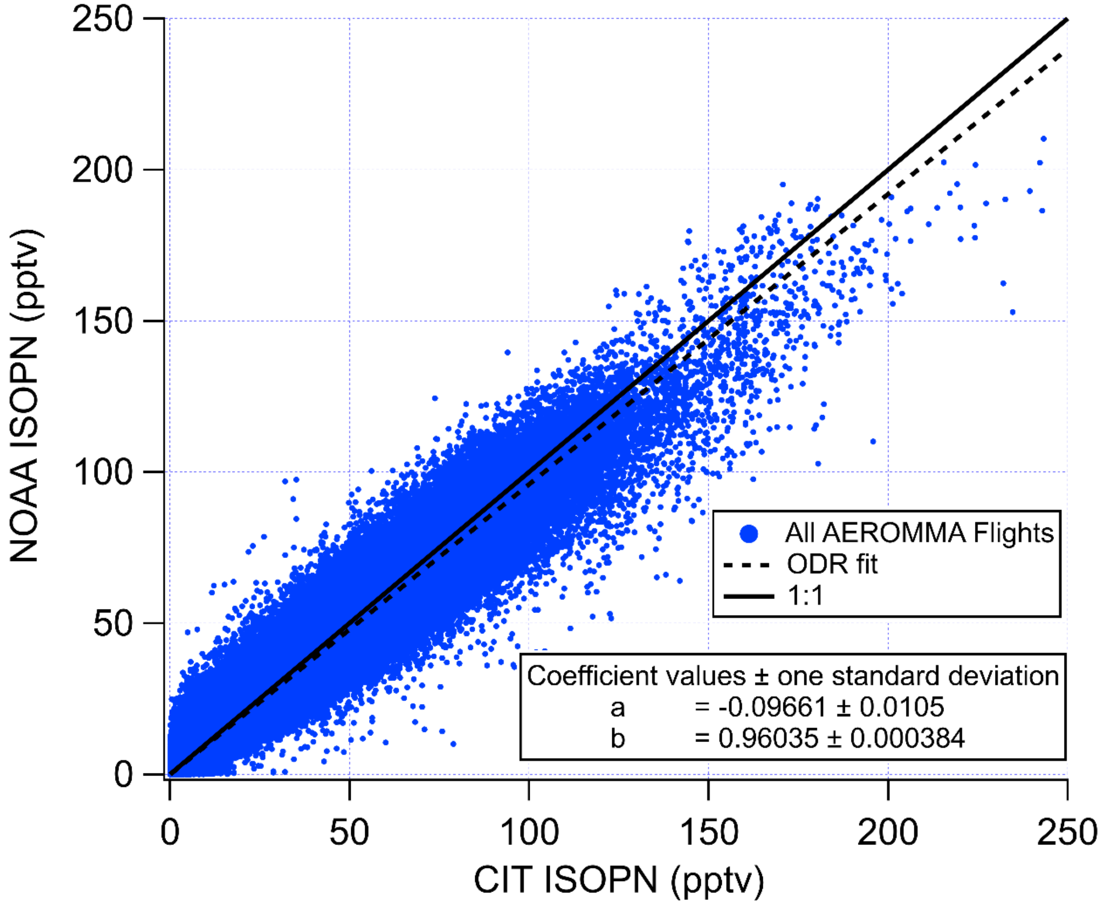

Fig. S22. Isoprene hydroxy nitrate measurement comparison between NOAA I^- CIMS and CIT CF_3O^- CIMS from AEROMMA campaign.

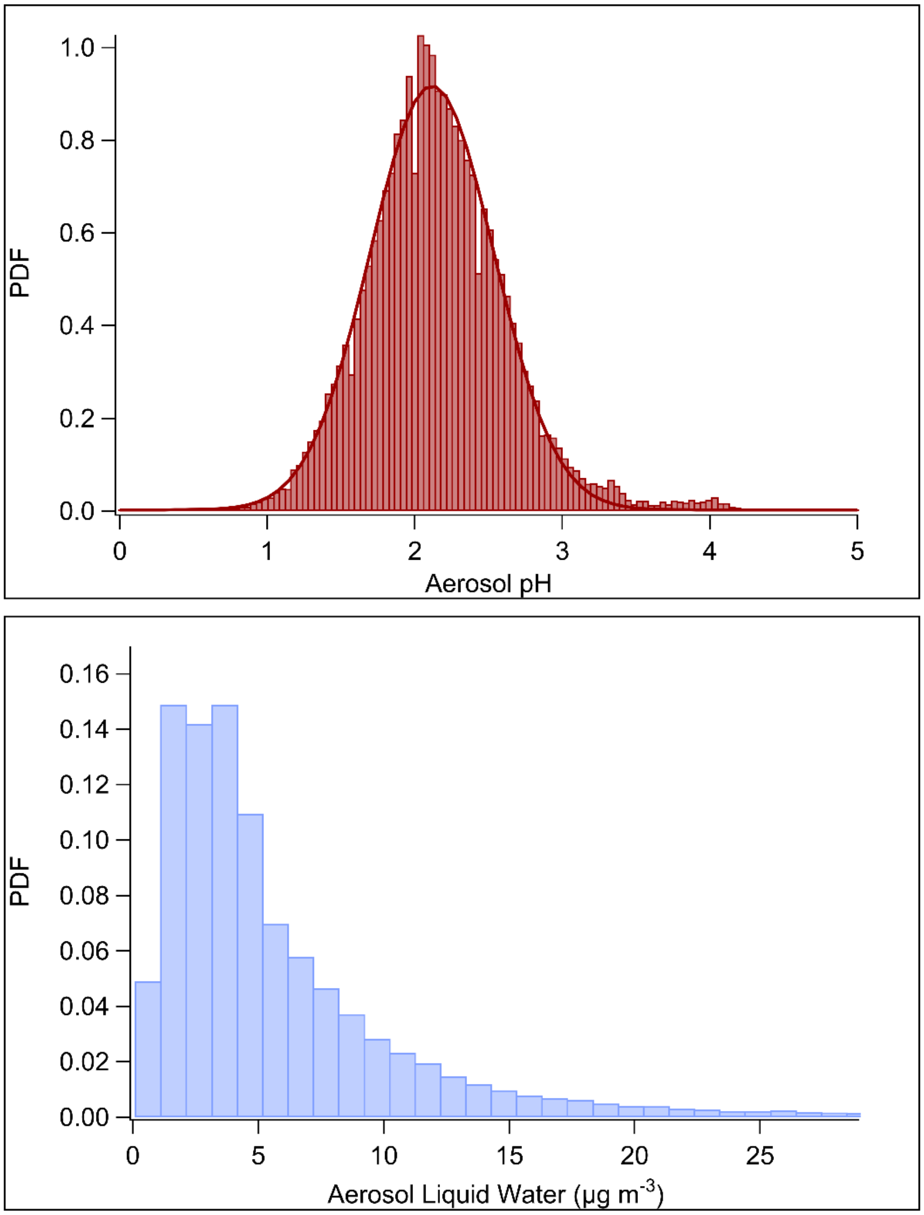

Fig. S23. Probability distribution functions of aerosol pH and aerosol liquid water during AEROMMA 2023 determined by ISORROPIA-lite (see SI Section 1.3 for details).

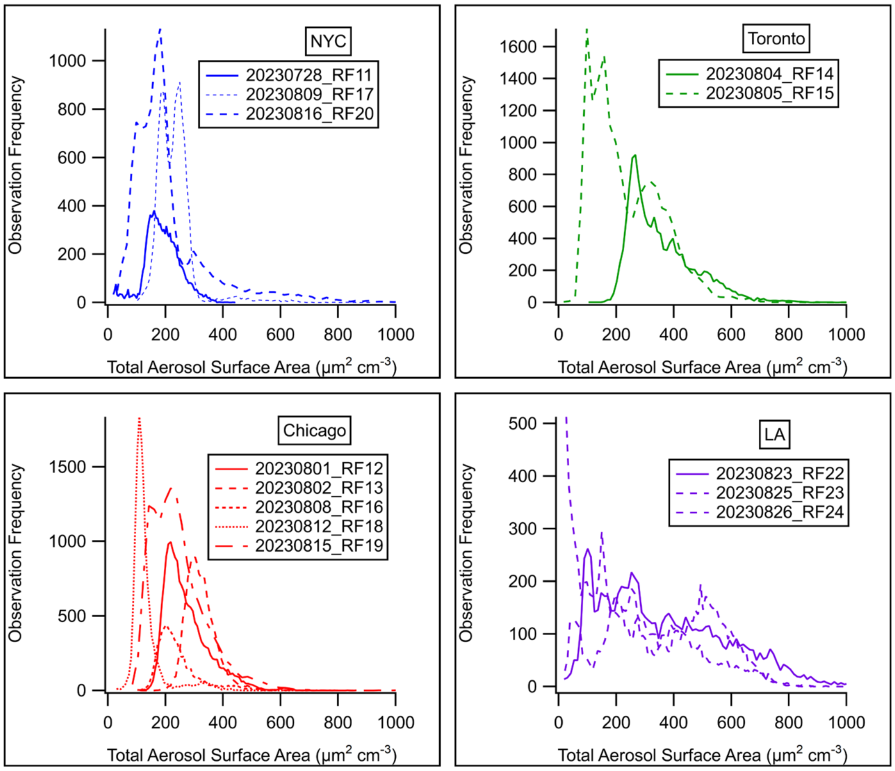

Fig. S24. Calculated total aerosol surface area measurements at ambient relative humidity in the boundary layer for all urban research flights from the AEROMMA campaign.

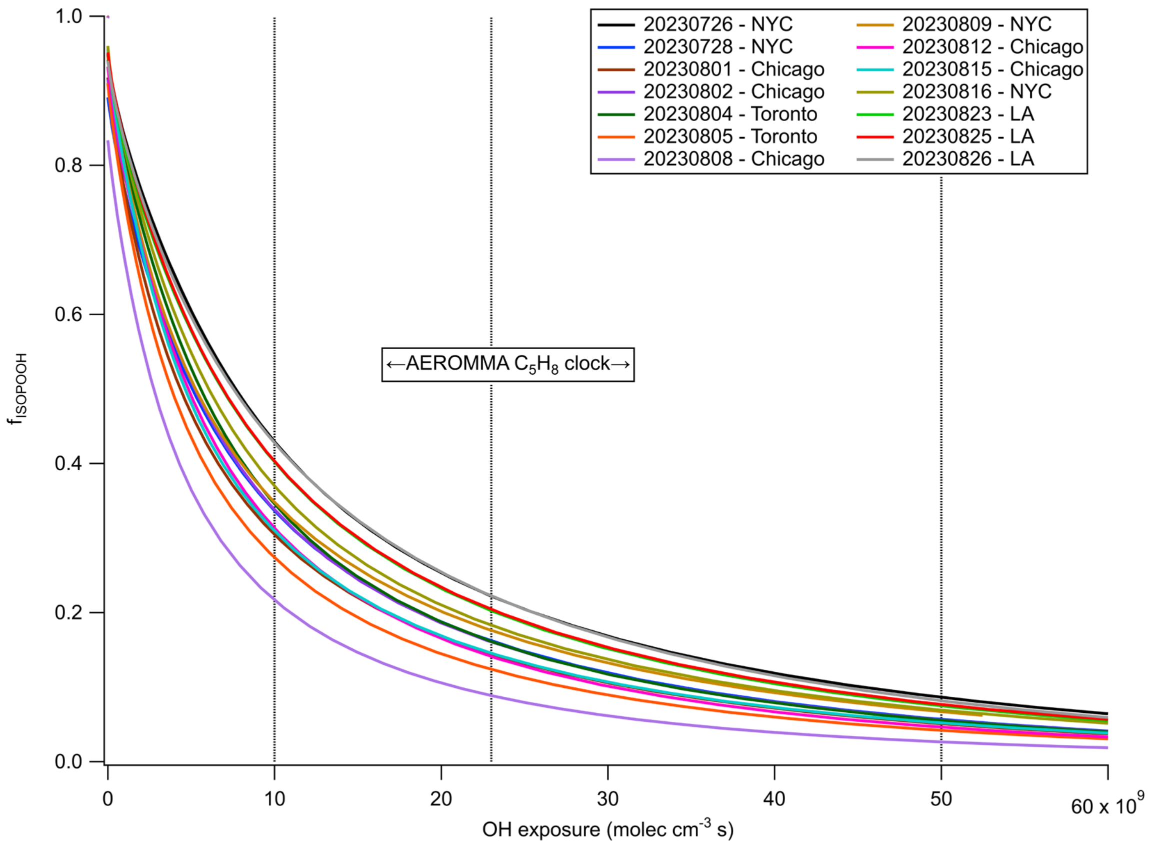

Fig. S25. Plot of [ISOPOOH]/∑[ISOPOOH + IEPOX] (fraction of ISOPOOH or $f_{ISOPOOH}$) as a function of OH exposure for each research flight box model developed for urban flights during AEROMMA 2023. The range of OH exposures measured during AEROMMA are indicated by vertical dashed lines with the center line indicating the average OH exposure (2.3 x 10^{10} molec cm^{-3} s).

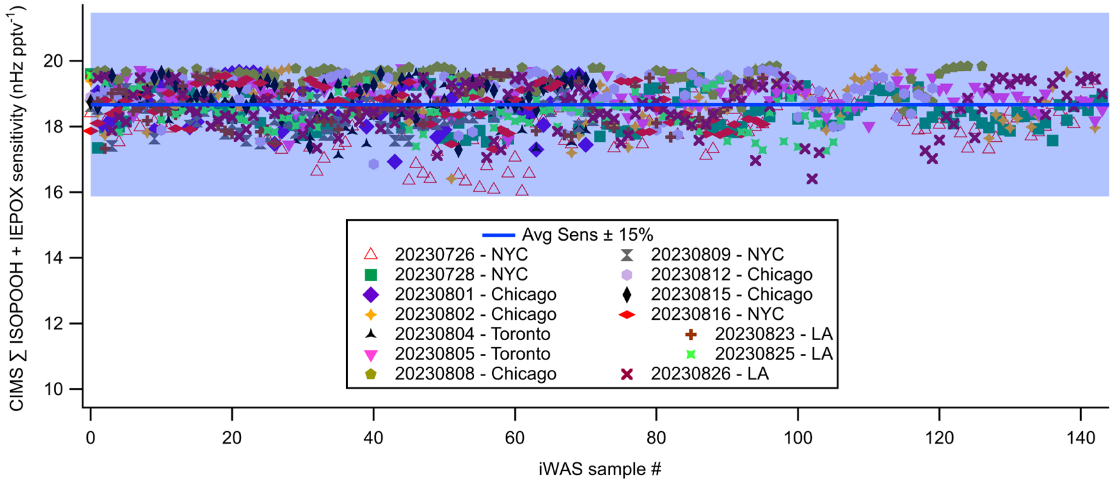

Fig. S26. AEROMMA 2023 Iodide CIMS ∑[ISOPOOH + IEPOX] sensitivity for a cluster ratio = 0.55 for each iWAS sample determined OH exposure using MVK and MACR sequential model. Campaign average sensitivity ± 15% is shown in blue solid line ± shading.

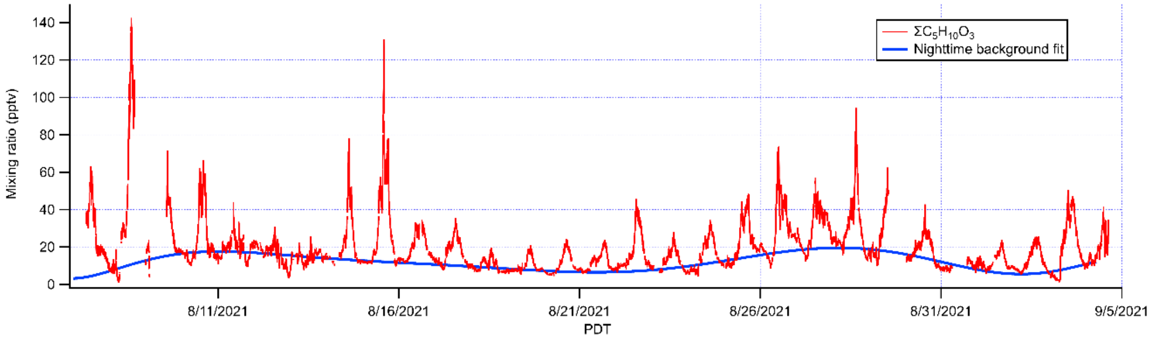

Fig. S27. SUNVEX 2021 Iodide CIMS ∑[ISOPOOH + IEPOX] measured mixing ratio and fitted nighttime background.

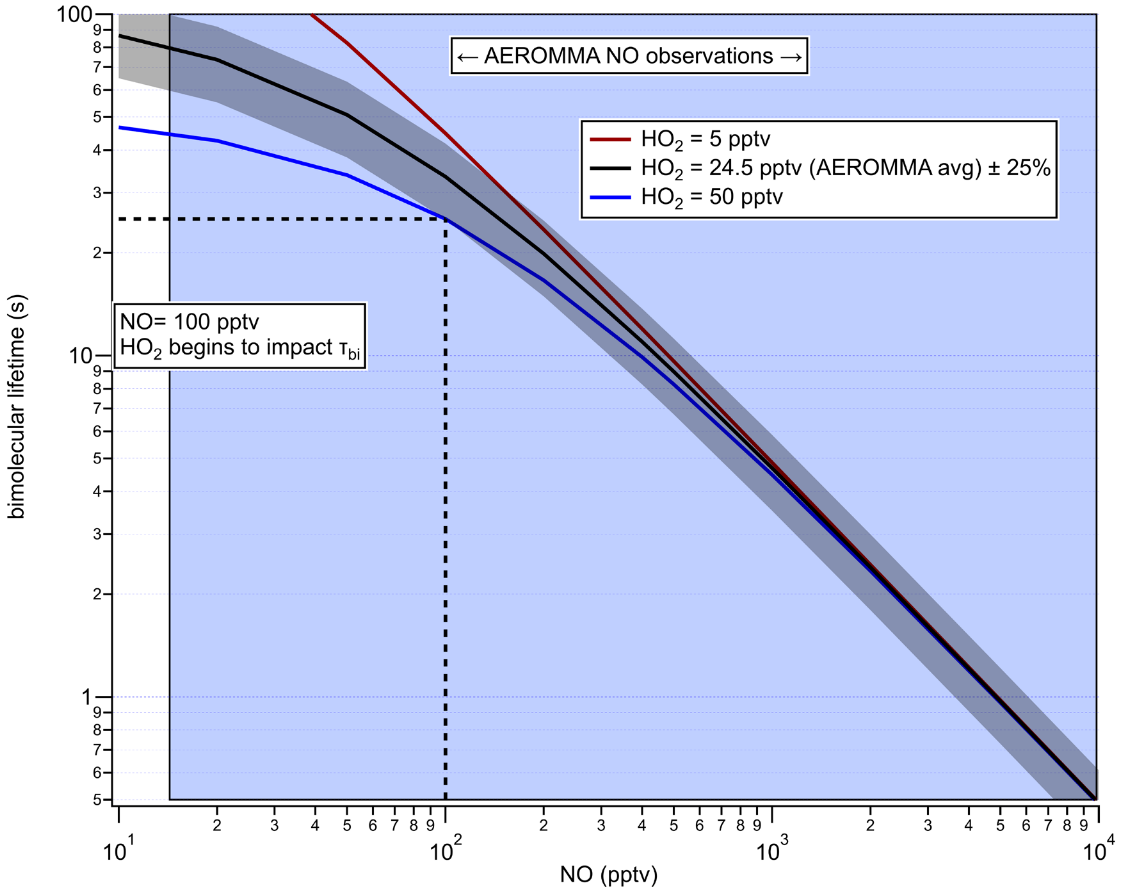

Fig. S28. Calculated RO_2 bimolecular lifetime (τ_{bi}) for three HO_2 mixing ratios (5, 24.5 and 50 pptv) over the range of observed NO mixing ratios for urban transects during AEROMMA. Both low and high HO_2 cases diverge from AEROMMA average HO_2 (24.5) at approximately 100 pptv of NO.

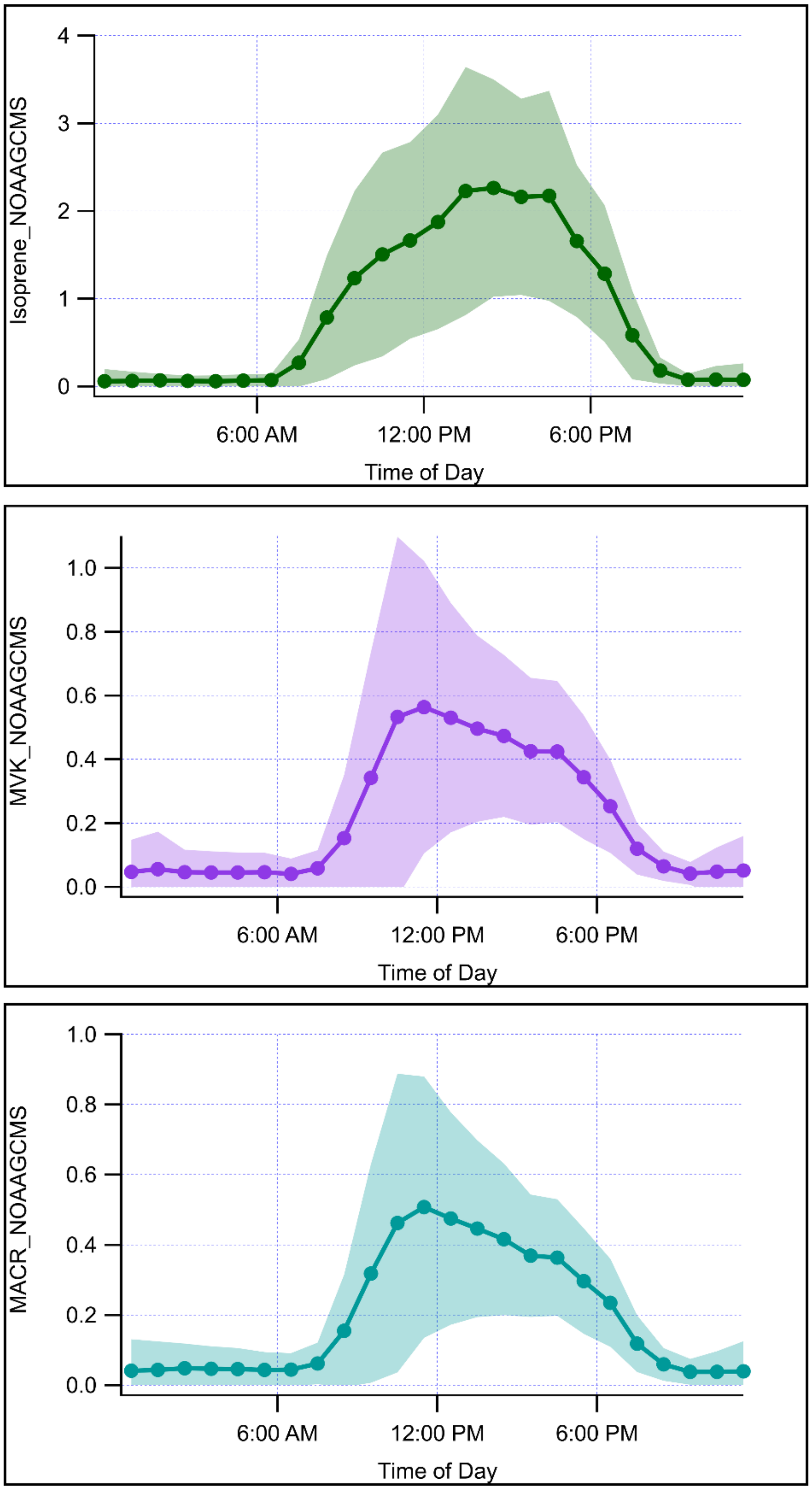

Fig. S29. Ground site observations from Pasadena, CA during the SUNVEx campaign in 2021. Average diurnal mixing ratios of Isoprene, MVK and MACR, with standard deviation shading.

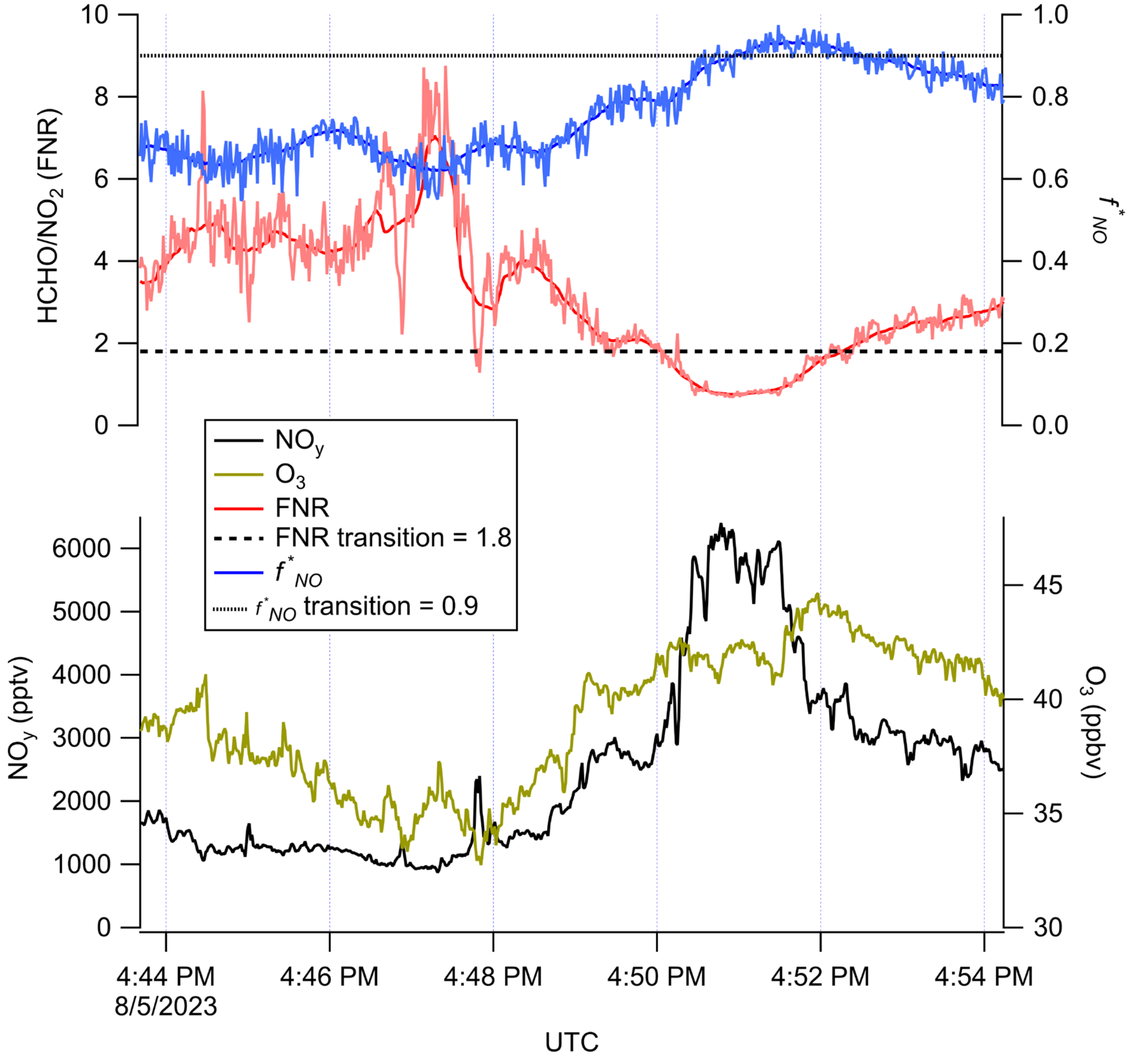

Fig. S30. Example plume transect from Toronto on 8/5/23 for both $HCHO/NO_2$ and f^*_{NO} (top panel), bottom panel shows NOy and O_3 for the same transect. Solid lines represent 30 second box car smoothing of 1 Hz data. Dashed lines in the top panel represent the defined transitions for both $HCHO/NO_2$ and f^*_{NO}.

Flight Date	City	ALW (µg m^{-3})	pH	Aerosol SA (µm^2 cm^{-3})	Particle Radius (nm)	Temp. (degK)	RH (%)	Press. (mbar)	jNO_2 x 10^3 (s^{-1})	HO_2 (pptv)	NO (ppbv)	NO_2 (ppbv)	C_5H_8 (ppbv)	τ_{IHN} (hrs)[a]	$\tau_{ISOP+IEPOX}$ (hrs)[b]
20230726	NYC	6.5[c]	1.9[c]	287 ± 96[c]	137 ± 21[c]	295	71	945	8.6	31.6 ± 3.1	2.0	6.00	4.00	2.2	9.9
20230728	NYC	5.1	2.0	226 ± 53	114 ± 17	297	67	948	8.2	40.2 ± 4.7	2.0	6.00	2.25	2.2	10.2
20230801	Chi	5.6	2.3	367 ± 80	118 ± 11	296	60	965	6.5	23.4 ± 4.0	2.0	7.95	1.50	2.1	10.0
20230802	Chi	5.9	2.2	351 ± 39	120 ± 3.0	296	58	963	8.1	28.8 ± 3.2	2.0	6.38	2.00	2.1	9.9
20230804	Tor	10.8	2.5	382 ± 31	168 ± 11	294	68	957	8.6	19.4 ± 6.7	2.0	6.00	2.00	1.7	9.9
20230805	Tor	6.0	2.4	340 ± 92	145 ± 13	292	66	960	7.1	22.1 ± 3.6	2.0	7.27	1.00	2.1	10.0
20230808	Chi	8.4	2.6	352 ± 75	143 ± 25	294	62	946	9.2	32.3 ± 1.2	2.0	5.61	0.50	1.9	10.0
20230809	NYC	3.3	1.8	237 ± 37	157 ± 8.0	296	48	949	5.5	17.5 ± 4.5	2.0	9.39	2.00	2.4	8.0
20230812	Chi	2.4	2.3	284 ± 99	89.2 ± 9.0	295	64	948	8.6	19.8 ± 7.1	2.0	6.0	1.50	2.6	10.3
20230815	Chi	9.0	2.6	264 ± 67	171 ± 22	292	70	956	6.2	13.7 ± 3.4	2.0	8.33	1.50	1.8	10.0
20230816	NYC	11.2	2.0	398 ± 200	140 ± 14	294	84	950	7.0	21.2 ± 5.9	2.0	7.38	3.00	1.6	9.9
20230823	LA	6.0	2.4	485 ± 170	125 ± 17	297	50	943	8.2	25.6 ± 11	2.0	6.30	3.00	2.1	9.7
20230825	LA	8.3	2.5	459 ± 99	115 ± 15	296	46	958	7.4	21.1 ± 8.0	2.0	6.98	3.00	1.9	9.8
20230826	LA	4.2	2.2	394 ± 127	104 ± 5	301	29	966	7.4	24.0 ± 8.8	2.0	6.98	3.00	2.3	9.9

Table S1. Flight Isoprene box model conditions. These parameters (except for NO, NO_2 and C_5H_8) were determined by averages of each parameter while the DC-8 was sampling in the boundary layer for each flight.

[a] IHN lifetime based on OH and hydrolysis loss assuming rapid 1,2-IHN hydrolysis loss rate from Vasquez et al. (*124*)
[b] ∑ISOPOOH+IEPOX lifetime based on OH ISOPOOH, OH IEPOX and heterogenous IEPOX loss.
[c] Missing bulk aerosol chemical data, average NYC aerosol properties used for this flight

RO_2 name in Pye et al. 2019 (*73*)	HO_2 product name	C*	f_p	NO product name	*C**	f_p
**1st generation RO_2**						
C10H17O5B9RO2	C10H18O5B9	2.40E+01	30.4%	C10H17NO6B9	260	3.9%
C10H17O5B12RO2	C10H18O5B12	1.40E-01	98.7%	C10H17NO6B12	1.9	84.7%
C10H17O5B13RO2	C10H18O5B13	0.16	98.5%	C10H17NO6B13	2	84.0%
C10H17O5B14RO2	C10H18O5B14	0.036	99.7%	C10H17NO6B14	0.5	95.5%
**2nd generation RO_2**						
C10H17O7B19RO2	C10H18O7B29	0.033	99.7%	C10H17NO8B29	0.43	96.1%
C10H17O9RO2	C10H18O9	0.0000093	100.0%	C10H17NO10	0.00014	100.0%
C10H17O7B20RO2	C10H18O7B20	0.0073	99.9%	C10H17NO8B20	0.1	99.1%
C10H17O7B29RO2	C10H18O7B29	0.033	99.7%	C10H17NO8B29	0.43	96.1%

Table S2. Alpha-pinene highly oxidized molecule (HOM) products for two generations of autoxidation. Saturation vapor pressure taken from Pye et al. (2019) (*73*).

Measurement	Method	Uncertainty	Reference
NO, NO_2, NO_y	NO-LIF	NO: ± 6%<br>NO_2: ± 9%<br>NO_y: ± 10%	(*85*)
O_3	Chemiluminescence	± 5% + 50 pptv	(*134*)
NH_3	QC-TILDAS	± 12%	(*135*)
IHN, ∑ISOPOOH+IEPOX	I^- CIMS	IHN: ± 30% + 2 pptv<br>∑ISOPOOH + IEPOX: ± 30% + 5 pptv	(*84*, *97*, *98*)
HNO_3, IHN	CF_3O^- CIMS	HNO_3: ± 20% + 25 pptv<br>IHN: ± 25% + 3 pptv	(*83*)
Isoprene, MVK, MACR	GC/MS	C_5H_8 : ± 15%<br>C_4H_6O: ± 20%	(*87*)
OH Reactivity	LIF	± 1%	(*88*)
HCHO	LIF	± 15% + 10 pptv	(*86*)
SO_4^{2-}, NO_3^-, NH_4^+, organic aerosols	AMS	± 35%	(*112–114*)
Na^+, K^+, Ca^{2+}, Mg^{2+}	PiLS-IC	± 10%	(*136*, *137*)
Single particle composition	PALMS-NG	± 1- 10%	(*115*, *116*)
Aerosol size distribution	NMASS, UHSAS, CMASS	Number: ± 6%<br>Surface Area: +18%/-36%<br>Volume: +23%/-51%	(*117–119*)
RH	DLH	± 15%	(*138*)
jNO_2	J-CAFS	jNO_2: ± 10%	(*139*)

Table S3. Summary of measurements used for AEROMMA analysis (box model constraints and in-situ observations).

Flight Date	City	∑ISOPOOH + IEPOX background (pptv) Transect #															
		1	2	3	4	5	6	7	8	9	10	11	12	13	14	15	Avg ± stdev
20230726	NYC	75	100	n/a	50	100	50	75	100	40	262	n/a	n/a	n/a	n/a	n/a	95 ± 67
20230728	NYC	160	100	140	n/a	60	270	225	215	135	350	275	200	n/a	n/a	n/a	194 ± 85
20230801	Chi	48	20	5	10	n/a	n/a	n/a	n/a	n/a	n/a	n/a	n/a	n/a	n/a	n/a	21 ± 10
20230802	Chi	75	45	50	40	70	35	10	10	n/a	n/a	n/a	n/a	n/a	n/a	n/a	42 ± 24
20230804	Tor	60	62	n/a	n/a	n/a	n/a	n/a	n/a	n/a	n/a	n/a	n/a	n/a	n/a	n/a	61 ± 1
20230805	Tor	40	28	30	40	24	30	n/a	n/a	n/a	n/a	n/a	n/a	n/a	n/a	n/a	32 ± 7
20230808	Chi	100	55	20	n/a	n/a	n/a	n/a	n/a	n/a	n/a	n/a	n/a	n/a	n/a	n/a	58 ± 40
20230809	NYC	175	n/a	175	270	295	265	260	240	n/a	n/a	n/a	n/a	n/a	n/a	n/a	240 ± 47
20230812	Chi	40	8	22	60	8	45	n/a	n/a	n/a	n/a	n/a	n/a	n/a	n/a	n/a	31 ± 21
20230815	Chi	45	30	40	38	32	30	60	58	45	36	n/a	n/a	n/a	n/a	n/a	41 ± 11
20230816	NYC	10	0	5	8	12	12	0	5	n/a	n/a	n/a	n/a	n/a	n/a	n/a	7 ± 5
20230823	LA	12	18	18	8	18	10	10	20	20	12	10	12	10	10	10	13 ± 4
20230825	LA	10	20	20	15	5	10	15	15	40	35	5	5	5	5	5	14 ± 11
20230826	LA	10	10	15	8	55	5	0	5	10	5	5	5	n/a	n/a	n/a	11 ± 14

Table S4. ∑ISOPOOH+IEPOX backgrounds determined for each transect during AEROMMA sampling.

Molecule	Rate coefficient (x 10^{-11} cm^3 $molec^{-1}$ s^{-1})	Lifetime (hr) ([OH] = 3 x 10^6 molec cm^{-3})	reference
1,2 IHN	3.0 ± 0.9	3.1	Teng et al. (*62*)
4,3 IHN	4.2 ± 0.9	2.2	Lee et al.(*140*)
1,2 ISOPOOH	7.5 ± 1.2	1.2	St Clair.(*141*)
4,3 ISOPOOH	11.8 ± 1.9	0.8	St Clair.(*141*)
trans-β IEPOX	0.98 ± 0.05	9.4	Bates.(*142*)
cis-β IEPOX	1.52 ± 0.07	6	Bates.(*142*)

Table S5. Select isoprene first and second-generation products OH rate constants and lifetimes

**Measurement**	**Method**	**Uncertainty**	**Reference**
NO, NO_2	NO-LIF	NO: ± 8%<br>NO_2: ± 15%	(*85*)
O_3	UV-Absorption	O_3: ± 5%	(*104*)
CO, CH_4	CRDS	CO: ± 1% + 15 ppbv<br>CH_4 : ± 1% + 2 ppbv	(*108*)
PAN, PPN, HONO, N_2O_5, $ClNO_2$	I^- CIMS	PAN: ± 7% + 64 pptv<br>PPN: ± 12% + 7 pptv<br>HONO: ± 25% + 5 pptv<br>N_2O_5: ± 15% + 0.04 pptv<br>$ClNO_2$: ± 15% + 0.06 pptv	(*97*, *98*)
sum monoterpenes, benzene, toluene, benzaldehyde, methanol, acetaldehyde, ethanol	PTR-ToF-MS	∑MT: ± 30%<br>Benzene: ± 20%<br>Toluene: ± 20%<br>Benzaldehyde: ± 30%<br>Methanol: ± 50%<br>Acetaldehyde: ± 20%<br>Ethanol: ± 50%<br>∑MVK/MACR: ± 30%	(*105–107*)
C_2H_4, C_2H_6, C_3H_8, i-C_4H_{10}, n-C_4H_{10}, C_5H_8, i-C_5H_{12}, n-C_5H_{12}, n-C_6H_{14}, n-$C_{10}H_{22}$, CH_3COCH_3,	GC/MS	C_xH_y : ± 15%<br>$C_xH_yO_z$: ± 20%	(*87*)
HCHO	CRDS	HCHO: ± 5%	(*108*, *109*)
jNO_2	Photomultiplier tube	jNO_2: ± 10%	(*110*)

Table S6. Summary of measurements used to constrain 0-D box model at Pasadena ground site

REFERENCES

1. EPA, "Policy Assessment for the Review of the Ozone National Ambient Air Quality Standards" (United States Environmental Protection Agency, 2020); www.epa.gov/naaqs/ozone-o3-standards-policy-assessments-current-review.

2. H. O. T. Pye, C. K. Ward-Caviness, B. N. Murphy, K. W. Appel, K. M. Seltzer, Secondary organic aerosol association with cardiorespiratory disease mortality in the United States. *Nat. Commun.* **12**, 7215 (2021).

3. W. L. Chameides, The photochemical role of tropospheric nitrogen oxides. *Geophys. Res. Lett.* **5**, 17–20 (1978).

4. A. J. Haagen-Smit, M. M. Fox, Photochemical ozone formation with hydrocarbons and automobile exhaust. *Air Repair* **4**, 105–136 (1954).

5. P. J. Crutzen, The influence of nitrogen oxides on the atmospheric ozone content. *Q. J. R. Meteorolog. Soc.* **96**, 320–325 (1970).

6. C. C. Womack, E. E. McDuffie, P. M. Edwards, R. Bares, J. A. Gouw, K. S. Docherty, W. P. Dubé, D. L. Fibiger, A. Franchin, J. B. Gilman, L. Goldberger, B. H. Lee, J. C. Lin, R. Long, A. M. Middlebrook, D. B. Millet, A. Moravek, J. G. Murphy, P. K. Quinn, T. P. Riedel, J. M. Roberts, J. A. Thornton, L. C. Valin, P. R. Veres, A. R. Whitehill, R. J. Wild, C. Warneke, B. Yuan, M. Baasandorj, S. S. Brown, An odd oxygen framework for wintertime ammonium nitrate aerosol pollution in urban areas: NO_x and VOC control as mitigation strategies. *Geophys. Res. Lett.* **46**, 4971–4979 (2019).

7. I. Pullinen, S. Schmitt, S. Kang, M. Sarrafzadeh, P. Schlag, S. Andres, E. Kleist, T. F. Mentel, F. Rohrer, M. Springer, R. Tillmann, J. Wildt, C. Wu, D. Zhao, A. Wahner, A. Kiendler-Scharr, Impact of NO_x on secondary organic aerosol (SOA) formation from α -pinene and β -pinene photooxidation: The role of highly oxygenated organic nitrates. *Atmos. Chem. Phys.* **20**, 10125–10147 (2020).

8. Z. Yang, N. T. Tsona, J. Li, S. Wang, L. Xu, B. You, L. Du, Effects of NO and SO_2 on the secondary organic aerosol formation from the photooxidation of 1,3,5-trimethylbenzene: A new source of organosulfates. *Environ. Pollut.* **264**, 114742 (2020).

9. J. D. Crounse, F. Paulot, H. G. Kjaergaard, P. O. Wennberg, Peroxy radical isomerization in the oxidation of isoprene. *Phys. Chem. Chem. Phys.* **13**, 13607 (2011).

10. J. D. Crounse, L. B. Nielsen, S. Jørgensen, H. G. Kjaergaard, P. O. Wennberg, Autoxidation of organic compounds in the atmosphere. *J. Phys. Chem. Lett.* **4**, 3513–3520 (2013).

11. M. Ehn, J. A. Thornton, E. Kleist, M. Sipilä, H. Junninen, I. Pullinen, M. Springer, F. Rubach, R. Tillmann, B. Lee, F. Lopez-Hilfiker, S. Andres, I.-H. Acir, M. Rissanen, T. Jokinen, S. Schobesberger, J. Kangasluoma, J. Kontkanen, T. Nieminen, T. Kurtén, L. B. Nielsen, S. Jørgensen, H. G. Kjaergaard, M. Canagaratna, M. D. Maso, T. Berndt, T. Petäjä, A. Wahner, V.-M. Kerminen, M. Kulmala, D. R. Worsnop, J. Wildt, T. F. Mentel, A large source of low-volatility secondary organic aerosol. *Nature* **506**, 476–479 (2014).

12. E. Praske, R. V. Otkjær, J. D. Crounse, J. C. Hethcox, B. M. Stoltz, H. G. Kjaergaard, P. O. Wennberg, Atmospheric autoxidation is increasingly important in urban and suburban North America. *Proc. Natl. Acad. Sci. U.S.A.* **115**, 64–69 (2018).

13. W. Chameides, R. Lindsay, J. Richardson, C. Kiang, The role of biogenic hydrocarbons in urban photochemical smog: Atlanta as a case study. *Science* **241**, 1473–1475 (1988).

14. J. H. Seinfeld, Urban air pollution: State of the science. *Science* **243**, 745–752 (1989).

15. D. Poppe, R. Koppmann, J. Rudolph, Ozone formation in biomass burning plumes: Influence of atmospheric dilution. *Geophys. Res. Lett.* **25**, 3823–3826 (1998).

16. P. M. Edwards, S. S. Brown, J. M. Roberts, R. Ahmadov, R. M. Banta, J. A. deGouw, W. P. Dubé, R. A. Field, J. H. Flynn, J. B. Gilman, M. Graus, D. Helmig, A. Koss, A. O. Langford, B. L. Lefer, B. M. Lerner, R. Li, S.-M. Li, S. A. McKeen, S. M. Murphy, D. D. Parrish, C. J. Senff, J. Soltis, J. Stutz, C. Sweeney, C. R. Thompson, M. K. Trainer, C. Tsai, P. R. Veres, R. A.

Washenfelder, C. Warneke, R. J. Wild, C. J. Young, B. Yuan, R. Zamora, High winter ozone pollution from carbonyl photolysis in an oil and gas basin. *Nature* **514**, 351–354 (2014).

17. G. M. Mazzuca, X. Ren, C. P. Loughner, M. Estes, J. H. Crawford, K. E. Pickering, A. J. Weinheimer, R. R. Dickerson, Ozone production and its sensitivity to NO_x and VOCs: Results from the DISCOVER-AQ field experiment, Houston 2013. *Atmos. Chem. Phys.* **16**, 14463–14474 (2016).

18. E. E. McDuffie, P. M. Edwards, J. B. Gilman, B. M. Lerner, W. P. Dubé, M. Trainer, D. E. Wolfe, W. M. Angevine, J. deGouw, E. J. Williams, A. G. Tevlin, J. G. Murphy, E. V. Fischer, S. McKeen, T. B. Ryerson, J. Peischl, J. S. Holloway, K. Aikin, A. O. Langford, C. J. Senff, R. J. Alvarez, S. R. Hall, K. Ullmann, K. O. Lantz, S. S. Brown, Influence of oil and gas emissions on summertime ozone in the Colorado Northern Front Range. *J. Geophys. Res. Atmos.* **121**, 8712–8729 (2016).

19. M. A. Robinson, Z. C. J. Decker, K. C. Barsanti, M. M. Coggon, F. M. Flocke, A. Franchin, C. D. Fredrickson, J. B. Gilman, G. I. Gkatzelis, C. D. Holmes, A. Lamplugh, A. Lavi, A. M. Middlebrook, D. M. Montzka, B. B. Palm, J. Peischl, B. Pierce, R. H. Schwantes, K. Sekimoto, V. Selimovic, G. S. Tyndall, J. A. Thornton, P. Van Rooy, C. Warneke, A. J. Weinheimer, S. S. Brown, Variability and time of day dependence of ozone photochemistry in Western wildfire plumes. *Environ. Sci. Technol.* **55**, 10280–10290 (2021).

20. L. I. Kleinman, P. H. Daum, J. H. Lee, Y.-N. Lee, L. J. Nunnermacker, S. R. Springston, L. Newman, J. Weinstein-Lloyd, S. Sillman, Dependence of ozone production on NO and hydrocarbons in the troposphere. *Geophys. Res. Lett.* **24**, 2299–2302 (1997).

21. L. I. Kleinman, The dependence of tropospheric ozone production rate on ozone precursors. *Atmos. Environ.* **39**, 575–586 (2005).

22. P. M. Edwards, C. J. Young, K. Aikin, J. deGouw, W. P. Dubé, F. Geiger, J. Gilman, D. Helmig, J. S. Holloway, J. Kercher, B. Lerner, R. Martin, R. McLaren, D. D. Parrish, J. Peischl, J. M. Roberts, T. B. Ryerson, J. Thornton, C. Warneke, E. J. Williams, S. S. Brown, Ozone

photochemistry in an oil and natural gas extraction region during winter: Simulations of a snow-free season in the Uintah Basin, Utah. *Atmos. Chem. Phys.* **13**, 8955–8971 (2013).

23. P. S. Rickly, M. M. Coggon, K. C. Aikin, R. J. Alvarez, S. Baidar, J. B. Gilman, G. I. Gkatzelis, C. Harkins, J. He, A. Lamplugh, A. O. Langford, B. C. McDonald, J. Peischl, M. A. Robinson, A. W. Rollins, R. H. Schwantes, C. J. Senff, C. Warneke, S. S. Brown, Influence of wildfire on urban ozone: An observationally constrained box modeling study at a site in the Colorado front range. *Environ. Sci. Technol.* **57**, 1257–1267 (2023).

24. A. H. Souri, M. S. Johnson, G. M. Wolfe, J. H. Crawford, A. Fried, A. Wisthaler, W. H. Brune, D. R. Blake, A. J. Weinheimer, T. Verhoelst, S. Compernolle, G. Pinardi, C. Vigouroux, B. Langerock, S. Choi, L. Lamsal, L. Zhu, S. Sun, R. C. Cohen, K.-E. Min, C. Cho, S. Philip, X. Liu, K. Chance, Characterization of errors in satellite-based HCHO / NO_2 tropospheric column ratios with respect to chemistry, column-to-PBL translation, spatial representation, and retrieval uncertainties. *Atmos. Chem. Phys.* **23**, 1963–1986 (2023).

25. S. Sillman, The use of NO_y, H_2O_2, and HNO_3 as indicators for ozone-NO_x-hydrocarbon sensitivity in urban locations. *J. Geophys. Res. Atmos.* **100**, 14175–14188 (1995).

26. R. V. Martin, A. M. Fiore, A. Van Donkelaar, Space-based diagnosis of surface ozone sensitivity to anthropogenic emissions. *Geophys. Res. Lett.* **31**, 2004GL019416 (2004).

27. B. N. Duncan, Y. Yoshida, J. R. Olson, S. Sillman, R. V. Martin, L. Lamsal, Y. Hu, K. E. Pickering, C. Retscher, D. J. Allen, J. H. Crawford, Application of OMI observations to a space-based indicator of NO_x and VOC controls on surface ozone formation. *Atmos. Environ.* **44**, 2213–2223 (2010).

28. W. L. Chameides, F. Fehsenfeld, M. O. Rodgers, C. Cardelino, J. Martinez, D. Parrish, W. Lonneman, D. R. Lawson, R. A. Rasmussen, P. Zimmerman, J. Greenberg, P. Mlddleton, T. Wang, Ozone precursor relationships in the ambient atmosphere. *J. Geophys. Res. Atmos.* **97**, 6037–6055 (1992).

29. R. V. Martin, D. J. Jacob, K. Chance, T. P. Kurosu, P. I. Palmer, M. J. Evans, Global inventory of nitrogen oxide emissions constrained by space-based observations of NO_2 columns. *J. Geophys. Res. Atmos.* **108**, 2003JD003453 (2003).

30. M. Martinez, H. Harder, T. A. Kovacs, J. B. Simpas, J. Bassis, R. Lesher, W. H. Brune, G. J. Frost, E. J. Williams, C. A. Stroud, B. T. Jobson, J. M. Roberts, S. R. Hall, R. E. Shetter, B. Wert, A. Fried, B. Alicke, J. Stutz, V. L. Young, A. B. White, R. J. Zamora, OH and HO_2 concentrations, sources, and loss rates during the Southern Oxidants Study in Nashville, Tennessee, summer 1999. *J. Geophys. Res. Atmos.* **108**, 4617 (2003).

31. J. R. Schroeder, J. H. Crawford, A. Fried, J. Walega, A. Weinheimer, A. Wisthaler, M. Müller, T. Mikoviny, G. Chen, M. Shook, D. R. Blake, G. S. Tonnesen, New insights into the column CH_2O/NO_2 ratio as an indicator of near-surface ozone sensitivity: CH_2O/NO_2 as indicator of O_3 sensitivity. *J. Geophys. Res. Atmos.* **122**, 8885–8907 (2017).

32. L. Xu, J. D. Crounse, K. T. Vasquez, H. Allen, P. O. Wennberg, I. Bourgeois, S. S. Brown, P. Campuzano-Jost, M. M. Coggon, J. H. Crawford, J. P. DiGangi, G. S. Diskin, A. Fried, E. M. Gargulinski, J. B. Gilman, G. I. Gkatzelis, H. Guo, J. W. Hair, S. R. Hall, H. A. Halliday, T. F. Hanisco, R. A. Hannun, C. D. Holmes, L. G. Huey, J. L. Jimenez, A. Lamplugh, Y. R. Lee, J. Liao, J. Lindaas, J. A. Neuman, J. B. Nowak, J. Peischl, D. A. Peterson, F. Piel, D. Richter, P. S. Rickly, M. A. Robinson, A. W. Rollins, T. B. Ryerson, K. Sekimoto, V. Selimovic, T. Shingler, A. J. Soja, J. M. St. Clair, D. J. Tanner, K. Ullmann, P. R. Veres, J. Walega, C. Warneke, R. A. Washenfelder, P. Weibring, A. Wisthaler, G. M. Wolfe, C. C. Womack, R. J. Yokelson, Ozone chemistry in western U.S. wildfire plumes. *Sci. Adv.* **7**, eabl3648 (2021).

33. E. A. Marais, D. J. Jacob, J. L. Jimenez, P. Campuzano-Jost, D. A. Day, W. Hu, J. Krechmer, L. Zhu, P. S. Kim, C. C. Miller, J. A. Fisher, K. Travis, K. Yu, T. F. Hanisco, G. M. Wolfe, H. L. Arkinson, H. O. T. Pye, K. D. Froyd, J. Liao, V. F. McNeill, Aqueous-phase mechanism for secondary organic aerosol formation from isoprene: Application to the southeast United States and co-benefit of SO_2 emission controls. *Atmos. Chem. Phys.* **16**, 1603–1618 (2016).

34. H. S. Kenagy, C. L. Heald, N. Tahsini, M. B. Goss, J. H. Kroll, Can we achieve atmospheric chemical environments in the laboratory? An integrated model-measurement approach to chamber SOA studies. *Sci. Adv.* **10**, eado1482 (2024).

35. M. J. Newland, D. J. Bryant, R. E. Dunmore, T. J. Bannan, W. J. F. Acton, B. Langford, J. R. Hopkins, F. A. Squires, W. Dixon, W. S. Drysdale, P. D. Ivatt, M. J. Evans, P. M. Edwards, L. K. Whalley, D. E. Heard, E. J. Slater, R. Woodward-Massey, C. Ye, A. Mehra, S. D. Worrall, A. Bacak, H. Coe, C. J. Percival, C. N. Hewitt, J. D. Lee, T. Cui, J. D. Surratt, X. Wang, A. C. Lewis, A. R. Rickard, J. F. Hamilton, Low-NO atmospheric oxidation pathways in a polluted megacity. *Atmos. Chem. Phys.* **21**, 1613–1625 (2021).

36. C. M. Nussbaumer, H. Fischer, J. Lelieveld, A. Pozzer, What controls ozone sensitivity in the upper tropical troposphere? *Atmos. Chem. Phys.* **23**, 12651–12669 (2023).

37. C. M. Nussbaumer, M. Kohl, A. Pozzer, I. Tadic, R. Rohloff, D. Marno, H. Harder, H. Ziereis, A. Zahn, F. Obersteiner, A. Hofzumahaus, H. Fuchs, C. Künstler, W. H. Brune, T. B. Ryerson, J. Peischl, C. R. Thompson, I. Bourgeois, J. Lelieveld, H. Fischer, Ozone formation sensitivity to precursors and lightning in the tropical troposphere based on airborne observations. *J. Geophys. Res. Atmos.* **129**, e2024JD041168 (2024).

38. F. Bianchi, T. Kurtén, M. Riva, C. Mohr, M. P. Rissanen, P. Roldin, T. Berndt, J. D. Crounse, P. O. Wennberg, T. F. Mentel, J. Wildt, H. Junninen, T. Jokinen, M. Kulmala, D. R. Worsnop, J. A. Thornton, N. Donahue, H. G. Kjaergaard, M. Ehn, Highly oxygenated organic molecules (HOM) from gas-phase autoxidation involving peroxy radicals: A key contributor to atmospheric aerosol. *Chem. Rev.* **119**, 3472–3509 (2019).

39. M. Rissanen, Anthropogenic volatile organic compound (AVOC) autoxidation as a source of highly oxygenated organic molecules (HOM). *J. Phys. Chem. A* **125**, 9027–9039 (2021).

40. M. Wang, D. Chen, M. Xiao, Q. Ye, D. Stolzenburg, V. Hofbauer, P. Ye, A. L. Vogel, R. L. Mauldin, A. Amorim, A. Baccarini, B. Baumgartner, S. Brilke, L. Dada, A. Dias, J. Duplissy, H. Finkenzeller, O. Garmash, X.-C. He, C. R. Hoyle, C. Kim, A. Kvashnin, K. Lehtipalo, L. Fischer, U. Molteni, T. Petäjä, V. Pospisilova, L. L. J. Quéléver, M. Rissanen, M. Simon, C.

Tauber, A. Tomé, A. C. Wagner, L. Weitz, R. Volkamer, P. M. Winkler, J. Kirkby, D. R. Worsnop, M. Kulmala, U. Baltensperger, J. Dommen, I. El-Haddad, N. M. Donahue, Photo-oxidation of aromatic hydrocarbons produces low-volatility organic compounds. *Environ. Sci. Technol.* **54**, 7911–7921 (2020).

41. M. Xiao, M. Wang, B. Mentler, O. Garmash, H. Lamkaddam, U. Molteni, M. Simon, L. Ahonen, A. Amorim, A. Baccarini, P. S. Bauer, D. Chen, R. Chiu, L. Dada, J. Duplissy, H. Finkenzeller, L. Fischer, X.-C. He, M. Heinritzi, V. Hofbauer, C. Kim, A. Kürten, A. Kvashnin, K. Lehtipalo, Y. Liu, H. Mai, V. Makhmutov, S. Mathot, R. Mauldin, A. Onnela, T. Petäjä, L. L. J. Quéléver, M. Rissanen, S. Schuchmann, M. Sipilä, D. Stolzenburg, Y. Stozhkov, C. Tauber, A. Tomé, R. Wagner, C. Yan, B. Yang, P. Ye, Q. Zha, J. Curtius, A. Hansel, J. Kirkby, M. Kulmala, R. Volkamer, P. M. Winkler, D. R. Worsnop, W. Nie, N. M. Donahue, C. R. Hoyle, J. Jiang, U. Baltensperger, J. Dommen, I. El Haddad, Anthropogenic organic aerosol in Europe produced mainly through second-generation oxidation. *Nat. Geosci.* **18**, 239–245 (2025).

42. J. Peeters, T. L. Nguyen, L. Vereecken, HO_x radical regeneration in the oxidation of isoprene. *Phys. Chem. Chem. Phys.* **11**, 5935–5939 (2009).

43. R. Atkinson, Atmospheric chemistry of VOCs and NO_x. *Atmos. Environ.* **34**, 2063–2101 (2000).

44. M. Claeys, B. Graham, G. Vas, W. Wang, R. Vermeylen, V. Pashynska, J. Cafmeyer, P. Guyon, M. O. Andreae, P. Artaxo, W. Maenhaut, Formation of secondary organic aerosols through photooxidation of isoprene. *Science* **303**, 1173–1176 (2004).

45. A. G. Carlton, C. Wiedinmyer, J. H. Kroll, A review of secondary organic aerosol (SOA) formation from isoprene. *Atmos. Chem. Phys.* **9**, 4987–5005 (2009).

46. M. Shrivastava, C. D. Cappa, J. Fan, A. H. Goldstein, A. B. Guenther, J. L. Jimenez, C. Kuang, A. Laskin, S. T. Martin, N. L. Ng, T. Petaja, J. R. Pierce, P. J. Rasch, P. Roldin, J. H. Seinfeld, J. Shilling, J. N. Smith, J. A. Thornton, R. Volkamer, J. Wang, D. R. Worsnop, R. A. Zaveri, A. Zelenyuk, Q. Zhang, Recent advances in understanding secondary organic aerosol: Implications for global climate forcing. *Rev. Geophys.* **55**, 509–559 (2017).

47. S. Liu, B. Barletta, R. S. Hornbrook, A. Fried, J. Peischl, S. Meinardi, M. Coggon, A. Lamplugh, J. B. Gilman, G. I. Gkatzelis, C. Warneke, E. C. Apel, A. J. Hills, I. Bourgeois, J. Walega, P. Weibring, D. Richter, T. Kuwayama, M. FitzGibbon, D. Blake, Composition and reactivity of volatile organic compounds in the South Coast Air Basin and San Joaquin Valley of California. *Atmos. Chem. Phys.* **22**, 10937–10954 (2022).

48. C. Cao, D. R. Gentner, R. Commane, R. Toledo-Crow, L. D. Schiferl, J. E. Mak, Policy-related gains in urban air quality may be offset by increased emissions in a warming climate. *Environ. Sci. Technol.* **57**, 9683–9692 (2023).

49. M. M. Coggon, G. I. Gkatzelis, B. C. McDonald, J. B. Gilman, R. H. Schwantes, N. Abuhassan, K. C. Aikin, M. F. Arend, T. A. Berkoff, S. S. Brown, T. L. Campos, R. R. Dickerson, G. Gronoff, J. F. Hurley, G. Isaacman-VanWertz, A. R. Koss, M. Li, S. A. McKeen, F. Moshary, J. Peischl, V. Pospisilova, X. Ren, A. Wilson, Y. Wu, M. Trainer, C. Warneke, Volatile chemical product emissions enhance ozone and modulate urban chemistry. *Proc. Natl. Acad. Sci. U.S.A.* **118**, e2026653118 (2021).

50. S. Gu, A. Guenther, C. Faiola, Effects of anthropogenic and biogenic volatile organic compounds on Los Angeles air quality. *Environ. Sci. Technol.* **55**, 12191–12201 (2021).

51. E. Y. Pfannerstill, C. Arata, Q. Zhu, B. C. Schulze, R. Woods, C. Harkins, R. H. Schwantes, B. C. McDonald, J. H. Seinfeld, A. Bucholtz, R. C. Cohen, A. H. Goldstein, Comparison between spatially resolved airborne flux measurements and emission inventories of volatile organic compounds in Los Angeles. *Environ. Sci. Technol.* **57**, 15533–15545 (2023).

52. C. E. Stockwell, M. M. Coggon, R. H. Schwantes, C. Harkins, B. Verreyken, C. Lyu, Q. Zhu, L. Xu, J. B. Gilman, A. Lamplugh, J. Peischl, M. A. Robinson, P. R. Veres, M. Li, A. W. Rollins, K. Zuraski, S. Baidar, S. Liu, T. Kuwayama, S. S. Brown, B. C. McDonald, C. Warneke, Urban ozone formation and sensitivities to volatile chemical products, cooking emissions, and NO_x upwind of and within two Los Angeles Basin cities. *Atmos. Chem. Phys.* **25**, 1121–1143 (2025).

53. M. P. Vermeuel, G. A. Novak, H. D. Alwe, D. D. Hughes, R. Kaleel, A. F. Dickens, D. Kenski, A. C. Czarnetzki, E. A. Stone, C. O. Stanier, R. B. Pierce, D. B. Millet, T. H. Bertram,

Sensitivity of ozone production to NO_x and VOC along the Lake Michigan Coastline. *J. Geophys. Res. Atmos.* **124**, 10989–11006 (2019).

54. J. A. Geddes, S. E. Pusede, A. Y. H. Wong, Changes in the relative importance of biogenic isoprene and soil NO_x emissions on ozone concentrations in nonattainment areas of the United States. *J. Geophys. Res. Atmos.* **127**, e2021JD036361 (2022).

55. C. M. Nussbaumer, R. C. Cohen, The role of temperature and NO_x in ozone trends in the Los Angeles Basin. *Environ. Sci. Technol.* **54**, 15652–15659 (2020).

56. H. O. T. Pye, A. W. H. Chan, M. P. Barkley, J. H. Seinfeld, Global modeling of organic aerosol: The importance of reactive nitrogen (NO_x and NO_3). *Atmos. Chem. Phys.* **10**, 11261–11276 (2010).

57. P. O. Wennberg, K. H. Bates, J. D. Crounse, L. G. Dodson, R. C. McVay, L. A. Mertens, T. B. Nguyen, E. Praske, R. H. Schwantes, M. D. Smarte, J. M. St Clair, A. P. Teng, X. Zhang, J. H. Seinfeld, Gas-phase reactions of isoprene and its major oxidation products. *Chem. Rev.* **118**, 3337–3390 (2018).

58. M. E. Jenkin, J. C. Young, A. R. Rickard, The MCM v3.3.1 degradation scheme for isoprene. *Atmos. Chem. Phys.* **15**, 11433–11459 (2015).

59. A. E. Sebol, T. P. Canty, G. M. Wolfe, R. Hannun, A. M. Ring, X. Ren, Exploring ozone production sensitivity to NO_x and VOCs in the New York City airshed in the spring and summers of 2017–2019. *Atmos. Environ.* **324**, 120417 (2024).

60. J. J. M. Acdan, R. B. Pierce, A. F. Dickens, Z. Adelman, T. Nergui, Examining TROPOMI formaldehyde to nitrogen dioxide ratios in the Lake Michigan region: Implications for ozone exceedances. *Atmos. Chem. Phys.* **23**, 7867–7885 (2023).

61. X. Jin, A. Fiore, K. F. Boersma, I. D. Smedt, L. Valin, Inferring changes in summertime surface ozone–NO_x–VOC chemistry over U.S. Urban areas from two decades of satellite and ground-based observations. *Environ. Sci. Technol.* **54**, 6518–6529 (2020).

62. A. P. Teng, J. D. Crounse, P. O. Wennberg, Isoprene peroxy radical dynamics. *J. Am. Chem. Soc.* **139**, 5367–5377 (2017).

63. M. E. Jenkin, S. M. Saunders, M. J. Pilling, The tropospheric degradation of volatile organic compounds: A protocol for mechanism development. *Atmos. Environ.* **31**, 81–104 (1997).

64. G. S. Tyndall, R. A. Cox, C. Granier, R. Lesclaux, G. K. Moortgat, M. J. Pilling, A. R. Ravishankara, T. J. Wallington, Atmospheric chemistry of small organic peroxy radicals. *J. Geophys. Res. Atmos.* **106**, 12157–12182 (2001).

65. M. E. Jenkin, R. Valorso, B. Aumont, A. R. Rickard, Estimation of rate coefficients and branching ratios for reactions of organic peroxy radicals for use in automated mechanism construction. *Atmos. Chem. Phys.* **19**, 7691–7717 (2019).

66. L. Vereecken, J.-F. Müller, J. Peeters, Low-volatility poly-oxygenates in the OH-initiated atmospheric oxidation of α-pinene: Impact of non-traditional peroxyl radical chemistry. *Phys. Chem. Chem. Phys.* **9**, 5241 (2007).

67. L. Xu, K. H. Møller, J. D. Crounse, R. V. Otkjær, H. G. Kjaergaard, P. O. Wennberg, Unimolecular reactions of peroxy radicals formed in the oxidation of α-pinene and β-pinene by hydroxyl radicals. *J. Phys. Chem. A* **123**, 1661–1674 (2019).

68. T. Berndt, Peroxy radical processes and product formation in the OH radical-initiated oxidation of α-pinene for near-atmospheric conditions. *J. Phys. Chem. A* **125**, 9151–9160 (2021).

69. H. Yu, K. H. Møller, R. S. Buenconsejo, J. D. Crounse, H. G. Kjaergaard, P. O. Wennberg, Atmospheric photo-oxidation of 2-ethoxyethanol: Autoxidation chemistry of glycol ethers. *J. Phys. Chem. A* **127**, 9564–9579 (2023).

70. T. Berndt, S. Richters, T. Jokinen, N. Hyttinen, T. Kurtén, R. V. Otkjær, H. G. Kjaergaard, F. Stratmann, H. Herrmann, M. Sipilä, M. Kulmala, M. Ehn, Hydroxyl radical-induced formation of highly oxidized organic compounds. *Nat. Commun.* **7**, 13677 (2016).

71. M. Färber, H. Fuchs, B. Bohn, P. T. M. Carlsson, G. I. Gkatzelis, A. C. Marcillo Lara, F. Rohrer, L. Vereecken, S. Wedel, A. Wahner, A. Novelli, Effect of the Alkoxy radical chemistry on the ozone formation from anthropogenic organic compounds investigated in chamber experiments. *ACS ES&T Air* **1**, 1096–1111 (2024).

72. S. Barua, S. Iyer, A. Kumar, P. Seal, M. Rissanen, An aldehyde as a rapid source of secondary aerosol precursors: Theoretical and experimental study of hexanal autoxidation. *Atmos. Chem. Phys.* **23**, 10517–10532 (2023).

73. H. O. T. Pye, E. L. D'Ambro, B. H. Lee, S. Schobesberger, M. Takeuchi, Y. Zhao, F. Lopez-Hilfiker, J. Liu, J. E. Shilling, J. Xing, R. Mathur, A. M. Middlebrook, J. Liao, A. Welti, M. Graus, C. Warneke, J. A. De Gouw, J. S. Holloway, T. B. Ryerson, I. B. Pollack, J. A. Thornton, Anthropogenic enhancements to production of highly oxygenated molecules from autoxidation. *Proc. Natl. Acad. Sci. U.S.A.* **116**, 6641–6646 (2019).

74. R. Xu, J. A. Thornton, B. H. Lee, Y. Zhang, L. Jaeglé, F. D. Lopez-Hilfiker, P. Rantala, T. Petäjä, Global simulations of monoterpene-derived peroxy radical fates and the distributions of highly oxygenated organic molecules (HOMs) and accretion products. *Atmos. Chem. Phys.* **22**, 5477–5494 (2022).

75. H. O. T. Pye, B. K. Place, B. N. Murphy, K. M. Seltzer, E. L. D'Ambro, C. Allen, I. R. Piletic, S. Farrell, R. H. Schwantes, M. M. Coggon, E. Saunders, L. Xu, G. Sarwar, W. T. Hutzell, K. M. Foley, G. Pouliot, J. Bash, W. R. Stockwell, Linking gas, particulate, and toxic endpoints to air emissions in the Community Regional Atmospheric Chemistry Multiphase Mechanism (CRACMM). *Atmos. Chem. Phys.* **23**, 5043–5099 (2023).

76. M. Schervish, N. M. Donahue, Peroxy radical chemistry and the volatility basis set. *Atmos. Chem. Phys.* **20**, 1183–1199 (2020).

77. B. C. Schulze, "Insights into the sources of atmospheric aerosols and greenhouse gases in California," thesis, California Institute of Technology, Pasadena, CA (2023).

78. T. Hass-Mitchell, T. Joo, M. Rogers, B. A. Nault, C. Soong, M. Tran, M. Seo, J. E. Machesky, M. Canagaratna, J. Roscioli, M. S. Claflin, B. M. Lerner, D. C. Blomdahl, P. K. Misztal, N. L. Ng, A. M. Dillner, R. Bahreini, A. Russell, J. E. Krechmer, A. Lambe, D. R. Gentner, Increasing contributions of temperature-dependent oxygenated organic aerosol to summertime particulate matter in New York City. *ACS ES&T Air* **1**, 113–128 (2024).

79. E. Y. Pfannerstill, C. Arata, Q. Zhu, B. C. Schulze, R. Ward, R. Woods, C. Harkins, R. H. Schwantes, J. H. Seinfeld, A. Bucholtz, R. C. Cohen, A. H. Goldstein, Temperature-dependent emissions dominate aerosol and ozone formation in Los Angeles. *Science* **384**, 1324–1329 (2024).

80. P. S. Romer Present, A. Zare, R. C. Cohen, The changing role of organic nitrates in the removal and transport of NO_x. *Atmos. Chem. Phys.* **20**, 267–279 (2020).

81. W. S. Chace, C. Womack, K. Ball, K. H. Bates, B. Bohn, M. Coggon, J. D. Crounse, H. Fuchs, J. Gilman, G. I. Gkatzelis, C. M. Jernigan, G. A. Novak, A. Novelli, J. Peischl, I. Pollack, M. A. Robinson, A. Rollins, N. B. Schafer, R. H. Schwantes, M. Selby, A. Stainsby, C. Stockwell, R. Taylor, V. Treadaway, P. R. Veres, C. Warneke, E. Waxman, P. O. Wennberg, G. M. Wolfe, L. Xu, K. Zuraski, S. S. Brown, Ozone production efficiencies in the three largest United States cities from airborne measurements. *Environ. Sci. Technol.* **59**, 13306–13318 (2025).

82. J. D. Crounse, K. A. McKinney, A. J. Kwan, P. O. Wennberg, Measurement of gas-phase hydroperoxides by chemical ionization mass spectrometry. *Anal. Chem.* **78**, 6726–6732 (2006).

83. H. M. Allen, J. D. Crounse, M. J. Kim, A. P. Teng, E. A. Ray, K. McKain, C. Sweeney, P. O. Wennberg, H_2O_2 and CH_3OOH (MHP) in the remote atmosphere: 1. Global distribution and regional influences. *J. Geophys. Res. Atmos.* **127**, 1–14 (2022).

84. M. A. Robinson, J. M. Roberts, J. A. Neuman, C. M. Jernigan, L. Xu, M. M. Coggon, C. E. Stockwell, C. Warneke, J. Peischl, J. B. Gilman, A. Lamplugh, A. W. Rollins, K. Zuraski, J. C. Rivera-Rios, Y. Wang, N. L. Ng, S. Liu, S. S. Brown, P. R. Veres, Online calibration of a chemical ionization mass spectrometer for multifunctional biogenic organic nitrates. *ACS ES&T Air* **1**, 1066–1083 (2024).

85. A. W. Rollins, P. S. Rickly, R.-S. Gao, T. B. Ryerson, S. S. Brown, J. Peischl, I. Bourgeois, Single-photon laser-induced fluorescence detection of nitric oxide at sub-parts-per-trillion mixing ratios. *Atmos. Meas. Tech.* **13**, 2425–2439 (2020).

86. M. Cazorla, G. M. Wolfe, S. A. Bailey, A. K. Swanson, H. L. Arkinson, T. F. Hanisco, A new airborne laser-induced fluorescence instrument for in situ detection of formaldehyde throughout the troposphere and lower stratosphere. *Atmos. Meas. Tech.* **8**, 541–552 (2015).

87. B. M. Lerner, J. B. Gilman, K. C. Aikin, E. L. Atlas, P. D. Goldan, M. Graus, R. Hendershot, G. A. Isaacman-VanWertz, A. Koss, W. C. Kuster, R. A. Lueb, R. J. McLaughlin, J. Peischl, D. Sueper, T. B. Ryerson, T. W. Tokarek, C. Warneke, B. Yuan, J. A. de Gouw, An improved, automated whole air sampler and gas chromatography mass spectrometry analysis system for volatile organic compounds in the atmosphere. *Atmos. Meas. Tech.* **10**, 291–313 (2017).

88. H. Fuchs, A. Stainsby, F. Berg, R. Dubus, M. Färber, A. Hofzumahaus, F. Holland, K. H. Bates, S. S. Brown, M. M. Coggon, G. S. Diskin, G. I. Gkatzelis, C. M. Jernigan, J. Peischl, M. A. Robinson, A. W. Rollins, N. B. Schafer, R. H. Schwantes, C. E. Stockwell, P. R. Veres, C. Warneke, E. M. Waxman, L. Xu, K. Zuraski, A. Wahner, A. Novelli, Advances in an OH reactivity instrument for airborne field measurements. *Atmos. Meas. Tech.* **18**, 881–895 (2025).

89. G. Marsaglia, W. W. Tsang, A fast, easily implemented method for sampling from decreasing or symmetric unimodal density functions. *SIAM J. Sci. Stat. Comput.* **5**, 349–359 (1984).

90. M. Evans, N. Hastings, B. Peacock, *Statistical Distributions* (Wiley, ed. 2, 1993).

91. J. M. Roberts, M. Marchewka, S. B. Bertman, P. Goldan, W. Kuster, J. De Gouw, C. Warneke, E. Williams, B. Lerner, P. Murphy, E. Apel, F. C. Fehsenfeld, Analysis of the isoprene chemistry observed during the New England Air Quality Study (NEAQS) 2002 intensive experiment. *J. Geophys. Res. Atmos.* **111**, 2006JD007570 (2006).

92. G. M. Wolfe, J. Kaiser, T. F. Hanisco, F. N. Keutsch, J. A. De Gouw, J. B. Gilman, M. Graus, C. D. Hatch, J. Holloway, L. W. Horowitz, B. H. Lee, B. M. Lerner, F. Lopez-Hilifiker, J. Mao, M. R. Marvin, J. Peischl, I. B. Pollack, J. M. Roberts, T. B. Ryerson, J. A. Thornton, P. R. Veres,

C. Warneke, Formaldehyde production from isoprene oxidation across NO_x regimes. *Atmos. Chem. Phys.* **16**, 2597–2610 (2016).

93. A. Novelli, L. Vereecken, B. Bohn, H.-P. Dorn, G. I. Gkatzelis, A. Hofzumahaus, F. Holland, D. Reimer, F. Rohrer, S. Rosanka, D. Taraborrelli, R. Tillmann, R. Wegener, Z. Yu, A. Kiendler-Scharr, A. Wahner, H. Fuchs, Importance of isomerization reactions for OH radical regeneration from the photo-oxidation of isoprene investigated in the atmospheric simulation chamber SAPHIR. *Atmos. Chem. Phys.* **20**, 3333–3355 (2020).

94. T. B. Ryerson, A. E. Andrews, W. M. Angevine, T. S. Bates, C. A. Brock, B. Cairns, R. C. Cohen, O. R. Cooper, J. A. de Gouw, F. C. Fehsenfeld, R. A. Ferrare, M. L. Fischer, R. C. Flagan, A. H. Goldstein, J. W. Hair, R. M. Hardesty, C. A. Hostetler, J. L. Jimenez, A. O. Langford, E. McCauley, S. A. McKeen, L. T. Molina, A. Nenes, S. J. Oltmans, D. D. Parrish, J. R. Pederson, R. B. Pierce, K. Prather, P. K. Quinn, J. H. Seinfeld, C. J. Senff, A. Sorooshian, J. Stutz, J. D. Surratt, M. Trainer, R. Volkamer, E. J. Williams, S. C. Wofsy, The 2010 California Research at the Nexus of Air Quality and Climate Change (CalNex) field study: CalNex 2010 FIELD PROJECT OVERVIEW. *J. Geophys. Res. Atmos.* **118**, 5830–5866 (2013).

95. L. Xu, M. M. Coggon, C. E. Stockwell, J. B. Gilman, M. A. Robinson, M. Breitenlechner, A. Lamplugh, J. D. Crounse, P. O. Wennberg, J. A. Neuman, G. A. Novak, P. R. Veres, S. S. Brown, C. Warneke, Chemical ionization mass spectrometry utilizing ammonium ions (NH_4^+ CIMS) for measurements of organic compounds in the atmosphere. *Atmos. Meas. Tech.* **15**, 7353–7373 (2022).

96. J. A. De Gouw, J. B. Gilman, S. -W. Kim, S. L. Alvarez, S. Dusanter, M. Graus, S. M. Griffith, G. Isaacman-VanWertz, W. C. Kuster, B. L. Lefer, B. M. Lerner, B. C. McDonald, B. Rappenglück, J. M. Roberts, P. S. Stevens, J. Stutz, R. Thalman, P. R. Veres, R. Volkamer, C. Warneke, R. A. Washenfelder, C. J. Young, Chemistry of volatile organic compounds in the Los Angeles Basin: Formation of oxygenated compounds and determination of emission ratios. *J. Geophys. Res. Atmos.* **123**, 2298–2319 (2018).

97. P. R. Veres, J. A. Neuman, T. H. Bertram, E. Assaf, G. M. Wolfe, C. J. Williamson, B. Weinzierl, S. Tilmes, C. R. Thompson, A. B. Thames, J. C. Schroder, A. Saiz-Lopez, A. W.

Rollins, J. M. Roberts, D. Price, J. Peischl, B. A. Nault, K. H. Møller, D. O. Miller, S. Meinardi, Q. Li, J.-F. Lamarque, A. Kupc, H. G. Kjaergaard, D. Kinnison, J. L. Jimenez, C. M. Jernigan, R. S. Hornbrook, A. Hills, M. Dollner, D. A. Day, C. A. Cuevas, P. Campuzano-Jost, J. B. Burkholder, T. P. Bui, W. H. Brune, S. S. Brown, C. A. Brock, I. Bourgeois, D. R. Blake, E. C. Apel, T. B. Ryerson, Global airborne sampling reveals a previously unobserved dimethyl sulfide oxidation mechanism in the marine atmosphere. *Proc. Natl. Acad. Sci. U.S.A.* **118**, e2113268118 (2021).

98. M. A. Robinson, J. A. Neuman, L. G. Huey, J. M. Roberts, S. S. Brown, P. R. Veres, Temperature-dependent sensitivity of iodide chemical ionization mass spectrometers. *Atmos. Meas. Tech.* **15**, 4295–4305 (2022).

99. T. H. Bertram, J. R. Kimmel, T. A. Crisp, O. S. Ryder, R. L. N. Yatavelli, J. A. Thornton, M. J. Cubison, M. Gonin, D. R. Worsnop, A field-deployable, chemical ionization time-of-flight mass spectrometer. *Atmos. Meas. Tech.* **4**, 1471–1479 (2011).

100. B. H. Lee, F. D. Lopez-Hilfiker, C. Mohr, T. Kurtén, D. R. Worsnop, J. A. Thornton, An iodide-adduct high-resolution time-of-flight chemical-ionization mass spectrometer: Application to atmospheric inorganic and organic compounds. *Environ. Sci. Technol.* **48**, 6309–6317 (2014).

101. Y. Ji, L. G. Huey, D. J. Tanner, Y. R. Lee, P. R. Veres, J. A. Neuman, Y. Wang, X. Wang, A vacuum ultraviolet ion source (VUV-IS) for iodide–chemical ionization mass spectrometry: A substitute for radioactive ion sources. *Atmos. Meas. Tech.* **13**, 3683–3696 (2020).

102. M. Breitenlechner, G. A. Novak, J. A. Neuman, A. W. Rollins, P. R. Veres, A versatile vacuum ultraviolet ion source for reduced pressure bipolar chemical ionization mass spectrometry. *Atmos. Meas. Tech.* **15**, 1159–1169 (2022).

103. J. M. Roberts, J. A. Neuman, S. S. Brown, P. R. Veres, M. M. Coggon, C. E. Stockwell, C. Warneke, J. Peischl, M. A. Robinson, Furoyl peroxynitrate (fur-PAN), a product of VOC–NO_x photochemistry from biomass burning emissions: Photochemical synthesis, calibration, chemical characterization, and first atmospheric observations. *Environ. Sci. Atmos.* **2**, 1087–1100 (2022).

104. E. J. Maier, A. C. Aikin, J. E. Ainsworth, Stratospheric nitric oxide and ozone measurements using photoionization mass spectrometry and UV absorption. *Geophys. Res. Lett.* **5**, 37–40 (1978).

105. J. de Gouw, C. Warneke, Measurements of volatile organic compounds in the Earth's atmosphere using proton-transfer-reaction mass spectrometry. *Mass Spectrom. Rev.* **26**, 223–257 (2007).

106. B. Yuan, A. R. Koss, C. Warneke, M. Coggon, K. Sekimoto, J. A. de Gouw, Proton-transfer-reaction mass spectrometry: Applications in atmospheric sciences. *Chem. Rev.* **117**, 13187–13229 (2017).

107. J. Krechmer, F. Lopez-Hilfiker, A. Koss, M. Hutterli, C. Stoermer, B. Deming, J. Kimmel, C. Warneke, R. Holzinger, J. Jayne, D. Worsnop, K. Fuhrer, M. Gonin, J. de Gouw, Evaluation of a new reagent-ion source and focusing ion–molecule reactor for use in proton-transfer-reaction mass spectrometry. *Anal. Chem.* **90**, 12011–12018 (2018).

108. E. R. Crosson, A cavity ring-down analyzer for measuring atmospheric levels of methane, carbon dioxide, and water vapor. *Appl. Phys. B* **92**, 403–408 (2008).

109. C. Rella, J. Hoffnagle, D. Kim-Hak, "Quantification of atmospheric formaldehyde by near-infrared cavity ring-down spectroscopy," in *20th EGU General Assembly, EGU2018, Proceedings from the Conference* (European Geosciences Union, 2017), p. 11101.

110. W. Junkermann, U. Platt, A. Volz-Thomas, A photoelectric detector for the measurement of photolysis frequencies of ozone and other atmospheric molecules. *J. Atmos. Chem.* **8**, 203–227 (1989).

111. W. Zheng, F. M. Flocke, G. S. Tyndall, A. Swanson, J. J. Orlando, J. M. Roberts, L. G. Huey, D. J. Tanner, Characterization of a thermal decomposition chemical ionization mass spectrometer for the measurement of peroxy acyl nitrates (PANs) in the atmosphere. *Atmos. Chem. Phys.* **11**, 6529–6547 (2011).

112. H. Guo, P. Campuzano-Jost, B. A. Nault, D. A. Day, J. C. Schroder, D. Kim, J. E. Dibb, M. Dollner, B. Weinzierl, J. L. Jimenez, The importance of size ranges in aerosol instrument intercomparisons: A case study for the Atmospheric Tomography Mission. *Atmos. Meas. Tech.* **14**, 3631–3655 (2021).

113. R. Bahreini, E. J. Dunlea, B. M. Matthew, C. Simons, K. S. Docherty, P. F. DeCarlo, J. L. Jimenez, C. A. Brock, A. M. Middlebrook, Design and operation of a pressure-controlled inlet for airborne sampling with an aerodynamic aerosol lens. *Aerosol Sci. Tech.* **42**, 465–471 (2008).

114. R. Bahreini, B. Ervens, A. M. Middlebrook, C. Warneke, J. A. De Gouw, P. F. DeCarlo, J. L. Jimenez, C. A. Brock, J. A. Neuman, T. B. Ryerson, H. Stark, E. Atlas, J. Brioude, A. Fried, J. S. Holloway, J. Peischl, D. Richter, J. Walega, P. Weibring, A. G. Wollny, F. C. Fehsenfeld, Organic aerosol formation in urban and industrial plumes near Houston and Dallas, Texas. *J. Geophys. Res. Atmos.* **114**, 2008JD011493 (2009).

115. K. D. Froyd, D. M. Murphy, C. A. Brock, P. Campuzano-Jost, J. E. Dibb, J.-L. Jimenez, A. Kupc, A. M. Middlebrook, G. P. Schill, K. L. Thornhill, C. J. Williamson, J. C. Wilson, L. D. Ziemba, A new method to quantify mineral dust and other aerosol species from aircraft platforms using single-particle mass spectrometry. *Atmos. Meas. Tech.* **12**, 6209–6239 (2019).

116. J. L. Jacquot, X. Shen, M. Abou-Ghanem, K. D. Froyd, M. Lawler, G. P. Schill, K. Slovacek, D. S. Thomson, D. J. Cziczo, D. M. Murphy, A new airborne single particle mass spectrometer: PALMS-NG. *Aerosol Sci. Tech.* **58**, 991–1007 (2024).

117. C. A. Brock, F. Schröder, B. Kärcher, A. Petzold, R. Busen, M. Fiebig, Ultrafine particle size distributions measured in aircraft exhaust plumes. *J. Geophys. Res. Atmos.* **105**, 26555–26567 (2000).

118. C. Williamson, A. Kupc, J. Wilson, D. W. Gesler, J. M. Reeves, F. Erdesz, R. McLaughlin, C. A. Brock, Fast time response measurements of particle size distributions in the 3–60 nm size range with the nucleation mode aerosol size spectrometer. *Atmos. Meas. Tech.* **11**, 3491–3509 (2018).

119. A. Kupc, C. Williamson, N. L. Wagner, M. Richardson, C. A. Brock, Modification, calibration, and performance of the ultra-high sensitivity aerosol spectrometer for particle size distribution and volatility measurements during the Atmospheric Tomography Mission (ATom) airborne campaign. *Atmos. Meas. Tech.* **11**, 369–383 (2018).

120. C. A. Brock, C. Williamson, A. Kupc, K. D. Froyd, F. Erdesz, N. Wagner, M. Richardson, J. P. Schwarz, R.-S. Gao, J. M. Katich, P. Campuzano-Jost, B. A. Nault, J. C. Schroder, J. L. Jimenez, B. Weinzierl, M. Dollner, T. Bui, D. M. Murphy, Aerosol size distributions during the Atmospheric Tomography Mission (ATom): Methods, uncertainties, and data products. *Atmos. Meas. Tech.* **12**, 3081–3099 (2019).

121. C. A. Brock, K. D. Froyd, M. Dollner, C. J. Williamson, G. Schill, D. M. Murphy, N. J. Wagner, A. Kupc, J. L. Jimenez, P. Campuzano-Jost, B. A. Nault, J. C. Schroder, D. A. Day, D. J. Price, B. Weinzierl, J. P. Schwarz, J. M. Katich, S. Wang, L. Zeng, R. Weber, J. Dibb, E. Scheuer, G. S. Diskin, J. P. DiGangi, T. Bui, J. M. Dean-Day, C. R. Thompson, J. Peischl, T. B. Ryerson, I. Bourgeois, B. C. Daube, R. Commane, S. C. Wofsy, Ambient aerosol properties in the remote atmosphere from global-scale in situ measurements. *Atmos. Chem. Phys.* **21**, 15023–15063 (2021).

122. W. Hu, B. B. Palm, D. A. Day, P. Campuzano-Jost, J. E. Krechmer, Z. Peng, S. S. De Sá, S. T. Martin, M. L. Alexander, K. Baumann, L. Hacker, A. Kiendler-Scharr, A. R. Koss, J. A. De Gouw, A. H. Goldstein, R. Seco, S. J. Sjostedt, J.-H. Park, A. B. Guenther, S. Kim, F. Canonaco, A. S. H. Prévôt, W. H. Brune, J. L. Jimenez, Volatility and lifetime against OH heterogeneous reaction of ambientisoprene-epoxydiols-derived secondary organic aerosol (IEPOX-SOA). *Atmos. Chem. Phys.* **16**, 11563–11580 (2016).

123. E. A. Marais, D. J. Jacob, J. R. Turner, L. J. Mickley, Evidence of 1991–2013 decrease of biogenic secondary organic aerosol in response to SO_2 emission controls. *Environ. Res. Lett.* **12**, 054018 (2017).

124. K. T. Vasquez, J. D. Crounse, B. C. Schulze, K. H. Bates, A. P. Teng, L. Xu, H. M. Allen, P. O. Wennberg, Rapid hydrolysis of tertiary isoprene nitrate efficiently removes NO_x from the atmosphere. *Proc. Natl. Acad. Sci. U.S.A.* **117**, 33011–33016 (2020).

125. G. M. Wolfe, M. R. Marvin, S. J. Roberts, K. R. Travis, J. Liao, The Framework for 0-D Atmospheric Modeling (F0AM) v3.1. *Geosci. Model Dev.* **9**, 3309–3319 (2016).

126. Q. Zhu, R. H. Schwantes, M. Coggon, C. Harkins, J. Schnell, J. He, H. O. T. Pye, M. Li, B. Baker, Z. Moon, R. Ahmadov, E. Y. Pfannerstill, B. Place, P. Wooldridge, B. C. Schulze, C. Arata, A. Bucholtz, J. H. Seinfeld, C. Warneke, C. E. Stockwell, L. Xu, K. Zuraski, M. A. Robinson, J. A. Neuman, P. R. Veres, J. Peischl, S. S. Brown, A. H. Goldstein, R. C. Cohen, B. C. McDonald, A better representation of volatile organic compound chemistry in WRF-Chem and its impact on ozone over Los Angeles. *Atmos. Chem. Phys.* **24**, 5265–5286 (2024).

127. F. Li, X. Zhang, S. Kondragunta, X. Lu, I. Csiszar, C. C. Schmidt, Hourly biomass burning emissions product from blended geostationary and polar-orbiting satellites for air quality forecasting applications. *Remote Sens. Environ.* **281**, 113237 (2022).

128. C. E. Stockwell, M. M. Bela, M. M. Coggon, G. I. Gkatzelis, E. Wiggins, E. M. Gargulinski, T. Shingler, M. Fenn, D. Griffin, C. D. Holmes, X. Ye, P. E. Saide, I. Bourgeois, J. Peischl, C. C. Womack, R. A. Washenfelder, P. R. Veres, J. A. Neuman, J. B. Gilman, A. Lamplugh, R. H. Schwantes, S. A. McKeen, A. Wisthaler, F. Piel, H. Guo, P. Campuzano-Jost, J. L. Jimenez, A. Fried, T. F. Hanisco, L. G. Huey, A. Perring, J. M. Katich, G. S. Diskin, J. B. Nowak, T. P. Bui, H. S. Halliday, J. P. DiGangi, G. Pereira, E. P. James, R. Ahmadov, C. A. McLinden, A. J. Soja, R. H. Moore, J. W. Hair, C. Warneke, Airborne emission rate measurements validate remote sensing observations and emission inventories of Western U.S. Wildfires. *Environ. Sci. Technol.* **56**, 7564–7577 (2022).

129. J. He, C. Harkins, K. O'Dell, M. Li, C. Francoeur, K. C. Aikin, S. Anenberg, B. Baker, S. S. Brown, M. M. Coggon, G. J. Frost, J. B. Gilman, S. Kondragunta, A. Lamplugh, C. Lyu, Z. Moon, B. R. Pierce, R. H. Schwantes, C. E. Stockwell, C. Warneke, K. Yang, C. R. Nowlan, G. González Abad, B. C. McDonald, COVID-19 perturbation on US air quality and human health impact assessment. *PNAS Nexus* **3**, pgad483 (2023).

130. J. D. Fast, W. I. Gustafson, R. C. Easter, R. A. Zaveri, J. C. Barnard, E. G. Chapman, G. A. Grell, S. E. Peckham, Evolution of ozone, particulates, and aerosol direct radiative forcing in the

vicinity of Houston using a fully coupled meteorology-chemistry-aerosol model. *J. Geophys. Res. Atmos.* **111**, 10.1029/2005JD006721 (2006).

131. G. A. Grell, S. E. Peckham, R. Schmitz, S. A. McKeen, G. Frost, W. C. Skamarock, B. Eder, Fully coupled "online" chemistry within the WRF model. *Atmos. Environ.* **39**, 6957–6975 (2005).

132. L. K. Emmons, R. H. Schwantes, J. J. Orlando, G. Tyndall, D. Kinnison, J. Lamarque, D. Marsh, M. J. Mills, S. Tilmes, C. Bardeen, R. R. Buchholz, A. Conley, A. Gettelman, R. Garcia, I. Simpson, D. R. Blake, S. Meinardi, G. Pétron, The Chemistry Mechanism in the Community Earth System Model Version 2 (CESM2). *J. Adv. Model Earth Syst.* **12**, 10.1029/2019MS001882 (2020).

133. S. Koplitz, H. Simon, B. Henderson, J. Liljegren, G. Tonnesen, A. Whitehill, B. Wells, Changes in ozone chemical sensitivity in the United States from 2007 to 2016. *ACS Environ. Au* **2**, 206–222 (2022).

134. I. Bourgeois, J. Peischl, C. R. Thompson, K. C. Aikin, T. Campos, H. Clark, R. Commane, B. Daube, G. W. Diskin, J. W. Elkins, R.-S. Gao, A. Gaudel, E. J. Hintsa, B. J. Johnson, R. Kivi, K. McKain, F. L. Moore, D. D. Parrish, R. Querel, E. Ray, R. Sánchez, C. Sweeney, D. W. Tarasick, A. M. Thompson, V. Thouret, J. C. Witte, S. C. Wofsy, T. B. Ryerson, Global-scale distribution of ozone in the remote troposphere from the ATom and HIPPO airborne field missions. *Atmos. Chem. Phys.* **20**, 10611–10635 (2020).

135. I. B. Pollack, J. Lindaas, J. R. Roscioli, M. Agnese, W. Permar, L. Hu, E. V. Fischer, Evaluation of ambient ammonia measurements from a research aircraft using a closed-path QC-TILDAS operated with active continuous passivation. *Atmos. Meas. Tech.* **12**, 3717–3742 (2019).

136. A. P. Sullivan, R. P. Pokhrel, Y. Shen, S. M. Murphy, D. W. Toohey, T. Campos, J. Lindaas, E. V. Fischer, J. L. Collett Jr., Examination of brown carbon absorption from wildfires in the western US during the WE-CAN study. *Atmos. Chem. Phys.* **22**, 13389–13406 (2022).

137. A. P. Sullivan, H. Guo, J. C. Schroder, P. Campuzano-Jost, J. L. Jimenez, T. Campos, V. Shah, L. Jaeglé, B. H. Lee, F. D. Lopez-Hilfiker, J. A. Thornton, S. S. Brown, R. J. Weber, Biomass burning markers and residential burning in the WINTER aircraft campaign. *J. Geophys. Res. Atmos.* **124**, 1846–1861 (2019).

138. G. S. Diskin, J. R. Podolske, G. W. Sachse, T. A. Slate, Open-path airborne tunable diode laser hygrometer. *Proc. SPIE* **4817**, 196 (2002).

139. B. Bohn, I. Lohse, Calibration and evaluation of CCD spectroradiometers for ground-based and airborne measurements of spectral actinic flux densities. *Atmos. Meas. Tech.* **10**, 3151–3174 (2017).

140. L. Lee, A. P. Teng, P. O. Wennberg, J. D. Crounse, R. C. Cohen, On rates and mechanisms of OH and O_3 reactions with isoprene-derived hydroxy nitrates. *J. Phys. Chem. A* **118**, 1622–1637 (2014).

141. J. M. St. Clair, J. C. Rivera-Rios, J. D. Crounse, H. C. Knap, K. H. Bates, A. P. Teng, S. Jørgensen, H. G. Kjaergaard, F. N. Keutsch, P. O. Wennberg, Kinetics and products of the reaction of the first-generation isoprene hydroxy hydroperoxide (ISOPOOH) with OH. *J. Phys. Chem. A* **120**, 1441–1451 (2016).

142. K. H. Bates, J. D. Crounse, J. M. St. Clair, N. B. Bennett, T. B. Nguyen, J. H. Seinfeld, B. M. Stoltz, P. O. Wennberg, Gas phase production and loss of isoprene epoxydiols. *J. Phys. Chem. A* **118**, 1237–1246 (2014).
